# Dysregulation of microRNA Modulatory Network in Abdominal Aortic Aneurysm

**DOI:** 10.3390/jcm9061974

**Published:** 2020-06-24

**Authors:** Daniel P. Zalewski, Karol P. Ruszel, Andrzej Stępniewski, Dariusz Gałkowski, Jacek Bogucki, Łukasz Komsta, Przemysław Kołodziej, Paulina Chmiel, Tomasz Zubilewicz, Marcin Feldo, Janusz Kocki, Anna Bogucka-Kocka

**Affiliations:** 1 Chair and Department of Biology and Genetics, Medical University of Lublin, 4a Chodźki St., 20-093 Lublin, Poland; daniel.piotr.zalewski@gmail.com (D.P.Z.); pachmiel13@gmail.com (P.C.); 2 Chair of Medical Genetics, Department of Clinical Genetics, Medical University of Lublin, 11 Radziwiłłowska St., 20-080 Lublin, Poland; karol.ruszel@umlub.pl (K.P.R.); jacek.bogucki@umlub.pl (J.B.); janusz.kocki@umlub.pl (J.K.); 3 Ecotech Complex Analytical and Programme Centre for Advanced Environmentally Friendly Technologies, University of Marie Curie-Skłodowska, 39 Głęboka St., 20-612 Lublin, Poland; andrzej.stepniewski@umcs.pl; 4 Department of Pathology and Laboratory Medicine, Rutgers - Robert Wood Johnson Medical School, One Robert Wood Johnson Place, New Brunswick, NJ 08903-0019, USA; galkowd@fastmail.fm; 5 Chair and Department of Medicinal Chemistry, Medical University of Lublin, 4 Jaczewskiego St., 20-090 Lublin, Poland; lukasz.komsta@umlub.pl; 6 Laboratory of Diagnostic Parasitology, Chair and Department of Biology and Genetics, Medical University of Lublin, 4a Chodźki St., 20-093 Lublin, Poland; przemyslaw.kolodziej@umlub.pl; 7 Chair and Department of Vascular Surgery and Angiology, Medical University of Lublin, 11 Staszica St., 20-081 Lublin, Poland; tomasz.zubilewicz@umlub.pl (T.Z.); martinf@interia.pl (M.F.)

**Keywords:** abdominal aortic aneurysm, AAA, miRNA, microRNA, gene, expression, next generation sequencing, biomarker

## Abstract

Abdominal artery aneurysm (AAA) refers to abdominal aortic dilatation of 3 cm or greater. AAA is frequently underdiagnosed due to often asymptomatic character of the disease, leading to elevated mortality due to aneurysm rupture. MiRNA constitute a pool of small RNAs controlling gene expression and is involved in many pathologic conditions in human. Targeted panel detecting altered expression of miRNA and genes involved in AAA would improve early diagnosis of this disease. In the presented study, we selected and analyzed miRNA and gene expression signatures in AAA patients. Next, generation sequencing was applied to obtain miRNA and gene-wide expression profiles from peripheral blood mononuclear cells in individuals with AAA and healthy controls. Differential expression analysis was performed using DESeq2 and uninformative variable elimination by partial least squares (UVE-PLS) methods. A total of 31 miRNAs and 51 genes were selected as the most promising biomarkers of AAA. Receiver operating characteristics (ROC) analysis showed good diagnostic ability of proposed biomarkers. Genes regulated by selected miRNAs were determined in silico and associated with functional terms closely related to cardiovascular and neurological diseases. Proposed biomarkers may be used for new diagnostic and therapeutic approaches in management of AAA. The findings will also contribute to the pool of knowledge about miRNA-dependent regulatory mechanisms involved in pathology of that disease.

## 1. Introduction

Abdominal aortic aneurysms (AAA) are segmental dilatations of the abdominal aorta measuring 50% greater than the proximal normal segment, or >3 cm in maximum diameter [1,2]. Screening programs launched in different populations reported the prevalence of AAA between 4% and 8% in general population of men aged 65–80 years [3] and lower (0.45%) in Asians [4]. AAA rupture is responsible for 0.3–0.4% of all death cases and approximately 1% of deaths among men above 65 years globally, causing 130,000 to 180,000 fatalities per year [5]. Although mortality of AAA is decreasing in the 21st Century in many countries including United States and United Kingdom (mostly due to introduction of more advanced endovascular and open surgery repair techniques and better risk factor management), in other countries (Hungary, Romania), AAA mortality is still increasing [6,7,8,9].

The specific mechanism initiating and leading to progression of AAA has not yet been elucidated; however, AAA development has been associated with a variety of infections like brucellosis, salmonellosis and tuberculosis, trauma and connective tissue disorders, Takayasu disease and Marfan syndrome. Identified risk factors for aneurysm development include older age, male gender, cigarette smoking, obesity, dysregulation of lipid levels, hypertension [2,10,11,12] and genetic predisposition [13,14,15,16].

Patients with AAA may report nonspecific symptoms like abdominal and back pain; however, in many cases disease progress is asymptomatic. The prolonged course of asymptomatic phase of AAA provides a relatively long diagnostic window before rupture [17]. Despite many studies targeting circulatory biomarkers of AAA, there is still lack of robust molecular methods able to classify affected individuals with satisfactory precision [18,19,20].

MiRNA-dependent regulation of gene expression emerged as a new tool providing novel opportunities in diagnosis of AAA. MiRNAs are approximately 18–25 nucleotides long, non-coding and single-stranded RNAs, which exhibit gene expression regulating effect by binding to mRNA. The pairing effect of miRNA-mRNA interactions predominantly inhibits gene expression by repression of translation, destabilization and cleavage of mRNA [21,22]. Currently miRNAs are a particularly intensively studied with promising preliminary results opening door to novel diagnostic and treatment approaches [23].

Large studies comparing miRNA and gene expression patterns in patients with AAA and healthy individuals may provide novel biomarkers with good discriminative value improving our diagnostic capability of detecting aneurysm, its rates of progression and complications; however, their introduction to clinical practice requires further investigations [24,25,26,27].

Although differential expression of miRNAs in human AAA cases was reported in abdominal aortic tissue, whole blood, serum and plasma samples, deregulation of miRNA expression in PBMCs (peripheral blood mononuclear cells) was not extensively studied.

In the present study, we applied next generation sequencing to analyze miRNA and gene expression in PBMCs of AAA patients and healthy volunteers with a goal to find the most capable miRNA and gene expression biomarkers of AAA and to research a potential role of identified biomarkers in pathogenesis of AAA.

The study design, methodology, article structure and language have been inspired by our previous studies regarding deregulation of miRNA regulatory network in lower extremities arterial disease [28] and chronic venous disease [29].

## 2. Materials and Methods

### 2.1. Study Participants Characteristics

The study was performed in accordance with the Declaration of Helsinki. The study design was approved by the Ethics Committee of Medical University of Lublin (decision No. K × 10−254/341/2015). Inclusion was carried out between February 2016 and May 2017. Twenty eight patients hospitalized due to intrarenal true AAA in Independent Public Clinical Hospital No. 1 in Lublin were included in the AAA group. All patients underwent pre-operative aneurysm surveillance, which included duplex ultrasonography and contrast enhanced spiral computed tomography with volume-rendered reconstructions.

Nineteen healthy and non-smoking volunteers were included in the control group. Control subjects have not shown presence of abdominal aorta dilatation and abnormalities during duplex ultrasound scanning.

Informed and signed consent was obtained from all study participants. All participants were asked about smoking habits and medical history to establish exclusion criteria, which included presence of inflammatory aneurysm, false aneurysm, thoracic aorta aneurysm, isolated popliteal or iliac artery aneurysm, aortic and/or arterial dissection, stroke, transient ischemic attack (TIA), myocardial infarction, diabetes mellitus type I, symptomatic peripheral arterial disease (ankle brachial index < 0.8), connective tissue disorders including rheumatoid disease, impaired hepatic or renal function, corticoid therapy, infection within previous 6 weeks, recent deep venous thrombosis (less than 1 year), pulmonary embolism, inflammatory and/or infectious disease and cancer. Detailed characteristics of case and control group are presented in Table 1. Application of exclusion criteria enabled to include healthy individuals to control group; however, statistically significant differences in age, body mass index (BMI), smoking habits, and sex distribution were noticed when compared to AAA group (Table 1). Construction of AAA and control groups is described in detail in Appendix A.

### 2.2. Study Material Preparation and Sequencing

The procedure of study material preparation and sequencing was conducted as previously described in [28].

Peripheral blood mononuclear cells (PBMCs) were isolated from whole blood specimens using density gradient centrifugation with Gradisol L reagent (Aqua-Med, Łódź, Poland). Proportions of white blood cells subpopulations in AAA group were obtained from venous blood morphology analysis results and were presented in Figure S1.

Small RNA fractions (for miRNA expression analysis) were isolated from PBMCs specimens of twenty eight AAA patients and nineteen control subjects using MirVana microRNA Isolation Kit (Ambion, Austin, TX, USA).

Total RNA specimens (for transcriptome analysis) were isolated from PBMCs samples of seven randomly selected AAA patients and seven randomly selected controls using TRI Reagent Solution (Applied Biosystems, Foster City, CA, USA).

Small RNA and transcriptome libraries were prepared using Ion Total RNA-Seq Kit v2, Magnetic Bead Cleanup Module kit, Ion Xpress RNA-Seq Barcode 01-16 Kit and sequenced on Ion 540 chips (all Life Technologies, Carlsbad, CA, USA) using Ion S5 XL System (Thermo Fisher Scientific, Waltham, MA, USA). Raw sequences of small RNA and transcriptomic libraries were aligned to 2792 human miRNAs from miRBase v21 (http://www.mirbase.org) and to 55,765 genes of hg19 human genome, respectively.

### 2.3. Statistical and Bioinformatical Analysis

Detailed description of methodology applied to statistical and bioinformatical analysis was provided in our previous study [28].

The differences of AAA and control groups in age and BMI were evaluated using two-sided Mann–Whitney *U* test (wilcox.test function in R), and in sex and smoking using Fisher’s exact test (fisher.test function in R).

Statistical analysis of miRNA expression data (resulted from sequencing of small RNA libraries) and gene expression data (resulted from sequencing of transcriptome libraries) was performed using R environment (version 3.5.2, https://www.r-project.org). Analysis was conducted on biological replicates. Differential expression analysis was performed using DESeq2 and UVE-PLS (uninformative variable elimination by partial least squares) [30] methods implemented in DESeq2 1.18.1 (https://bioconductor.org/packages/release/bioc/html/DESeq2.html) [31] and plsVarSel 0.9.3 (https://cran.r-project.org/web/packages/plsVarSel/index.html) [32] packages, respectively. MiRNA and gene transcripts found by DESeq2 method with *p* value < 0.05 after adjustment by Benjamini–Hochberg false discovery rate were considered as statistically significant. UVE-PLS analysis was performed for miRNA and gene expression data using 3 and 2 PLS components, respectively. UVE-PLS analysis was executed with 1,000 iterations and default cut-off threshold.

Visualizations including Venn diagrams, heat-maps and PCA (principal component analysis) plots were prepared using VennDiagram 1.6.20 (https://cran.r-project.org/web/packages/VennDiagram/index.html) [33], pheatmap 1.0.10 (https://cran.r-project.org/web/packages/pheatmap/index.html) and ggplot2 3.2.1 (https://cran.r-project.org/web/packages/ggplot2/index.html) packages, respectively.

Receiver operating characteristics (ROC) analysis was performed using pROC package version 1.12.1 (https://cran.r-project.org/web/packages/pROC/index.html) [34]. Spearman rank correlation test implemented in Hmisc package 4.4-0. (https://cran.r-project.org/web/packages/Hmisc/index.html) was used to perform correlation analysis.

In order to evaluate the diversity of cell subpopulation in PBMCs specimens, the deconvolution of gene expression data was performed using “quanTIseq” [35] and “MCPcounter” [36] methods implemented to immunedeconv 2.0.0 package (https://rdrr.io/github/grst/immunedeconv/) [37].

Interactions between selected miRNAs and genes were identified using multiMiR package 1.2.0 (https://bioconductor.org/packages/release/bioc/html/multiMiR.html) [38]. Obtained interactions formed a regulatory network, which was presented using Cytoscape v3.5.1 software (https://cytoscape.org/) [39].

Functional analysis was performed for genes included in the network using DAVID (Database for Annotation, Visualization and Integrated Discovery) 6.8 database (https://david.ncifcrf.gov/) [40,41] and it’s supporting resources: KEGG (Kyoto Encyclopedia of Genes and Genomes) pathway maps, the Reactome Database of Signaling Pathways, GAD (Genetic Association Database) and GO (Gene Ontology). As a background, default whole *Homo sapiens* genome was applied. All associated terms of KEGG, Reactome and GAD categories were harvested as well as associated GO terms with Expression Analysis Systematic Explorer (EASE) score < 0.05.

## 3. Results

**Figure 1:**
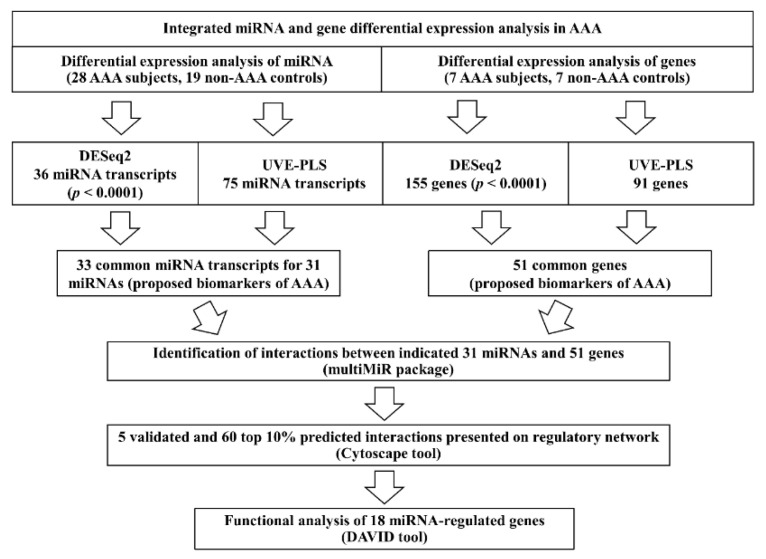
The scheme summarizing applied methodology and general results. AAA—abdominal aortic aneurysm.

The summary of research process is presented on Figure 1.

### 3.1. Study Population Analysis

Study population characteristics (28 patients with AAA and 19 controls) are presented in Table 1. Statistically significant differences between AAA and control groups were noticed in relation to age (*p* = 8.30 × 10^−9^), BMI (*p* = 4.05 × 10^−2^), gender (*p* = 2.63 × 10^−3^) and smoking history (*p* = 6.69 × 10^−3^), resulting from inclusion of healthy, AAA-negative individuals in control group (Table 1, Figure S2).

### 3.2. Primary Results

Results of libraries assessments, Ion Sphere Particles enrichment quality control and results of sequencing data primary analysis are shown in Tables S1 and S2. To assess sequencing data quality, MA plot, boxplot of Cook’s distances across samples and histogram of *p* values frequency were performed for miRNA (Figure S3) and transcriptome (Figure S4) sequencing results.

### 3.3. Differential Expression Analysis of miRNA

Differential expression analysis of miRNA between 28 AAA patients and 19 non-AAA controls was performed using DESeq2 and UVE-PLS methods.

DESeq2 comparative analysis of the miRNA expression signatures revealed 1107 differentially expressed miRNA transcripts in AAA group. Altered expression of 187 miRNA transcripts was characterized by statistical significance (*p* < 0.05) after adjustment by the Benjamini–Hochberg false discovery rate. To limit false positive results, for further comparison with UVE-PLS results we selected 36 differentially expressed miRNA transcripts (for 32 mature miRNAs) with adjusted *p* < 0.0001 (Table S3).

UVE-PLS analysis has returned 75 informative miRNA transcripts (Table S4). The arrangement of prediction error and PLS components as well as cross-validated predictions versus measured values were presented on Figure S5.

**Figure 2:**
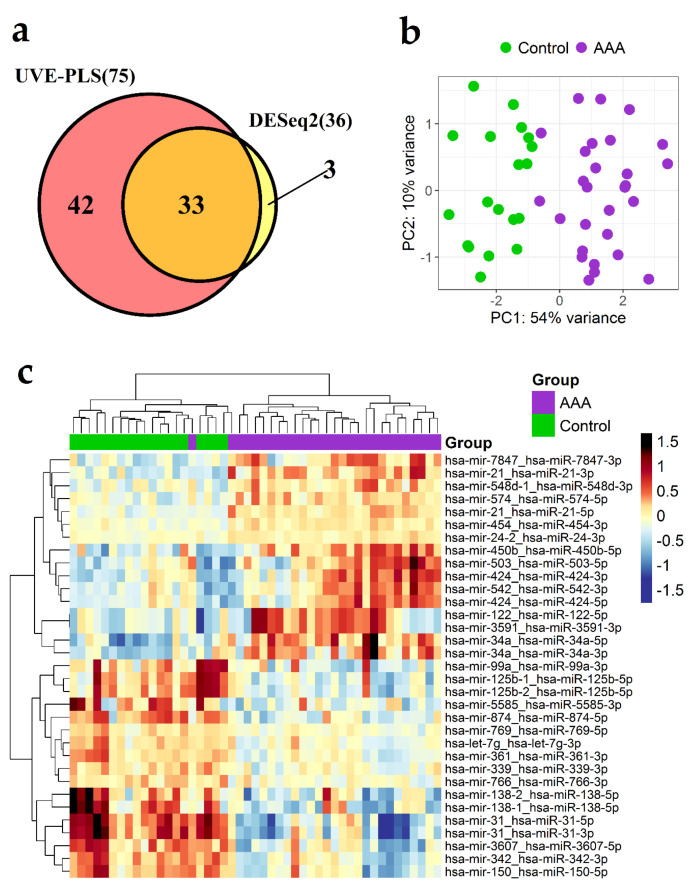
Differential expression analysis of miRNA in PBMCs samples derived from 28 patients with abdominal aortic aneurysm (AAA) and 19 controls (control). (**a**) Venn diagram presenting comparison of two sets of miRNA transcripts: set of 36 miRNA transcripts indicated by DESeq2 analysis with *p* < 0.0001 and set of 75 miRNA transcripts indicated by uninformative variable elimination by partial least squares (UVE-PLS) analysis as informative. A total of 33 miRNA transcripts were common for both analyzed sets. Principal component analysis (PCA) plot (**b**) and heat-map with Euclidean clustering (complete linkage) (**c**) of these common 33 miRNA transcripts.

In the next step, the set of 36 differentially expressed miRNA transcripts (*p* < 0.0001) identified by DESeq2 method and the set of 75 differentially expressed miRNA transcripts identified by UVE-PLS method as informative were compared on Venn diagram (Figure 2a). The comparison disclosed 33 miRNA transcripts selected by both methods (Figure 2a). Differential expression of these 33 miRNA transcripts is visualized on heat-map with Euclidean clustering and PCA plot (Figure 2b,c, respectively).

Discriminative value of altered expression in selected 33 miRNA transcripts was assessed using ROC analysis. Calculated areas under the curve ranged between 0.981 and 0.795, indicating good ability of AAA classification (Table 2 and Table S5, Figure S6). Therefore, a set of 31 mature miRNAs (16 upregulated and 15 downregulated) encoded by selected 33 miRNA transcripts was proposed as the most promising miRNA biomarkers of AAA (Table 2).

### 3.4. Differential Expression Analysis of Genes

Transcriptomic analysis was performed for randomly selected 7 AAA patients and 7 non-AAA controls. Differential expression analysis of genes was performed using DESeq2 and UVE-PLS methods.

DESeq2 analysis revealed 26,816 differentially expressed genes in AAA group when compared to controls. Altered expression of 2238 genes resulted with statistical significance *p* < 0.05, after adjustment by Benjamini–Hochberg false discovery rate. To limit false positive results, a set of 155 differentially expressed genes with adjusted *p* < 0.0001 was chosen for further comparison with UVE-PLS results (Table S6).

UVE-PLS analysis disclosed 91 informative genes, which expression differentiated AAA and control groups (Table S7). Figure S7 presents the arrangement of prediction error and PLS components and also cross-validated predictions versus measured values.

**Figure 3:**
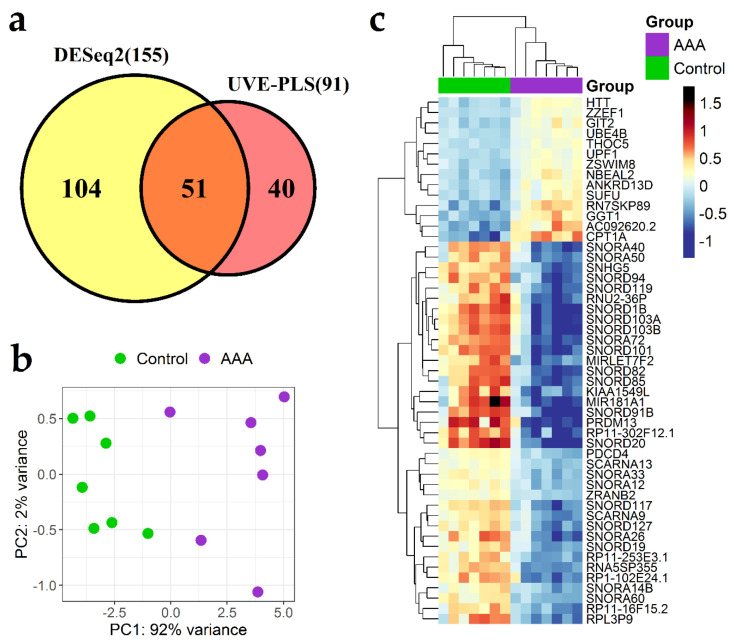
Differential expression analysis of genes in abdominal aortic aneurysm group (AAA) and controls group (control). (**a**) Set of 155 genes indicated by DESeq2 analysis with *p* < 0.0001 and set of 91 genes indicated by uninformative variable elimination by partial least squares (UVE-PLS) analysis were compared on Venn diagram showing 51 common genes. Principal component analysis (PCA) plot (**b**) and heat-map with Canberra clustering (**c**) for expression of common 51 genes.

The comparison between the set of 155 differentially expressed genes revealed by DESeq2 (*p* < 0.0001) and the set of 91 informative genes selected by UVE-PLS disclosed 51 genes common for both methods (Figure 3a). A potential of these 51 genes to differentiate AAA and control groups was evaluated by PCA analysis and Canberra clustering (Figure 3b,c, respectively).

ROC analysis showed strong discriminative value of changed expression of these 51 genes with an area under the ROC curve varying from 0.939 to 1 (Table 3 and Table S8, Figure S8). Therefore, these 51 genes was considered as a panel of transcriptomic biomarkers of AAA (Table 3).

Deconvolution procedure revealed estimated proportions of 11 cell subpopulations in PBMCs specimens subjected to transcriptome analysis. The “quanTIseq” method enables to obtain comparisons between cell types and specimens (Figure S9) and “MCPcounter” method provides further information in differences between samples (Figure S10). Certain differences in proportions of 11 cell subpopulations between samples were noticed; however, further statistics suggests that cell subpopulations composition in PBMCs specimens had no significant influence on the study outcome.

### 3.5. Correlation Analysis

Demographical characteristics (age, BMI), clinical parameters (maximum aneurysm diameter, thrombus volume and aneurysm neck length) and expression data of 34 selected miRNA transcripts and 51 selected genes of AAA group were included to the correlation analysis (Table 4, broaden results are provided in Table S9 and S10). Among demographical and clinical characteristics, statistically significant and weak positive correlation was indicated between age and maximum aneurysm diameter (R = 0.42, p = 0.025), as well as between BMI and thrombus volume (R = 0.38, p = 0.045) (Table S9). Two upregulated miRNAs: hsa-miR-34a-5p and hsa-miR-574-5p were positively correlated with maximum aneurysm diameter and thrombus volume, respectively, what make them potential targets for AAA prognosis. Hsa-miR-769-5p and hsa-miR-7847-3p are associated with age, pointing them as possible age-associated risk factors of AAA. Statistically significant correlations between genes and age (AC092620.2, PDCD4, SNHG5, SUFU, ZRANB2) as well as BMI (GIT2, RP1-102E24.1, RPL3P9) were also revealed (Table 4). Relatively low number of samples does not allow to make categorical conclusions, therefore further studies with larger populations should be performed to confirm these results. To evaluate effects of smoking, coronary artery disease, diabetes mellitus type 2 and hypertension presence in AAA group on miRNA and gene expression, DESeq2 method was applied to find differentially expressed miRNAs and genes in AAA subjects with these conditions in comparison to AAA subjects without them and any of analyzed miRNAs and genes was statistically significantly differentially expressed. This result suggests, that miRNAs and genes identified as potential biomarkers of AAA do not depend on smoking, coronary artery disease, diabetes mellitus type 2 and hypertension; however, this finding should be confirmed in further studies.

### 3.6. In Silico Identification of miRNA:Gene Interactions

**Figure 4:**
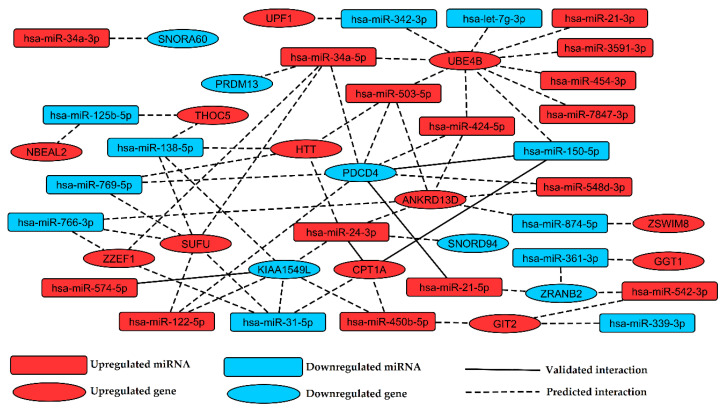
Regulatory network presenting interactions between miRNAs and genes proposed as indicative for abdominal aortic aneurysm.

Identification of validated and predicted miRNA:gene interactions between 31 miRNAs and 51 genes revealed as potential biomarkers of AAA was processed by multiMiR package. In the analysis, five validated miRNA:gene pairs (Table S11) and 60 top 10% predicted miRNA:gene pairs (Table S12) were returned. Received interactions were visualized on regulatory network generated using Cytoscape 3.5.1 software (Figure 4).

### 3.7. Functional Analysis of miRNA Targets

Functional analysis of 18 target genes (*ANKRD13D*, *CPT1A*, *GGT1*, *GIT2*, *HTT*, *KIAA1549L*, *NBEAL2 PDCD4*, *PRDM13*, *SNORA60*, *SNORD94*, *SUFU*, *THOC5*, *UBE4B*, *UPF1*, *ZRANB2*, *ZSWIM8*, and *ZZEF1*) included in the regulatory network was performed using DAVID 6.8 tools and resulted associations are presented in Table 5.

Analyzed genes were associated with cardiovascular diseases (*CPT1A*, *THOC5*, *KIAA1549L*), diabetes (*ANKRD13D*, *CPT1A*), lipids metabolism (*CPT1A*, *GIT2*), inflammation mediators (*GGT1*), glutathione metabolism (*GGT1*), aging (*GGT1*, *PDCD4*), cancer (*HTT*, *SUFU*, *UBE4B*, *PDCD4*), RNA transport and processing (*THOC5*, *UPF1*), proteolysis (*SUFU*, *UBE4B*), chemical dependency (*ZZEF1*, *KIAA1549L*)

Seven out of 18 genes are associated with neurological diseases: Alzheimer’s disease (*CPT1A*, *SUFU*, *ZSWIM8*, *PDCD4*), schizophrenia (*HTT*, *NBEAL2*) and Parkinson disease (*PRDM13*).

GO enrichment analysis assigned upregulated genes (*ANKRD13D*, *CPT1A*, *GGT1*, *GIT2*, *HTT*, *NBEAL2*, *SUFU*, *THOC5*, *UBE4B*, *UPF1*, *ZSWIM8*, and *ZZEF1*) to positive regulation of cellular catabolic process, mRNA transport and developmental processes, while downregulated genes (*KIAA1549L*, *PDCD4*, *PRDM13*, *SNORA60*, *SNORD94*, and *ZRANB2*) were associated with RNA biosynthesis and gene expression (Table 5).

## 4. Discussion

Searching for precise and robust biomarkers of early AAA is crucial due to very subtle symptoms of the disease and high mortality of ruptured aneurysm. Examining deregulations in miRNA network and consequential effects on gene expression appears as an interesting research tactics for finding novel biomarkers of AAA [25,26,27].

In the presented study, we performed integrated analysis of miRNAome and transcriptome expression in PBMC specimens obtained from patients with AAA and healthy controls. The PBMCs pool is involved in inflammation, an important element of AAA pathology, and therefore, should provide an abundance of information about condition and disorders of vascular system. Moreover, high accessibility of PBMCs facilitates translation of obtained results into clinical practice.

Application of next generation sequencing and multi-stage statistical methodology allowed to select 31 miRNAs (Table 2) and 51 genes (Table 3) as the most favorable candidates for detection of AAA (Figure 1). Selection of proposed biomarkers was preceded by control of potential false positive results through adopting the higher threshold of statistical significance (*p* < 0.0001, adjusted by Benjamini–Hochberg false discovery rate) and eliminating uninformative variables using UVE-PLS. High diagnostic value of proposed biomarkers was confirmed in ROC analysis (Table 2 and Table 3, Tables S5 and S8, Figures S6 and S8). Such stringent criteria applied for biomarkers selection were introduced due to the inability to predict in advance the number of miRNA/genes that should be validated by qPCR, thus allowing us not to design proper experiment accordingly.

There is a limited number of studies regarding miRNA expression investigations by next generation sequencing in PBMC samples in AAA individuals [26]. The comparison of our results and findings obtained in similar studies is presented in Table 6. Relatively poor overlap with literature data and our miRNAs list could be explained by differences in methodology applied and biological material subjected to experiments.

In depth discussion regarding functions of DEMs (differentially expressed miRNAs) of this research vastly exceeds capacity of the current paper. Table A1 in Appendix B focuses on most important information regarding possible mechanisms of presented miRNAs actions in AAA. The set of revealed miRNAs clearly points to deregulation of numerous signaling pathways like mTOR, PI3K/AKT, TGF-β, NOTCH, MAPK, and NF-κB. Those affect general processes like cell adhesion, proliferation and motility as well as more detailed ones like wound healing and vascular growth. Taken together, this may point to processes engaged in AAA onset and development like vascular wall remodeling, hypoxia, subsequent revascularization and hemorrhage. On the other hand it is worth to draw attention to selected miRNAs, due to their engagement in regulation of genes, occurring in our sequencing data.

Expression levels of miR-21 may vary between types of biological material. In AAA aortic tissue is significantly upregulated [46] whereas in plasma of AAA patients it may be downregulated [47]. We observed exhibited upregulation of miR-21, suggesting that PBMCs may better reflect processes ongoing in affected aortic tissue. Higher levels of miR-21 were observed in low inflammatory state abdominal aneurysms compared to high inflammation phase aneurysm [48]. On the other hand, elevated level of miR-21 in macrophages promote apoptosis and vascular inflammation in atherogenesis [49] and was shown as a biomarker of low extremities arterial disease [28]. Those facts suggests more detailed assessment of miR-21 function in inflammation, atherogenesis and AAA in the future.

Overexpression of miR-21 leads to downregulation of *PDCD4* and *PTEN* inducing cell proliferation, decreasing apoptosis in the aortic wall, alleviating aneurysm expansion and protect against cell injury caused by hydrogen peroxide exposure [50,51,52]. *PDCD4* is involved in atherosclerosis pathology probably through enhancing levels of IL-6 and IL-8 and promoting apoptosis of VSMC in animal models of coronary atherosclerosis [53]. In our study, both upregulation of miR-21-5p and downregulation of *PDCD4* were observed in PBMCs samples of AAA subjects, suggesting pro-inflammatory and antiapoptotic effects in AAA.

We confirmed findings of Lenk et al. [54] demonstrating upregulation of *GGT1* in patients with AAA. This phenomenon was also reported as a signature of low extremities arterial disease (LEAD) [28], suggesting non-specific character of *GGT1* deregulation.

There was no overlap between our findings and gene expression biomarkers of AAA found in some other studies [55,56,57]. The differences in results may stem from dissimilarities in study material, criteria for participants inclusion and methodological approaches.

Integration of miRNA and gene expression analysis enabled identification of miRNA:gene regulatory pairs in AAA, presented on regulatory network (Figure 4). Elevated expression of *UBE4B* may be caused by many miRNAs selected as biomarkers of AAA (Figure 4) and lead to aneurysm expansion through inhibition of endothelial growth factor receptor (EGFR)-mediated proliferation of vascular cells by enhancing EGFR degradation [58]. Upregulation of *ANKRD13D* may affect endocytic trafficking of EGFR by inhibition of its ubiquitinated form from the cell surface, attenuating pro-proliferative signaling of internalized EGFR [59], potentially aggravating AAA. EGFR signaling might be compromised also by upregulation of miR-424-5p, which is a negative regulator of *EGFR* expression, as observed in tumor cells [60]. According to bioinformatic analysis, this miRNA might be a regulator of both *UBE4B* and *ANKRD13D*, affecting also *EGFR* expression [60]. This net of reciprocal regulations may be considered as a part of a mechanism decreasing cell proliferation in AAA through modulation of EGFR signaling.

The preliminary functional analysis of genes regulated by miRNAs revealed terms closely related to vascular pathology, including lipid metabolism, inflammation, atherosclerosis and aging (Table 5). Interestingly, there were seven genes associated with neurological disorders, including Alzheimer’s disease (*CPT1A*, *SUFU*, *ZSWIM8*, *PDCD4*), schizophrenia (*HTT*, *NBEAL2*) and Parkinson’s disease (*PRDM13*). For further comment on neurological relationships of our findings please refer to Appendix C.

The highly enriched terms associated with genes regulated by biomarker miRNAs like RNA biosynthesis and transport, positive regulation of catabolic processes and developmental processes suggest more general mechanisms also involved in control of gene expression in AAA.

We are aware of limitations of our study design. It was not established, whether alterations in expression of miRNAs and genes proposed as biomarkers were predictive or responsive to AAA development. Although proposed biomarkers were characterized by high predictive value, supported by high level of statistical significance, multistage selection and ROC confirmation, the clinical application requires confirmation in studies with larger cohorts. PBMCs consists of lymphocytes and monocytes subpopulations, varying in miRNA and gene expression patterns. Nuances in proportions of these subpopulations may affect diagnostic value of indicated biomarkers, thus this effect should be further investigated.

Co-existing diseases may bias evaluation of PBMCs expression profiles on a systemic scale. For this reason many conditions were established as exclusion criteria, as mentioned in experimental section. Such strict evaluation helped us to find expression patterns potentially reflecting the local changes in AAA; however, it entailed statistically significant differences in demographic characteristics between AAA and control groups (Table 1). Differences in gender, age and smoking habits might have potentially influenced the study outcome.

The AAA group in our study has men overrepresentation (89.3%, 25 patients) whereas control group is more sex-balanced and include 47% of healthy men (9 subjects) (Table 1). Those characteristics may potentially introduce a gender-associated bias into our data. Some of proposed miRNA biomarkers of AAA have already been connected to sex differences in humans (Table A2 in Appendix D), suggesting that deregulation of these miRNAs may reflect gender differences occurring between AAA and control group. In the case of gene expression, comparison of Deegan et al. paper [61] draw only five overlapping genes, suggesting minor gender bias, while from [62] there were no such ones (Table A3 in Appendix E). Interestingly, Cui et al. discovered that blood is poor material for distinguishing sex associated transcriptome patterns due to relative invariability between genders [63].

The AAA and control groups were age-unmatched (66.39 ± 4.52 years and 36.58 ± 9.97 years, respectively) (Table 1). Literature analysis revealed that some of proposed miRNA biomarkers of AAA may also be considered as senescence indicators (Table A2 in Appendix D). On the other hand, gene expression in AAA has no or little overlap with senescence/aging biomarkers. One of the proposed AAA biomarkers, *SNORA33*, has been previously associated with normal human aging [64]. In a comprehensive transcriptomic analysis of eight senescence in vitro models [65], 20 out of 51 genes reported in our study were also differentially expressed in senescent cells (Table A4 in Appendix F). It could be then possible that senescence characteristics present in our data may be the outcome of general stress(es) due to disease process itself, not only the age.

Smoking is AAA risk factor affecting miRNA expression [66] and in the case of our studies might be another bias-introducing factor, since AAA group includes current smokers (9 persons, 32.1%), while control group is devoid of them (Table 1). Only a small number of miRNAs indicated potential bias after comparison with literature (Table A2 in Appendix D). Other research regarding signatures of smoking did not provide us with any transcriptomic patterns recurring in our data (Table A3 in Appendix E). This suggests an absence of significant smoking-associated bias, probably due to relatively low number of current smokers in AAA group.

Despite of in-depth literature analysis we were unable to exclude unambiguously any miRNAs or gene transcripts from AAA biomarker panels. MiRNAs represent a group of RNAs with pleiotropic regulatory behavior. This does not exclude particular miRNA to act in various cellular processes in different spatiotemporal context. After detailed analysis, we noticed great variability of applied methodologies across literature (refer to literature in Table A3 and Table A4). It is possible that existing discrepancies may reflect methodological rather than sex/age/smoking bias.

Taking all of this into account, it should be considered that presented data shows either real AAA biomarkers or biomarkers associated with both AAA and AAA predisposing factors.

Due to technical (data storage server capacity) and financial limitations, gene expression analysis was performed for 14 out of 47 study participants. It could be a potential source of bias affecting investigations on miRNA:gene regulatory network; however, we confirmed some previously validated interactions (Table S11) and predictive interactions with high probability (Table S12). More research with larger populations is needed to confirm our findings and to validate predictive targets.

The results obtained in our study confirm the important role of miRNA in the pathogenesis of AAA, opening a door to deeper understanding of miRNA functions and regulatory network. AAA biomarkers proposed in this research, after further validation in studies with larger and demographically matched cohorts, can be prospectively applied into clinics for differentiation, diagnosis and therapy of AAA.

## Figures and Tables

**Table 1:** Characteristics of 28 patients with abdominal aortic aneurysm (AAA) and 19 controls included to the study.

Characteristic	AAA Population (n = 28)	Control Population (n = 19)	*p*
Age	66.39 ± 4.52 ^1^ 57–76 ^2^	36.58 ± 9.97 ^1^ 24–55 ^2^	8.30 × 10^−9^
Body Mass Index	25.08 ± 3.30 ^1^ 18.03–31.25 ^2^	23.12 ± 3.93 ^1^ 19.33–32.6 ^2^	4.05 × 10^−2^
Current smoking	9 (32.1%)	0 (0%)	6.69 × 10^−3^
Sex: Male	25 (89.3%)	9 (47%)	2.63 × 10^−3^
Sex: Female	3 (10.7%)	10 (53%)	
**Abdominal aneurysm measurements**			
Maximum aneurysm diameter (cm)	6.389 ± 0.633 ^1^ 5.6–7.8 ^2^	NA	
Thrombus volume (cm^3^)	9.782 ± 3.296 ^1^ 2.9–16.5 ^2^	NA	
Aneurysm neck length (cm)	0.925 ± 0.219 ^1^ 0.5–1.3 ^2^	NA	
**Risk factors and cardiovascular comorbidities**			
Coronary artery disease	7 (25.0%)	NA	
Diabetes type 2	6 (21.4%)	NA	
Hypertension	19 (67.9%)	NA	
**Clinical parameters**			
Red blood cells (M/µL)	4.94 ± 0.21 ^1^ 4.56–5.50 ^2^	NA	
White blood cells (K/µL)	5.66 ± 0.70 ^1^ 4.44–6.90 ^2^	NA	
Platelets (K/µL)	419.93 ± 123.98 ^1^ 211 – 756 ^2^	NA	
Hemoglobin (g/dL)	14.02 ± 0.51 ^1^ 13.34–15.00 ^2^	NA	
Hematocrit (%)	40.75 ± 1.30 ^1^ 38–43 ^2^	NA	
Creatinine (mmol/L)	54.18 ±11.53 ^1^ 39–87 ^2^	NA	
Urea (mmol/L)	4.66 ± 0.67 ^1^ 3.45–5.88 ^2^	NA	
**Medication**			
Statins	13 (46.4%)	NA	
Acetylsalicylic acid	27 (96.4%)	NA	
Clopidogrel	3 (10.7%)	NA	
Beta-adrenergic blockers	16 (57.1%)	NA	
Angiotensin Converting Enzyme Inhibitor	4 (14.3%)	NA	
Ca^2+^ channel blockers	2 (7.14%)	NA	
Fibrates	2 (7.14%)	NA	
Metformin	3 (10.7%)	NA	
Gliclazide	4 (14.3%)	NA	
**Treatment**			
Open surgery	2 (7.14%)	NA	
Stent graft	26 (92.9%)	NA	

^1^ Mean ± SD, ^2^ range. Statistical significance (*p*) of differences between AAA and control group in age and body mass index were determined using two-sided Mann–Whitney *U* test, and in sex and smoking habits were determined using two-sided Fisher’s exact test. AAA—Abdominal Aortic Aneurysm, “NA”—not applicable.

**Table 2:** Set of 33 miRNA transcripts, which significance of differential expression was confirmed by DESeq2 analysis with *p* < 0.0001 and by uninformative variable elimination by partial least squares (UVE-PLS) analysis in patients with abdominal aortic aneurysm in comparison to controls.

No.	miRNA Transcript	miRNA ID^*^	*p*	Fold Change	PLS Coefficient	ROC-AUC
Upregulated miRNA transcripts						
1	hsa-mir-21_hsa-miR-21-5p	hsa-miR-21-5p	9.19 × 10^−12^	1.356	1.61 × 10^−2^	0.953
2	hsa-mir-21_hsa-miR-21-3p	hsa-miR-21-3p	1.73 × 10^−9^	1.704	2.77 × 10^−2^	0.919
3	hsa-mir-34a_hsa-miR-34a-5p	hsa-miR-34a-5p	5.61 × 10^−9^	2.188	4.04 × 10^−2^	0.927
4	hsa-mir-454_hsa-miR-454-3p	hsa-miR-454-3p	2.74 × 10^−8^	1.216	1.15 × 10^−2^	0.940
5	hsa-mir-574_hsa-miR-574-5p	hsa-miR-574-5p	1.13 × 10^−6^	1.364	1.65 × 10^−2^	0.898
6	hsa-mir-424_hsa-miR-424-3p	hsa-miR-424-3p	2.03 × 10^−6^	1.872	2.61 × 10^−2^	0.861
7	hsa-mir-450b_hsa-miR-450b-5p	hsa-miR-450b-5p	2.76 × 10^−6^	1.834	2.54 × 10^−2^	0.872
8	hsa-mir-24-2_hsa-miR-24-3p	hsa-miR-24-3p	8.59 × 10^−6^	1.143	6.77 × 10^−3^	0.874
9	hsa-mir-34a_hsa-miR-34a-3p	hsa-miR-34a-3p	1.42 × 10^−5^	2.357	2.42 × 10^−2^	0.867
10	hsa-mir-542_hsa-miR-542-3p	hsa-miR-542-3p	4.14 × 10^−5^	1.666	1.86 × 10^−2^	0.852
11	hsa-mir-503_hsa-miR-503-5p	hsa-miR-503-5p	6.92 × 10^−5^	1.781	1.99 × 10^−2^	0.821
12	hsa-mir-7847_hsa-miR-7847-3p	hsa-miR-7847-3p	7.00 × 10^−5^	2.270	2.45 × 10^−2^	0.861
13	hsa-mir-548d-1_hsa-miR-548d-3p	hsa-miR-548d-3p	7.10 × 10^−5^	1.493	9.31 × 10^−3^	0.848
14	hsa-mir-122_hsa-miR-122-5p	hsa-miR-122-5p	7.94 × 10^−5^	1.790	1.88 × 10^−2^	0.795
15	hsa-mir-3591_hsa-miR-3591-3p	hsa-miR-3591-3p	7.94 × 10^−5^	1.789	1.88 × 10^−2^	0.795
16	hsa-mir-424_hsa-miR-424-5p	hsa-miR-424-5p	9.56 × 10^−5^	1.579	1.79 × 10^−2^	0.810
**Downregulated miRNA transcripts**						
17	hsa-mir-31_hsa-miR-31-5p	hsa-miR-31-5p	4.18 × 10^−12^	0.344	−4.97 × 10^−2^	0.981
18	hsa-mir-31_hsa-miR-31-3p	hsa-miR-31-3p	4.18 × 10^−12^	0.329	−5.27 × 10^−2^	0.970
19	hsa-mir-874_hsa-miR-874-5p	hsa-miR-874-5p	7.39 × 10^−11^	0.429	−3.33 × 10^−2^	0.934
20	hsa-mir-361_hsa-miR-361-3p	hsa-miR-361-3p	8.26 × 10^−10^	0.683	−1.81 × 10^−2^	0.945
21	hsa-mir-342_hsa-miR-342-3p	hsa-miR-342-3p	1.22 × 10^−7^	0.592	−1.94 × 10^−2^	0.923
22	hsa-mir-138-1_hsa-miR-138-5p	hsa-miR-138-5p	3.65 × 10^−7^	0.368	−4.28 × 10^−2^	0.852
23	hsa-mir-125b-2_hsa-miR-125b-5p	hsa-miR-125b-5p	1.32 × 10^−6^	0.552	−2.56 × 10^−2^	0.868
24	hsa-mir-150_hsa-miR-150-5p	hsa-miR-150-5p	1.88 × 10^−6^	0.581	−2.04 × 10^−2^	0.906
25	hsa-mir-3607_hsa-miR-3607-5p	hsa-miR-3607-5p	2.03 × 10^−6^	0.532	−2.86 × 10^−2^	0.880
26	hsa-mir-769_hsa-miR-769-5p	hsa-miR-769-5p	5.36 × 10^−6^	0.813	−9.44 × 10^−3^	0.874
27	hsa-let-7g_hsa-let-7g-3p	hsa-let-7g-3p	7.34 × 10^−6^	0.750	−1.16 × 10^−2^	0.887
28	hsa-mir-125b-1_hsa-miR-125b-5p	hsa-miR-125b-5p	7.34 × 10^−6^	0.560	−2.35 × 10^−2^	0.857
29	hsa-mir-138-2_hsa-miR-138-5p	hsa-miR-138-5p	2.47 × 10^−5^	0.397	−3.78 × 10^−2^	0.863
30	hsa-mir-339_hsa-miR-339-3p	hsa-miR-339-3p	4.31 × 10^−5^	0.770	−1.00 × 10^−2^	0.868
31	hsa-mir-5585_hsa-miR-5585-3p	hsa-miR-5585-3p	4.64 × 10^−5^	0.396	−1.91 × 10^−2^	0.801
32	hsa-mir-99a_hsa-miR-99a-3p	hsa-miR-99a-3p	6.92 × 10^−5^	0.481	−2.38 × 10^−2^	0.853
33	hsa-mir-766_hsa-miR-766-3p	hsa-miR-766-3p	8.72 × 10^−5^	0.808	−1.63 × 10^−2^	0.852

^1^ According to miRBase 22 (http://www.mirbase.org/). Presented 33 miRNA transcripts give 31 mature miRNAs (miRNA IDs). The table presents *p* (after Benjamini–Hochberg false discovery rate correction) and fold change values resulted from DESeq2 analysis, PLS (partial least squares) coefficients resulted from UVE-PLS (uninformative variable elimination by partial least squares) analysis and areas under ROC (receiver operating characteristics) curves (ROC-area under curves (AUC)) resulted from ROC analysis. MiRNA transcripts were divided into upregulated and downregulated groups and ordered according to increasing *p* value.

**Table 3:** Set of 51 differentially expressed genes, which significance of differential expression was confirmed by DESeq2 analysis with *p* < 0.0001 and by uninformative variable elimination by partial least squares (UVE-PLS) analysis in patients with abdominal aortic aneurysm in comparison to controls.

No.	Gene Symbol	Gene Name	*P*	Fold Change	PLS Coefficient	ROC-AUC
Upregulated Genes						
1	*CPT1A*	carnitine palmitoyltransferase 1A	1.70 × 10^−10^	2.487	1.567 × 10^−3^	1.000
2	*GGT1*	gamma-glutamyltransferase 1	1.11 × 10^−8^	1.973	9.424 × 10^−4^	1.000
3	*UPF1*	UPF1 RNA helicase and ATPase	2.43 × 10^−8^	1.321	4.369 × 10^−4^	1.000
4	*AC092620.2*	Unmatched	8.85 × 10^−7^	2.867	1.239 × 10^−3^	1.000
5	*UBE4B*	ubiquitination factor E4B	5.72 × 10^−6^	1.300	3.839 × 10^−4^	1.000
6	*HTT*	huntingtin	1.12 × 10^−5^	1.388	4.526 × 10^−4^	1.000
7	*NBEAL2*	neurobeachin like 2	1.38 × 10^−5^	1.517	5.590 × 10^−4^	1.000
8	*GIT2*	GIT ArfGAP 2	2.08 × 10^−5^	1.449	5.331 × 10^−4^	1.000
9	*THOC5*	THO complex 5	2.72 × 10^−5^	1.319	4.027 × 10^−4^	1.000
10	*ZZEF1*	zinc finger ZZ-type and EF-hand domain containing 1	2.82 × 10^−5^	1.291	3.325 × 10^−4^	1.000
11	*ANKRD13D*	ankyrin repeat domain 13D	3.01 × 10^−5^	1.428	4.791 × 10^−4^	1.000
12	*SUFU*	SUFU negative regulator of hedgehog signaling	4.11 × 10^−5^	1.482	5.397 × 10^−4^	1.000
13	*RN7SKP89*	RN7SK pseudogene 89	4.45 × 10^−5^	2.764	8.740 × 10^−4^	0.980
14	*ZSWIM8*	zinc finger SWIM-type containing 8	5.52 × 10^−5^	1.355	4.127 × 10^−4^	1.000
**Downregulated genes**						
15	*SNORA60*	small nucleolar RNA, H/ACA box 60	1.19 × 10^−11^	0.547	−1.082 × 10^−3^	1.000
16	*MIRLET7F2*	microRNA let-7f-2	4.89 × 10^−10^	0.285	−1.620 × 10^−3^	1.000
17	*SNHG5*	small nucleolar RNA host gene 5	5.05 × 10^−10^	0.433	−1.296 × 10^−3^	1.000
18	*SNORD20*	small nucleolar RNA, C/D box 20	7.75 × 10^−10^	0.235	−2.069 × 10^−3^	1.000
19	*SNORA72*	small nucleolar RNA, H/ACA box 72	3.72 × 10^−9^	0.358	−1.464 × 10^−3^	1.000
20	*SNORD117*	small nucleolar RNA, C/D box 117	1.11 × 10^−8^	0.457	−1.228 × 10^−3^	1.000
21	*SNORD82*	small nucleolar RNA, C/D box 82	1.17 × 10^−8^	0.357	−1.448 × 10^−3^	1.000
22	*SNORD94*	small nucleolar RNA, C/D box 94	5.10 × 10^−8^	0.387	−1.642 × 10^−3^	1.000
23	*SNORD101*	small nucleolar RNA, C/D box 101	5.43 × 10^−8^	0.330	−1.558 × 10^−3^	1.000
24	*RNA5SP355*	RNA, 5S ribosomal pseudogene 355	8.24 × 10^−8^	0.053	−1.178 × 10^−3^	1.000
25	*SNORD103C (SNORD85)*	small nucleolar RNA, C/D box 103C	1.34 × 10^−7^	0.342	−1.352 × 10^−3^	0.980
26	*RPL3P9*	ribosomal protein L3 pseudogene 9	1.87 × 10^−7^	0.260	−1.237 × 10^−3^	0.980
27	*RP11-16F15.2*	Unmatched	2.06 × 10^−7^	0.344	−1.054 × 10^−3^	1.000
28	*RP11-302F12.1*	Unmatched	2.25 × 10^−7^	0.194	−1.588 × 10^−3^	1.000
29	*SNORA12*	small nucleolar RNA, H/ACA box 12	4.92 × 10^−7^	0.631	−7.161 × 10^−4^	1.000
30	*SNORA33*	small nucleolar RNA, H/ACA box 33	6.82 × 10^−7^	0.633	−7.151 × 10^−4^	1.000
31	*ZRANB2*	zinc finger RANBP2-type containing 2	7.81 × 10^−7^	0.710	−5.094 × 10^−4^	1.000
32	*SNORD91B*	small nucleolar RNA, C/D box 91B	9.72 × 10^−7^	0.324	−1.497 × 10^−3^	1.000
33	*RP11-253E3.1*	Unmatched	1.32 × 10^−6^	0.315	−1.007 × 10^−3^	1.000
34	*SNORD103B*	small nucleolar RNA, C/D box 103B	2.34 × 10^−6^	0.338	−1.417 × 10^−3^	1.000
35	*SNORD127*	small nucleolar RNA, C/D box 127	3.36 × 10^−6^	0.511	−1.002 × 10^−3^	1.000
36	*SNORD103A*	small nucleolar RNA, C/D box 103A	4.06 × 10^−6^	0.354	−1.379 × 10^−3^	1.000
37	*SCARNA13*	small Cajal body-specific RNA 13	4.13 × 10^−6^	0.689	−4.895 × 10^−4^	1.000
38	*SNORA14B*	small nucleolar RNA, H/ACA box 14B	4.66 × 10^−6^	0.592	−7.310 × 10^−4^	1.000
39	*KIAA1549L*	KIAA1549 like	5.44 × 10^−6^	0.178	−1.223 × 10^−3^	0.980
40	*SNORD119*	small nucleolar RNA, C/D box 119	5.58 × 10^−6^	0.427	−1.048 × 10^−3^	1.000
41	*PDCD4*	programmed cell death 4	9.40 × 10^−6^	0.654	−5.442 × 10^−4^	1.000
42	*MIR181A1*	microRNA 181a-1	9.42 × 10^−6^	0.112	−1.680 × 10^−3^	0.980
43	*SCARNA9*	small Cajal body-specific RNA 9	1.14 × 10^−5^	0.539	−8.278 × 10^−4^	1.000
44	*RP1-102E24.1*	Unmatched	1.19 × 10^−5^	0.315	-1.006 × 10^−3^	0.939
45	*PRDM13*	PR/SET domain 13	2.46 × 10^−5^	0.140	−1.674 × 10^−3^	0.959
46	*SNORD19*	small nucleolar RNA, C/D box 19	3.45 × 10^−5^	0.541	−7.859 × 10^−4^	1.000
47	*SNORA26*	small nucleolar RNA, H/ACA box 26	3.67 × 10^−5^	0.425	−1.156 × 10^−3^	1.000
48	*RNU2-36P*	RNA, U2 small nuclear 36, pseudogene	4.80 × 10^−5^	0.401	−9.650 × 10^−4^	0.959
49	*SNORA50A (SNORA50)*	small nucleolar RNA, H/ACA box 50A	4.84 × 10^−5^	0.475	−9.165 × 10^−4^	0.959
50	*SNORA40*	small nucleolar RNA, H/ACA box 40	5.31 × 10^−5^	0.394	−1.075 × 10^−3^	0.959
51	*SNORD1B*	small nucleolar RNA, C/D box 1B	8.82 × 10^−5^	0.333	−1.285 × 10^−3^	0.959

The table presents *p* (FDR with Benjamini–Hochberg correction) and fold change values received from DESeq2 analysis, PLS coefficients received from UVE-PLS analysis and areas under receiver operating characteristics (ROC) curves (ROC-AUC) received from ROC analysis. Genes were divided into upregulated and downregulated groups and ordered according to increasing *p* value. Gene symbols without assigned gene names by a Human Genome Organization (HUGO) Multi-Symbol Checker (https://www.genenames.org/tools/multi-symbol-checker/) were termed as “unmatched”. Gene symbols in brackets are synonyms or previous gene symbols.

**Table 4:** Correlation analysis between maximum aneurysm diameter, thrombus volume, aneurysm neck length, age, body mass index (BMI) and expression of 33 selected miRNA transcripts and 51 selected genes identified as potential abdominal aortic aneurysm signatures. MiRNA transcripts and genes with at least one statistically significant correlation (*p* < 0.05) were presented. All correlations results are provided in Table S9 and S10 in Supplementary File.

miRNA Transcript/Gene	Maximum Aneurysm Diameter		Thrombus Volume		Aneurysm Neck Length		Age		BMI	
	R	*p*	R	*p*	R	*p*	R	*p*	R	*p*
hsa-mir-122_hsa-miR-122-5p	0.10	0.619	0.27	0.160	−0.05	0.782	0.10	0.618	−0.38 ^1^	0.045
hsa-mir-125b-1_hsa-miR-125b-5p	0.02	0.926	−0.19	0.341	0.45 ^1^	0.015	0.08	0.692	−0.01	0.954
hsa-mir-125b-2_hsa-miR-125b-5p	0.12	0.560	−0.08	0.686	0.40 ^1^	0.037	0.09	0.662	0.02	0.901
hsa-mir-34a_hsa-miR-34a-5p	0.47 ^1^	0.011	0.32	0.096	−0.04	0.852	0.26	0.183	-0.01	0.961
hsa-mir-3591_hsa-miR-3591-3p	0.10	0.616	0.27	0.160	−0.05	0.781	0.10	0.617	−0.38 ^1^	0.045
hsa-mir-574_hsa-miR-574-5p	0.16	0.421	0.49 ^1^	0.007	−0.03	0.896	0.26	0.180	0.16	0.414
hsa-mir-769_hsa-miR-769-5p	−0.22	0.252	−0.04	0.832	0.36	0.061	−0.41 ^1^	0.032	0.01	0.973
hsa-mir-7847_hsa-miR-7847-3p	0.33	0.089	−0.01	0.944	0.13	0.521	0.53 ^1^	0.003	-0.03	0.884
*AC092620.2*	0.32	0.482	−0.39	0.389	0.69	0.085	0.81 ^1^	0.028	0.19	0.688
*GIT2*	0.20	0.666	−0.37	0.415	0.64	0.120	0.27	0.563	0.81 ^1^	0.027
*PDCD4*	−0.14	0.768	0.03	0.945	−0.07	0.885	−0.81 ^1^	0.026	−0.18	0.699
*RP1-102E24.1*	0.30	0.508	0.38	0.396	0.08	0.864	−0.18	0.703	−0.78 ^1^	0.039
*RPL3P9*	0.05	0.911	0.14	0.757	−0.42	0.344	−0.17	0.713	−0.76 ^1^	0.046
*SNHG5*	−0.01	0.976	0.32	0.490	−0.22	0.633	−0.80 ^1^	0.030	−0.19	0.685
*SUFU*	0.48	0.278	−0.24	0.597	0.48	0.274	0.77 ^1^	0.041	0.41	0.357
*ZRANB2*	−0.35	0.448	−0.19	0.679	−0.21	0.649	−0.79 ^1^	0.036	0.08	0.857

R—Spearman correlation coefficient, ^1^ correlations statistically significant (*p* < 0.05).

**Table 5:** Functional analysis of eighteen networked miRNA targets.

Functional Analysis of 12 Upregulated Genes (*ANKRD13D*, *CPT1A*, *GGT1*, *GIT2*, *HTT*, *NBEAL2*, *SUFU*, *THOC5*, *UBE4B*, *UPF1*, *ZSWIM8*, and *ZZEF1*)	
*KEGG, Reactome, GAD and GAD Class*	
*ANKRD13D*	**GAD**: Type 2 Diabetes|edema|rosiglitazone **GAD Class**: pharmacogenomic
*CPT1A*	**KEGG**: fatty acid degradation, fatty acid metabolism, PPAR signaling pathway, AMPK signaling pathway, adipocytokine signaling pathway, glucagon signaling pathway, insulin resistance **Reactome**: RORA activates gene expression, PPARA activates gene expression, import of palmitoyl-CoA into the mitochondrial matrix, signaling by retinoic acid **GAD**: acquired immunodeficiency syndrome|disease progression, Alzheimer’s disease, atherosclerosis, BMI–Edema rosiglitazone or pioglitazone, diabetes, type 2 hepatic lipid content insulin, hepatitis C, chronic, hypercholesterolemia|LDLC levels, left ventricular hypertrophy, lipid metabolism, inborn errors|sudden infant death, obesity, tunica media, type 2 diabetes|edema|rosiglitazone **GAD Class**: cardiovascular, infection, metabolic, neurological, pharmacogenomic, unknown
*GGT1*	**KEGG**: taurine and hypotaurine metabolism, cyan amino acid metabolism, glutathione metabolism, arachidonic acid metabolism, metabolic pathways **Reactome**: glutathione synthesis and recycling, synthesis of leukotrienes (LT) and eoxins (EX), aflatoxin activation and detoxification, defective GGT1 causes glutathionuria (GLUTH) **GAD**: aging/ telomere length, alkaline phosphatase, arsenic exposure, cognitive trait, fatty liver|metabolic syndrome X, gamma-glutamyltransferase, liver enzymes, normal variation, pancreatic neoplasm|pancreatic neoplasms, plasma levels of liver enzymes, protein quantitative trait loci, sleep apnea, obstructive **GAD class**: cardiovascular
*GIT2*	**KEGG**: endocytosis **GAD**: cholesterol, HDL, E-selectin **GAD Class**: metabolic
*HTT*	**KEGG**: Huntington’s disease **GAD**: atrophy|Huntington’s disease, chronic progressive chorea|, cognitive ability, cognitive function, Huntington’s disease; ataxia (SCA), myotonic dystrophy type 1, null, Parkinson’s disease, prostatic neoplasms, psychiatric disorders, schizophrenia, sleep disorders; Tourette syndrome, suicide **GAD Class**: cancer, neurological, other, psych, unknown
*NBEAL2*	**GAD**: Schizophrenia **GAD Class**: psych
*SUFU*	**KEGG**: hedgehog signaling pathway, pathways in cancer, Basal cell carcinoma **Reactome**: degradation of GLI1 by the proteasome, Degradation of GLI2 by the proteasome, GLI3 is processed to GLI3R by the proteasome, hedgehog ‘off’ state, hedgehog ‘on’ state **GAD**: Alzheimer’s disease, head and neck neoplasms|neoplasm recurrence, local|neoplasms, second primary **GAD Class**: cancer, neurological
*THOC5*	**KEGG**: RNA transport **Reactome**: transport of mature mRNA derived from an intron-containing transcript, mRNA 3’-end processing **GAD**: carotid atherosclerosis in HIV infection **GAD Class**: cardiovascular
*UBE4B*	**KEGG**: ubiquitin mediated proteolysis, protein processing in endoplasmic reticulum **GAD**: arteries, carcinoma, hepatocellular|hepatitis B, chronic|LCC—liver cell carcinoma|liver neoplasms **GAD Class**: cancer, cardiovascular
*UPF1*	**KEGG**: RNA transport, mRNA surveillance pathway **Reactome**: nonsense mediated decay (NMD) independent of the exon junction complex (EJC), nonsense mediated decay (NMD) enhanced by the exon junction complex (EJC)
*ZSWIM8*	**GAD**: Alzheimer’s disease **GAD Class**: neurological
*ZZEF1*	**GAD**: tobacco use disorder **GAD Class**: chemdependency
*Gene Ontology terms associated with EASE score < 0.05*	
GO Biological Process	Cellular catabolic process, regulation of cellular catabolic process, organic substance catabolic process, catabolic process, regulation of catabolic process, intracellular transport, positive regulation of cellular catabolic process, establishment of localization in cell, positive regulation of catabolic process, cellular response to stimulus, positive regulation of lipid catabolic process, nucleocytoplasmic transport, nuclear transport, cellular localization, cellular developmental process, regulation of lipid catabolic process, behavior, response to stimulus, single-organism intracellular transport, nitrogen compound transport, animal organ development, mRNA-containing ribonucleoprotein complex export from nucleus, mRNA export from nucleus
GO Cellular Compartment	Membrane-bounded organelle, nucleoplasm
**Functional analysis of 6 downregulated gene (*KIAA1549L*, *PDCD4*, *PRDM13*, *SNORA60*, *SNORD94*, and *ZRANB2*)**	
*KEGG, Reactome, GAD and GAD Class*	
*KIAA1549L*	**GAD**: alcoholism, body height, creatinine, heart rate, suicide, attempted **GAD Class**: cardiovascular, chemdependency, developmental, metabolic, psych
*PDCD4*	**KEGG**: proteoglycans in cancer, microRNAs in cancer **GAD**: Alzheimer’s disease, longevity **GAD Class**: aging, neurological
*PRDM13*	**GAD**: menarche, Parkinson’s disease **GAD Class**: neurological, reproduction
*SNORA60*	No information
*SNORD94*	No information
*ZRANB2*	No information
*Gene Ontology terms associated with EASE score <0.05*	
GO Biological Process	Regulation of transcription, DNA-templated, regulation of nucleic acid-templated transcription, regulation of RNA biosynthetic process, regulation of RNA metabolic process, nucleic acid-templated transcription, RNA biosynthetic process, regulation of cellular macromolecule biosynthetic process, regulation of nucleobase-containing compound metabolic process, regulation of macromolecule biosynthetic process, regulation of cellular biosynthetic process, regulation of biosynthetic process, regulation of gene expression, nucleobase-containing compound biosynthetic process, regulation of nitrogen compound metabolic process, heterocycle biosynthetic process, aromatic compound biosynthetic process, organic cyclic compound biosynthetic process, RNA metabolic process, cellular nitrogen compound biosynthetic process, cellular macromolecule biosynthetic process, nucleic acid metabolic process, macromolecule biosynthetic process, gene expression
GO Molecular Function	Nucleic acid binding

Analysis was performed using DAVID 6.8 database and following categories: Kyoto Encyclopedia of Genes and Genomes (KEGG), Reactome, Genetic Association Database (GAD), Genetic Association Database Class (GAD Class) and Gene Ontology (GO).

**Table 6:** The most relevant studies regarding differentially expressed miRNAs in abdominal aortic aneurysm (AAA), with results overlapping findings of the current study.

Ref.	Cases vs Controls	Material	Method (Number of Differentially Expressed miRNAs)	MiRNAs Overlapping with miRNA Biomarkers Proposed in the Current Study
[42]	6 AAA subjects vs 6 controls	Abdominal aorta tissues	qPCR (59)	let-7g-3p, miR-454-3p, -24-3p, -31-5p, -125b-5p, -150-5p, -99a-3p
[43]	169 AAA subjects vs 48 controls	Plasma	qPCR (103)	miR-454-3p, -122-5p, -424-5p, -766-3p
[44]	15 AAA subjects vs 10 non-AAA controls	Whole blood samples	qPCR (29)	miR-125b-5p, -138-5p
[45]	10 AAA subjects vs 10 controls	Plasma	Microarray (151)	miR-21-5p, -574-5p, -24-3p, -122-5p, -31-5p, -342-3p, -150-5p, -125b-5p, -339-3p
[46]	5 AAA subjects vs 5 controls	Infrarenal aortic tissues	Microarray (8)	miR-21-5p

The table presents studies on miRNAs in AAA. Differentially expressed miRNAs are revealed from AAA subjects and control groups. MiRNAs overlapping with biomarkers proposed in the current study are shown. For more comprehensive review of this topic please refer to [26].

## Data Availability

All datasets generated for this study can be found in the FigShare repository (link will be provided upon acceptance).

## Supplementary Materials

The following are available online at https://www.mdpi.com/2077-0383/9/6/1974/s1. Figure S1: proportions in white blood cells subpopulations in AAA patients resulted from blood morphology analysis. Figure S2: evaluation of differences between AAA group (n = 28) and the control group (n = 19) for age (A), BMI (B), sex (C) and smoking (D). Statistical significance (*p* values) were calculated using two-sided Mann–Whitney *U* test (for age and BMI) and a two-sided Fisher’s exact test (for sex and smoking). Distributions of age and BMI were presented on boxplots (A) and (B), respectively, with whiskers defining region between minimum and maximum values, boxes covering values between 25% and 75% quantile and horizontal lines inside boxes marking median value. Distribution of sex (black—female, gray—male) and smoking (black—no-smokers, grey—smokers) were presented on spine plots (C) and (D), respectively. Figure S3: quality control of data obtained from sequencing of small RNA libraries and results of differential expression analysis performed by DESeq2 package in 28 AAA individuals and 19 healthy controls. (A) Boxplot presents Cook’s distances of miRNAs across samples. Whiskers define range between minimum and maximum value of Cook’s distance, boxes range between 25% and 75% quartile, horizontal lines inside boxes mark median value. (B) Histogram presenting distribution of DESeq2 *p* values. (C) MA plot showing relation between log2 of fold changes of differentially expressed miRNAs and averages of normalized counts. MiRNAs with *p* value < 0.1 were marked as red points. Figure S4: quality control of data obtained from sequencing of transcriptome libraries and results of differential expression analysis performed by DESeq2 package between group of 7 AAA individuals and a group of 7 healthy controls. (A) Boxplot presenting Cook’s distances of miRNAs across samples. Whiskers define range between minimum and maximum value of Cook’s distance, boxes range between 25% and 75% quartile, horizontal lines inside boxes mark median value. (B) Histogram presenting distribution of DESeq2 *p* values. (C) MA plot showing relation between log2 of fold changes of differentially expressed miRNAs and averages of normalized counts. MiRNAs with *p* value < 0.1 were marked as red points. Figure S5: UVE-PLS differential expression analysis of miRNA expression data of 28 AAA patients compared to 19 healthy controls. Plots present the arrangement of prediction error and PLS components (A) and cross-validated predictions versus measured values (B). Figure S6: results of receiver operating characteristics (ROC) analysis performed for selected 33 miRNA transcripts differentially expressed in AAA. Values of area under curves (AUC) with 95% confidence interval (in brackets) were included in each plot. Figure S7: UVE-PLS differential expression analysis of transcriptomic expression data of 7 AAA individuals compared to 7 healthy controls. Plots present the arrangement of prediction error and PLS components (A) and cross-validated predictions versus measured values (B). Figure S8: results of receiver operating characteristics (ROC) analysis of 51 genes selected as signatures of AAA. Values of area under curves (AUC) with 95% confidence interval (in brackets) were included in each plot. Figure S9: results of deconvolution procedure performed on gene expression datasets of 7 AAA patients and 7 controls using “quanTIseq” method implemented to immunedeconv 2.0.0 package. Figure S10: results of deconvolution procedure performed on gene expression datasets of 7 AAA patients (AAA) and 7 controls (control) using “MCPcounter” method implemented to immunedeconv 2.0.0 package. Two-sided Mann–Whitney test (wilcox.test function in R) was used to calculate statistical significance of differences in score values between AAA and the control group. Table S1: assessment of small RNA samples, small RNA libraries and results of primary analysis of small RNA sequencing data carried out with Ion Torrent small RNA Plugin v5.0.5r3. Table S2: assessment of transcriptome libraries and results of primary analysis of transcriptome sequencing data carried out with Ion Torrent RNASeqAnalysis plugin v.5.0.3.0. Table S3: the set of 36 differentially expressed miRNA transcripts in the group of 28 AAA individuals compared to 19 healthy controls, resulted from DESeq2 analysis with *p* < 0.0001. MiRNA transcripts were ordered according to increasing *p* value. Table S4: the set of 75 differentially expressed miRNA transcripts in the group of 28 AAA individuals compared to 19 healthy controls, resulted from UVE-PLS analysis. MiRNA transcripts were ordered according to decreasing PLS coefficients. Table S5: results of ROC analysis for 34 miRNA transcripts selected as signatures for AAA. Table S6: results of DESeq2 differential analysis of genes in the group of 7 AAA individuals compared to the group of 7 healthy controls. The table presents 155 differentially expressed genes with *p* < 0.0001, ordered accordingly to increasing *p* value. Gene names were searched using HUGO multi-symbol checker and gene symbols not assigned to names are termed as “unmatched”. Synonyms or previous gene symbols are placed in brackets. Table S7: results of UVE-PLS analysis of miRNA in the group of 7 AAA individuals compared to the group of 7 healthy controls. The table presents 91 differentially expressed genes recognized as informative, ordered according to decreasing PLS coefficients. Gene names were searched using the HUGO Multi-Symbol Checker and gene symbols not assigned to names are termed as “unmatched”. Synonyms or previous gene symbols are placed in brackets. Table S8: results of ROC analysis for 51 genes selected as signatures of AAA. Table S9: correlation analysis between maximum aneurysm diameter, thrombus volume, aneurysm neck length, age, BMI and expression of 33 selected miRNA transcripts identified as potential AAA signatures. Table S10: correlation analysis between maximum aneurysm diameter, thrombus volume, aneurysm neck length, age, BMI and expression of 51 selected genes identified as potential AAA signatures. Table S11: experimentally validated miRNA:gene pairs found in silico among 31 miRNAs and 51 genes indicative for AAA. Table S12: top 10% predicted miRNA:gene pairs obtained in silico among 31 miRNAs and 51 genes found as signatures of AAA.

## Appendix A

### Abdominal Aorta Aneurysm and Control Groups Construction

All 28 patients from analyzed abdominal aorta aneurysm (AAA) group were hospitalized in Department of Vascular Surgery of the Independent Public Clinical Hospital No. 1 in Lublin for AAA as the main diagnosis. The lack of comorbidities was a crucial condition to qualify the patient to the study to create as homogenous group as possible. During the recruitment processes, the medical history and comorbidities were analyzed three times by three different physicians: vascular surgeon, anesthetist (two weeks before admission) and then, one more time, at the day of admission by vascular surgeon in order to conclude on current health state before surgery. In this study, we screened 232 AAA patients during first stage of recruitment. A total of 198 patients was excluded due to chronic venous disease (C2-C6 stage), inflammatory aneurysm, false aneurysm, thoracic aorta aneurysm, isolated popliteal or iliac artery aneurysm, aortic and/or arterial dissection, stroke, transient ischemic attack, myocardial infarction, diabetes mellitus type I, symptomatic peripheral arterial disease (ABI < 0.8), connective tissue disorders including rheumatoid disease, impaired hepatic or renal function, corticoid therapy, infection within previous six weeks, deep venous thrombosis (less than one year), pulmonary embolism, inflammatory and/or infectious disease and cancer. In fact, at the age of 60–70 it is quite impossible to find AAA patients without any kind of comorbidities.

To construct the control group, we aimed to include healthy (confirmed by vascular surgeon) and non-smoking subjects. AAA mainly affects elderly men and often is accompanied with other diseases, what is the reason of presence of differences in age, BMI, gender and smoking between AAA patients and controls. Therefore, it is difficult to find age- and gender-matched control group including healthy subjects and to avoid differences in those features during the study participants’ inclusion. We cope with this biological bias by extension of standard statistical methodology by noise reduction using advanced statistical methods (UVE-PLS), and strict significance thresholds (*p* < 0.0001 for miRNAs and genes). This particular group of subjects was used also in our both previous research as the control group [28,29].

## Appendix B

**Table A1:** Selected most prominent targets and processes affected by differentially expressed miRNAs from presented study, drawn from literature analysis.

MiRNAs Reported in the Present Study as Upregulated in AAA	
miRNA	Remarks
hsa-miR-21	Function in atherogenesis [28,46,49] and AAA [48], targets *PTEN* [50,51,52].
hsa-miR-24	Downregulated in plasma of AAA patients and murine AAA models [67].
hsa-miR-34a	Was deregulated in abdominal aorta tissue of AAA animal models [67].
hsa-miR-122	Role in Alzheimer’s disease through regulation of genes involved in lipid metabolism [68].
hsa-miR-424	Negative regulator of *EGFR* expression in tumor cells [60], targets Rictor (mTOR complex 2 signaling element), promotes tumor progression [69], affects MAPK and focal adhesion signaling pathways in esophageal squamous cell carcinoma [70].
hsa-miR-450b	Affects MAPK and focal adhesion signaling pathways in esophageal squamous cell carcinoma [70].
hsa-miR-454	Directly targets *PTEN* [71], promotes cancer progression [71,72], inhibits Wnt/β-catenin signaling [72].
hsa-miR-503	Targets Rictor (mTOR complex 2 signaling element), promotes tumor progression [69], promotes ESCC cell proliferation, migration, and invasion by targeting cyclin D1 [73], negative regulator of proliferation in primary human cells [74].
hsa-miR-542	Upregulated in AAA patients [42].
hsa-miR-548d	Associated with schizophrenia [75].
hsa-miR-574	Circulating marker of TAA [76], repressor of *VEGFA* [77], promotes VSMCs growth in CAD [78].
hsa-miR-3591	Lower extremities arterial disease-associated miRNA [28].
**MiRNAs reported in the present study as downregulated in AAA**	
hsa-let-7g	Increases viability of lung cancer and osteosarcoma cells via downregulation of *HOXB1* and activation of NF-kB pathway [79,80].
hsa-miR-31	Knockdown of this miRNA inhibits expression of Collagen I and III and Fibronectin in hypertrophic scar formation [81], regulator of senescence in cancer cells [82].
hsa-miR-99a	Significantly decreased in patients with AMI [83], regulates cell migration and cell proliferation by targeting PI3K/AKT and mTOR in wound healing model [84].
hsa-miR-125b	Associated with immune response of patients with ruptured intracranial aneurysms [85], upregulated in AAA subjects [44], suppresses bladder cancer development by targeting *SIRT7* and *MALAT1* [86].
hsa-miR-138	Promotes glioma angiogenesis through miR-138/HIF-1α/VEGF axis [87], upregulated after the induction of myocardial infarction [88].
hsa-miR-150	Inactivates VEGFA/VEGFR2 and the downstream Akt/mTOR signaling pathway in colorectal cancer [89], marker for early diagnosis of AMI [90], underexpression of this miRNA promotes proliferation and metastasis of gastric cancer [91].
hsa-miR-339	Overexpression of this miRNA can inhibit HCC cell invasion [92].
hsa-miR-342	Marker of T2D patients with high risk for developing CAD [93], in hUCMSCs enhances osteogenesis by targeting *SUFU*, induces TGF-β expression [94], regulates cell proliferation and apoptosis in hepatocellular carcinoma through Wnt/β-catenin signaling pathway [95].
hsa-miR-361	Overexpression in cutaneous leishmaniosis lesions, impairs epidermal barrier function by filaggrin-2 repression [96].
hsa-miR-766	Indirectly inhibits of NF-κB signaling causing anti-inflammatory response [97].
hsa-miR-769	Expression is significantly correlated with the presence of pronounced coronary atherosclerosis [98], inhibits colorectal cancer cell proliferation and invasion by targeting *HEY1* (downstream effector of NOTCH signaling pathway) [99], negatively correlated with *EGFR* expression [100].
hsa-miR-874	Decreased expression was associated with poor overall survival of ESCC patients, targets *STAT3* [101].
hsa-miR-5585	Regulates cell cycle progression in human colorectal carcinoma cells, decreases expression of *TGFβ-R1*, *TGFβ-R2*, *SMAD3*, and *SMAD4* [102].

AAA—aortic abdominal aneurysm, AMI—acute myocardial infraction, CAD—coronary artery disease, EGFR—endothelial growth factor receptor, ESCC—esophagal squamous cells carcinoma, HCC—human colorectal cancer, HEY1—hairy/enhancer-of-split related with YRPW motif protein 1, HIF-1α—hypoxia induced factor 1α, HOXB1—homeobox B1, hUCMSCs—human umbilical cord mesenchymal stem cells, MALAT1—metastasis associated lung adenocarcinoma transcript 1, MAPK—mitogen activated protein kinase, mTOR—mammalian target of rapamycin, NF-κB—necrotic factor κB, NOTCH—translocation-associated protein, PI3K/AKT—phosphoinositide 3-kinases/protein kinase B, PTEN—phosphatase and tensin homolog deleted on chromosome ten, T2D—type 2 diabetes, TAA—thoracic aortic aneurysm, TGF-β—tumor growth factor β, TGFβ-R1—tumor growth factor β receptor 1, TGFβ-R2—tumor growth factor β receptor 2, SIRT7—sirtuin 7, SMAD3—decapentaplegic homolog 3, SMAD4—decapentaplegic homolog 4, STAT3—signal transducer and activator of transcription 3, SUFU—suppressor of fused homolog, Wnt—wingless-type MMTV integration site family of genes, VEGF—vascular endothelial growth factor, VEGFA—vascular endothelial growth factor A, VEGFR2—vascular endothelial growth factor receptor 2, VSMCs—vascular smooth muscle cells.

## Appendix C

### Neurological Associations of Genes and miRNAs Involved in Abdominal Aortic Aneurysm (AAA) Pathology

Although our research was focused on AAA, some additional associations with nervous system diseases were discovered. Interestingly, there were seven genes associated with neurological disorders, like Alzheimer disease (*CPT1A*, *SUFU*, *ZSWIM8*, *PDCD4*), schizophrenia (*HTT*, *NBEAL2*) and Parkinson’s disease (*PRDM13*) (Table 5). *SUFU* and *PDCD4* genes were linked to Alzheimer’s disease and were shown as predictive targets of miR-34a-5p. Upregulation of miR-34a-5p was earlier reported in blood mononuclear cells from individuals with Alzheimer’s disease [103]. Both *SUFU* and *PDCD4* were also predictive targets of miR-122-5p involved in development of Alzheimer’s disease through regulation of genes involved in lipid metabolism [68]. Another target for miR-34a-5p was *PRDM13*, which was found to be associated with susceptibility to Parkinson’s disease. Upregulation of miR-34a was reported in in vitro model of Parkinson’s disease [104]. The regulatory network including miR-34a-5p, miR-122-5p, *SUFU*, *PDCD4*, and *PRDM13* may be involved in development of aneurysm-related neurodegenerative diseases. The link between nervous system and cardiovascular diseases is well known and was also confirmed in our earlier research [28].

## Appendix D

**Table A2:** Associations of miRNAs proposed in our study as abdominal aortic aneurysm (AAA) biomarkers with gender and aging, found in the most relevant literature.

**miRNAs**	Previous studies reported association with gender	Previous studies reported association with aging	Previous studies reported association with smoking
**miRNAs reported in the present study as upregulated in AAA**			
hsa-miR-21	[105]	[106]	
hsa-miR-24		[106,107]	
hsa-miR-34a		[108]	
hsa-miR-122		[106]	
hsa-miR-424	[63,105]	[106]	
hsa-miR-450b			
hsa-miR-454	[63]		
hsa-miR-503		[106]	
hsa-miR-542			
hsa-miR-548d		[106]	
hsa-miR-574			[109]
hsa-miR-3591			
**miRNAs reported in the present study as downregulated in AAA**			
hsa-let-7g		[106,107]	
hsa-miR-31			
hsa-miR-99a	[63]	[110]	
hsa-miR-125b		[106]	
hsa-miR-138		[106,107]	[111]
hsa-miR-150	[63,105]		
hsa-miR-339	[63]		
hsa-miR-342	[105]		
hsa-miR-361	[63]		
hsa-miR-766		[106]	[112]
hsa-miR-769	[63]		
hsa-miR-874		[106]	
hsa-miR-5585			

## Appendix E

**Table A3:** Associations of genes proposed in our study as AAA biomarkers with gender and smoking, found in the most relevant literature.

No.	Gene Symbol	Association with Gender		Association with Smoking							
		[61] ^1^	[62]	[113] ^2^	[114] ^3^	[115] ^4^	[116] ^5^	[117] ^5^	[118] ^5^	[119]	[120]
Upregulated genes											
1	*CPT1A*	no	no	no	no	no	no	no	no	no	no
2	*GGT1*	no	no	no	no	no	no	no	no	no	no
3	*UPF1*	yes, f	no	no	no	no	no	no	no	no	no
4	*AC092620.2*	no	no	no	no	no	no	no	no	no	no
5	*UBE4B*	yes, f	no	no	no	no	no	no	no	no	no
6	*HTT*	no	no	no	no	no	no	no	no	no	no
7	*NBEAL2*	no	no	no	no	no	no	no	no	no	no
8	*GIT2*	no	no	no	no	no	no	no	no	no	no
9	*THOC5*	no	no	no	no	no	no	no	no	no	no
10	*ZZEF1*	no	no	no	no	no	no	no	no	no	no
11	*ANKRD13D*	no	no	no	no	no	no	no	no	no	no
12	*SUFU*	no	no	no	no	no	no	no	no	no	no
13	*RN7SKP89*	no	no	no	no	no	no	no	no	no	no
14	*ZSWIM8*	no	no	no	no	no	no	no	no	no	no
**Downregulated genes**											
15	*SNORA60*	no	no	no	no	no	no	no	no	no	no
16	*MIRLET7F2*	no	no	no	no	no	no	no	no	no	no
17	*SNHG5*	no	no	no	no	no	no	no	no	no	no
18	*SNORD20*	no	no	no	no	no	no	no	no	no	no
19	*SNORA72*	no	no	no	no	no	no	no	no	no	no
20	*SNORD117*	no	no	no	no	no	no	no	no	no	no
21	*SNORD82*	yes, m	no	no	no	no	no	no	no	no	no
22	*SNORD94*	no	no	no	no	no	no	no	no	no	no
23	*SNORD101*	no	no	no	no	no	no	no	no	no	no
24	*RNA5SP355*	no	no	no	no	no	no	no	no	no	no
25	*SNORD103C (SNORD85)*	no	no	no	no	no	no	no	no	no	no
26	*RPL3P9*	no	no	no	no	no	no	no	no	no	no
27	*RP11-16F15.2*	no	no	no	no	no	no	no	no	no	no
28	*RP11-302F12.1*	no	no	no	no	no	no	no	no	no	no
29	*SNORA12*	no	no	no	no	no	no	no	no	no	no
30	*SNORA33*	no	no	no	no	no	no	no	no	no	no
31	*ZRANB2*	yes, m	no	no	no	no	no	no	no	no	no
32	*SNORD91B*	no	no	no	no	no	no	no	no	no	no
33	*RP11-253E3.1*	no	no	no	no	no	no	no	no	no	no
34	*SNORD103B*	no	no	no	no	no	no	no	no	no	no
35	*SNORD127*	no	no	no	no	no	no	no	no	no	no
36	*SNORD103A*	no	no	no	no	no	no	no	no	no	no
37	*SCARNA13*	no	no	no	no	no	no	no	no	no	no
38	*SNORA14B*	no	no	no	no	no	no	no	no	no	no
39	*KIAA1549L*	no	no	no	no	no	no	no	no	no	no
40	*SNORD119*	no	no	no	no	no	no	no	no	no	no
41	*PDCD4*	yes, m	no	no	no	no	no	no	no	no	no
42	*MIR181A1*	no	no	no	no	no	no	no	no	no	no
43	*SCARNA9*	no	no	no	no	no	no	no	no	no	no
44	*RP1-102E24.1*	no	no	no	no	no	no	no	no	no	no
45	*PRDM13*	no	no	no	no	no	no	no	no	no	no
46	*SNORD19*	no	no	no	no	no	no	no	no	no	no
47	*SNORA26*	no	no	no	no	no	no	no	no	no	no
48	*RNU2-36P*	no	no	no	no	no	no	no	no	no	no
49	*SNORA50A (SNORA50)*	no	no	no	no	no	no	no	no	no	no
50	*SNORA40*	no	no	no	no	no	no	no	no	no	no
51	*SNORD1B*	no	no	no	no	no	no	no	no	no	no

^1^ Heart tissue gender bias only, ^2^ xenobiotic metabolism genes only, ^3^ CpG island methylation only, full data comparison, ^4^ genes associated with smoking behavior, ^5^ full data comparison, f—female biased, m—male biased, no—there is no association between presented data and literature, yes—there is an association between presented data and literature.

## Appendix F

**Table A4:** Associations of genes proposed in our study as AAA biomarkers with aging, found in the most relevant literature.

No.	Gene Symbol	Associations with Aging:					
		[121] ^1^	[122]	[64]	[123]	[65] ^1^	Remarks for [65]
Upregulated Genes							
1	*CPT1A*	no	no	no	no	yes	IMR90 IR, IMR90 Rep, HUVEC IR, HAEC IR, WI38 Onc
2	*GGT1*	no	no	no	no	yes	WI38 IR, WI38 Onc, WI38 Dox, IMR90 IR, WI38 Rep, HUVEC IR
3	*UPF1*	no	no	no	no	yes	IMR90 Rep, IMR90 IR, WI38 Onc,
4	*AC092620.2*	no	no	no	no	yes	WI38 Onc
5	*UBE4B*	no	no	no	no	yes	IMR90 Rep, IMR90 IR
6	*HTT*	no	no	no	no	yes	IMR90 Rep, WI38 Onc, IMR90 IR
7	*NBEAL2*	no	no	no	no	yes	WI38 Onc, HAEC IR, WI38 Dox
8	*GIT2*	no	no	no	no	yes	IMR90 Rep, WI38 Dox, IMR90 IR, WI38 Onc
9	*THOC5*	no	no	no	no	no	
10	*ZZEF1*	no	no	no	no	no	
11	*ANKRD13D*	no	no	no	no	no	
12	*SUFU*	no	no	no	no	yes	HUVEC IR
13	*RN7SKP89*	no	no	no	no	no	
14	*ZSWIM8*	no	no	no	no	yes	HUVEC IR, IMR90 IR, IMR90 Rep
**Downregulated genes**							
15	*SNORA60*	no	no	no	no	no	
16	*MIRLET7F2*	no	no	no	no	no	
17	*SNHG5*	no	no	no	no	yes	HUVEC IR
18	*SNORD20*	no	no	no	no	no	
19	*SNORA72*	no	no	no	no	no	
20	*SNORD117*	no	no	no	no	no	
21	*SNORD82*	no	no	no	no	no	
22	*SNORD94*	no	no	no	no	yes	WI38 Dox
23	*SNORD101*	no	no	no	no	yes	HAEC IR, WI38 Dox, WI38 Onc, HUVEC IR
24	*RNA5SP355*	no	no	no	no	no	
25	*SNORD103C (SNORD85)*	no	no	no	no	no	
26	*RPL3P9*	no	no	no	no	no	
27	*RP11-16F15.2*	no	no	no	no	yes	WI38 Rep
28	*RP11-302F12.1*	no	no	no	no	no	
29	*SNORA12*	no	no	no	no	no	
30	*SNORA33*	no	no	yes	no	no	
31	*ZRANB2*	no	no	no	no	no	
32	*SNORD91B*	no	no	no	no	no	
33	*RP11-253E3.1*	no	no	no	no	no	
34	*SNORD103B*	no	no	no	no	no	
35	*SNORD127*	no	no	no	no	no	
36	*SNORD103A*	no	no	no	no	no	
37	*SCARNA13*	no	no	no	no	no	
38	*SNORA14B*	no	no	no	no	yes	WI38 Dox, WI38 Onc
39	*KIAA1549L*	no	no	no	no	yes	WI38 Dox, IMR90 Rep, IMR90 IR, WI38 IR, WI38 Onc
40	*SNORD119*	no	no	no	no	no	
41	*PDCD4*	no	no	no	no	yes	WI38 Onc, HUVEC IR, HAEC IR, WI38 Dox, WI38 Rep
42	*MIR181A1*	no	no	no	no	no	
43	*SCARNA9*	no	no	no	no	yes	WI38 Onc, HUVEC IR, IMR90 Rep, IMR90 IR
44	*RP1-102E24.1*	no	no	no	no	no	
45	*PRDM13*	no	no	no	no	no	
46	*SNORD19*	no	no	no	no	yes	WI38 Dox, WI38 Onc, HUVEC IR
47	*SNORA26*	no	no	no	no	yes	WI38 Dox, IMR90 Rep
48	*RNU2-36P*	no	no	no	no	no	
49	*SNORA50A (SNORA50)*	no	no	no	no	no	
50	*SNORA40*	no	no	no	no	no	
51	*SNORD1B*	no	no	no	no	no	

^1^ Genes with *p* < 0.0001 were analyzed, HAEC—human aortic endothelial cells, HUVEC—human umbilical vein endothelial cells, Dox—doxorubicin-induced senescence, IMR90—human diploid fibroblasts from fetal lung, IR—irradiation induced senescence, Rep—replicative exhaustion induced senescence, Onc—oncogene induced senescence, WI38—human diploid fibroblasts from fetal lung, no—there is no association between presented data and literature, yes—there is an association between presented data and literature

## References

[1] Moll F.L., Powell J.T., Fraedrich G., Verzini F., Haulon S., Waltham M., van Herwaarden J.A., Holt P.J., van Keulen J.W., Rantner B. (2011). European Society for Vascular Surgery. Management of abdominal aortic aneurysms clinical practice guidelines of the European society for vascular surgery. Eur. J. Vasc. Endovasc. Surg..

[2] Chaikof E.L., Dalman R.L., Eskandari M.K., Jackson B.M., Lee W.A., Mansour M.A., Mastracci T.M., Mell M., Murad M.H., Nguyen L.L. (2018). The Society for Vascular Surgery practice guidelines on the care of patients with an abdominal aortic aneurysm. J. Vasc. Surg..

[3] Nordon I.M., Hinchliffe R.J., Loftus I.M., Thompson M.M. (2011). Pathophysiology and epidemiology of abdominal aortic aneurysms. Nat. Rev. Cardiol..

[4] Salem M.K., Rayt H.S., Hussey G., Rafelt S., Nelson C.P., Sayers R.D., Naylor A.R., Nasim A. (2009). Should Asian men be included in abdominal aortic aneurysm screening programmes?. Eur. J. Vasc. Endovasc. Surg..

[5] GBD 2013 Mortality and Causes of Death Collaborators (2015). Global, regional, and national age-sex specific all-cause and cause-specific mortality for 240 causes of death, 1990–2013: A systematic analysis for the Global Burden of Disease Study 2013. Lancet.

[6] Choke E., Vijaynagar B., Thompson J., Nasim A., Bown M.J., Sayers R.D. (2012). Changing epidemiology of abdominal aortic aneurysms in England and Wales: Older and more benign?. Circulation.

[7] Sidloff D., Stather P., Dattani N., Bown M., Thompson J., Sayers R., Choke E. (2014). Aneurysm global epidemiology study: Public health measures can further reduce abdominal aortic aneurysm mortality. Circulation.

[8] Centers for Disease Control and Prevention, National Center for Health Statistics Multiple Cause of Death 1999-2018 on CDC WONDER Online Database, released in 2020. Data are from the Multiple Cause of Death Files, 1999-2018, as compiled from data provided by the 57 vital statistics jurisdictions through the Vital Statistics Cooperative Program. https://conifer.rhizome.org/DZal/cdc-wonder-aaa-mortality-in-usa/20200613060247/https://wonder.cdc.gov/controller/saved/D77/D86F192.

[9] Hernandez-Vaquero D., Silva J., Escalera A., Álvarez-Cabo R., Morales C., Díaz R., Avanzas P., Moris C., Pascual I. (2020). Life Expectancy after Surgery for Ascending Aortic Aneurysm. J. Clin. Med..

[10] Kent K.C., Zwolak R.M., Egorova N.N., Riles T.S., Manganaro A., Moskowitz A.J., Gelijns A.C., Greco G. (2010). Analysis of risk factors for abdominal aortic aneurysm in a cohort of more than 3 million individuals. J. Vasc. Surg..

[11] Ahmed R., Ghoorah K., Kunadian V. (2016). Abdominal Aortic Aneurysms and Risk Factors for Adverse Events. Cardiol. Rev..

[12] Rodríguez-Carrio J., Lindholt J.S., Canyelles M., Martínez-López D., Tondo M., Blanco-Colio L.M., Michel J.-B., Escolà-Gil J.C., Suárez A., Martín-Ventura J.L. (2020). IgG Anti-High Density Lipoprotein Antibodies Are Elevated in Abdominal Aortic Aneurysm and Associated with Lipid Profile and Clinical Features. J. Clin. Med..

[13] Golledge J., Kuivaniemi H. (2013). Genetics of abdominal aortic aneurysm. Curr. Opin. Cardiol..

[14] Bown M.J. (2014). Genomic insights into abdominal aortic aneurysms. Ann. R. Coll. Surg. Engl..

[15] Hong E.P., Kim B.J., Cho S.S., Yang J.S., Choi H.J., Kang S.H., Jeon J.P. (2019). Genomic Variations in Susceptibility to Intracranial Aneurysm in the Korean Population. J. Clin. Med..

[16] Hong E.P., Kim B.J., Jeon J.P. (2019). Genome-Wide Association between the 2q33.1 Locus and Intracranial Aneurysm Susceptibility: An Updated Meta-Analysis Including 18,019 Individuals. J. Clin. Med..

[17] Alamoudi A., Haque S., Srinivasan S., Mital D.P. (2015). Diagnostic efficacy value in terms of sensitivity and specificity of imaging modalities in detecting the abdominal aortic aneurysm: A systematic review. IJMEI.

[18] Hellenthal F.A., Pulinx B., Welten R.J., Teijink J.A., van Dieijen-Visser M.P., Wodzig W.K., Schurink G.W. (2012). Circulating biomarkers and abdominal aortic aneurysm size. J. Surg. Res..

[19] Moris D.N., Georgopoulos S.E. (2013). Circulating biomarkers for abdominal aortic aneurysm: What did we learn in the last decade?. Int. Angiol..

[20] Htun N.M., Peter K. (2014). Biomarkers for AAA: Encouraging steps but clinical relevance still to be delivered. Proteom. Clin. Appl..

[21] Kim D., Sung Y.M., Park J., Kim S., Kim J., Park J., Ha H., Bae J.Y., Kim S., Baek D. (2016). General rules for functional microRNA targeting. Nat. Genet..

[22] Guo H., Ingolia N.T., Weissman J.S., Bartel D.P. (2010). Mammalian microRNAs predominantly act to decrease target mRNA levels. Nature.

[23] Rupaimoole R., Slack F.J. (2017). MicroRNA therapeutics: Towards a new era for the management of cancer and other diseases. Nat. Rev. Drug Discov..

[24] Carvalho L.S. (2017). Can microRNAs improve prediction of abdominal aortic aneurysm growth?. Atherosclerosis.

[25] Raffort J., Lareyre F., Clement M., Mallat Z. (2016). Micro-RNAs in abdominal aortic aneurysms: Insights from animal models and relevance to human disease. Cardiovasc. Res..

[26] Iyer V., Rowbotham S., Biros E., Bingley J., Golledge J. (2017). A systematic review investigating the association of microRNAs with human abdominal aortic aneurysms. Atherosclerosis.

[27] Kumar S., Boon R.A., Maegdefessel L., Dimmeler S., Jo H. (2019). Role of Noncoding RNAs in the Pathogenesis of Abdominal Aortic Aneurysm. Circ. Res..

[28] Bogucka-Kocka A., Zalewski D.P., Ruszel K.P., Stępniewski A., Gałkowski D., Bogucki J., Komsta Ł., Kołodziej P., Zubilewicz T., Feldo M. (2019). Dysregulation of MicroRNA Regulatory Network in Lower Extremities Arterial Disease. Front. Genet..

[29] Zalewski D.P., Ruszel K.P., Stępniewski A., Gałkowski D., Bogucki J., Komsta Ł., Kołodziej P., Chmiel P., Zubilewicz T., Feldo M. (2020). Dysregulations of MicroRNA and Gene Expression in Chronic Venous Disease. J. Clin. Med..

[30] Centner V., Massart D.L., de Noord O.E., de Jong S., Vandeginste B.M., Sterna C. (1996). Elimination of uninformative variables for multivariate calibration. Anal. Chem..

[31] Love M.I., Huber W., Anders S. (2014). Moderated estimation of fold change and dispersion for RNA-seq data with DESeq2. Genome Biol..

[32] Mehmood T., Hovde K.H., Liland Snipen L., Sæbø S. (2012). A review of variable selection methods in Partial Least Squares Regression. Chemometr. Intell. Lab. Syst..

[33] Chen H., Boutros P.C. (2011). VennDiagram: A package for the generation of highly-customizable Venn and Euler diagrams in R. BMC Bioinform..

[34] Robin X., Turck N., Hainard A., Tiberti N., Lisacek F., Sanchez J.C., Müller M. (2011). pROC: An open-source package for R and S+ to analyze and compare ROC curves. BMC Bioinform..

[35] Finotello F., Mayer C., Plattner C., Laschober G., Rieder D., Hackl H., Krogsdam A., Loncova Z., Posch W., Wilflingseder D. (2019). Molecular and pharmacological modulators of the tumor immune contexture revealed by deconvolution of RNA-seq data. Genome Med..

[36] Becht E., Giraldo N.A., Lacroix L., Buttard B., Elarouci N., Petitprez F., Selves J., Laurent-Puig P., Sautès-Fridman C., Fridman W.H. (2016). Estimating the population abundance of tissue-infiltrating immune and stromal cell populations using gene expression. Genome Biol..

[37] Sturm G., Finotello F., Petitprez F., Zhang J.D., Baumbach J., Fridman W.H., List M., Aneichyk T. (2019). Comprehensive evaluation of transcriptome-based cell-type quantification methods for immuno-oncology. Bioinformatics.

[38] Ru Y., Kechris K.J., Tabakoff B., Hoffman P., Radcliffe R.A., Bowler R., Mahaffey S., Rossi S., Calin G.A., Bemis L. (2014). The multiMiR R package and database: Integration of microRNA–target interactions along with their disease and drug associations. Nucleic Acids Res..

[39] Shannon P., Markiel A., Ozier O., Baliga N.S., Wang J.T., Ramage D., Amin N., Schwikowski B., Ideker T. (2003). Cytoscape: A software environment for integrated models of biomolecular interaction networks. Genome Res..

[40] Huang D.W., Sherman B.T., Lempicki R.A. (2009). Systematic and integrative analysis of large gene lists using DAVID Bioinformatics Resources. Nat. Protoc..

[41] Huang D.W., Sherman B.T., Lempicki R.A. (2009). Bioinformatics enrichment tools: Paths toward the comprehensive functional analysis of large gene lists. Nucleic Acids Res..

[42] Araujo N.N.F., Lin-Wang H.T., Germano J.F., Farsky P.S., Feldman A., Rossi F.H., Izukawa N.M., Higuchi M.L., Savioli Neto F., Hirata M.H. (2019). Dysregulation of microRNAs and target genes networks in human abdominal aortic aneurysm tissues. PLoS ONE..

[43] Wanhainen A., Mani K., Vorkapic E., De Basso R., Björck M., Länne T., Wågsäter D. (2017). Screening of circulating microRNA biomarkers for prevalence of abdominal aortic aneurysm and aneurysm growth. Atherosclerosis.

[44] Stather P.W., Sylvius N., Sidloff D.A., Dattani N., Verissimo A., Wild J.B., Butt H.Z., Choke E., Sayers R.D., Bown M.J. (2015). Identification of microRNAs associated with abdominal aortic aneurysms and peripheral arterial disease. Br. J. Surg..

[45] Zhang W., Shang T., Huang C., Yu T., Liu C., Qiao T., Huang D., Liu Z., Liu C. (2015). Plasma microRNAs serve as potential biomarkers for abdominal aortic aneurysm. Clin. Biochem..

[46] Pahl M.C., Derr K., Gäbel G., Hinterseher I., Elmore J.R., Schworer C.M., Peeler T.C., Franklin D.P., Gray J.L., Carey D.J. (2012). MicroRNA expression signature in human abdominal aortic aneurysms. BMC Med. Genom..

[47] Kin K., Miyagawa S., Fukushima S., Shirakawa Y., Torikai K., Shimamura K., Daimon T., Kawahara Y., Kuratani T., Sawa Y. (2012). Tissue- and plasma-specific MicroRNA signatures for atherosclerotic abdominal aortic aneurysm. J. Am. Heart Assoc..

[48] Busch A., Busch M., Scholz C.J., Kellersmann R., Otto C., Chernogubova E., Maegdefessel L., Zernecke A., Lorenz U. (2016). Aneurysm miRNA Signature Differs, Depending on Disease Localization and Morphology. Int. J. Mol. Sci..

[49] Canfrán-Duque A., Rotllan N., Zhang X., Fernández-Fuertes M., Ramírez-Hidalgo C., Araldi E., Daimiel L., Busto R., Fernández-Hernando C., Suárez Y. (2017). Macrophage deficiency of miR-21 promotes apoptosis, plaque necrosis, and vascular inflammation during atherogenesis. EMBO Mol. Med..

[50] Maegdefessel L., Azuma J., Toh R., Deng A., Merk D.R., Raiesdana A., Leeper N.J., Raaz U., Schoelmerich A.M., McConnell M.V. (2012). MicroRNA-21 blocks abdominal aortic aneurysm development and nicotine-augmented expansion. Sci. Transl. Med..

[51] Liu X., Cheng Y., Yang J., Krall T.J., Huo Y., Zhang C. (2010). An essential role of PDCD4 in vascular smooth muscle cell apoptosis and proliferation: Implications for vascular disease. Am. J. Physiol. Cell. Physiol..

[52] Cheng Y., Liu X., Zhang S., Lin Y., Yang J., Zhang C. (2009). MicroRNA-21 protects against the H_2_O_2_-induced injury on cardiac myocytes via its target gene PDCD4. J. Mol. Cell. Cardiol..

[53] Gao Y., Li H., Zhou Y., Lv H., Chen Y. (2019). PDCD4 expression in coronary atherosclerosis rat models and its mechanism. Exp. Ther. Med..

[54] Lenk G.M., Tromp G., Weinsheimer S., Gatalica Z., Berguer R., Kuivaniemi H. (2007). Whole genome expression profiling reveals a significant role for immune function in human abdominal aortic aneurysms. BMC Genom..

[55] Choke E., Cockerill G.W., Laing K., Dawson J., Wilson W.R., Loftus I.M., Thompson M.M. (2009). Whole genome-expression profiling reveals a role for immune and inflammatory response in abdominal aortic aneurysm rupture. Eur. J. Vasc. Endovas. Surg..

[56] Giusti B., Rossi L., Lapini I., Magi A., Pratesi G., Lavitrano M., Biasi G.M., Pulli R., Pratesi C., Abbate R. (2009). Gene expression profiling of peripheral blood in patients with abdominal aortic aneurysm. Eur. J. Vasc. Endovasc. Surg..

[57] Butt H.Z., Sylvius N., Salem M.K., Wild J.B., Dattani N., Sayers R.D., Bown M.J. (2016). Microarray-based Gene Expression Profiling of Abdominal Aortic Aneurysm. Eur. J. Vasc. Endovasc. Surg..

[58] Sirisaengtaksin N., Gireud M., Yan Q., Kubota Y., Meza D., Waymire J.C., Zage P.E., Bean A.J. (2014). UBE4B protein couples ubiquitination and sorting machineries to enable epidermal growth factor receptor (EGFR) degradation. J. Biol. Chem..

[59] Tomas A., Futter C.E., Eden E.R. (2014). EGF receptor trafficking: Consequences for signaling and cancer. Trends Cell Biol..

[60] Wu C.T., Lin W.Y., Chang Y.H., Lin P.Y., Chen W.C., Chen M.F. (2015). DNMT1-dependent suppression of microRNA424 regulates tumor progression in human bladder cancer. Oncotarget.

[61] Deegan D.F., Karbalaei R., Madzo J., Kulathinal R.J., Engel N. (2019). The developmental origins of sex-biased expression in cardiac development. Biol. Sex Differ..

[62] Naqvi S., Godfrey A.K., Hughes J.F., Goodheart M.L., Mitchell R.N., Page D.C. (2019). Conservation, acquisition, and functional impact of sex-biased gene expression in mammals. Science.

[63] Cui C., Yang W., Shi J., Zhou Y., Yang J., Cui Q., Zhou Y. (2018). Identification and Analysis of Human Sex-biased MicroRNAs. Genom. Proteom. Bioinform..

[64] Calabria E., Mazza E.M., Dyar K.A., Pogliaghi S., Bruseghini P., Morandi C., Salvagno G.L., Gelati M., Guidi G.C., Bicciato S. (2016). Aging: A portrait from gene expression profile in blood cells. Aging.

[65] Casella G., Munk R., Kim K.M., Piao Y., De S., Abdelmohsen K., Gorospe M. (2019). Transcriptome signature of cellular senescence. Nucleic Acids Res..

[66] Kaur G., Begum R., Thota S., Batra S. (2019). A systematic review of smoking-related epigenetic alterations. Arch. Toxicol..

[67] Maegdefessel L., Spin J.M., Raaz U., Eken S.M., Toh R., Azuma J., Adam M., Nakagami F., Heymann H.M., Chernogubova E. (2014). miR-24 limits aortic vascular inflammation and murine abdominal aneurysm development. Nat. Commun..

[68] Rotllan N., Fernández-Hernando C. (2012). MicroRNA Regulation of Cholesterol Metabolism. Cholesterol.

[69] Oneyama C., Kito Y., Asai R., Ikeda J., Yoshida T., Okuzaki D., Kokuda R., Kakumoto K., Takayama K., Inoue S. (2013). MiR-424/503-mediated Rictor upregulation promotes tumor progression. PLoS ONE.

[70] Dai F., Mei L., Meng S., Ma Z., Guo W., Zhou J., Zhang J. (2017). The global expression profiling in esophageal squamous cell carcinoma. Genomics.

[71] Zhu D.Y., Li X.N., Qi Y., Liu D.L., Yang Y., Zhao J., Zhang C.Y., Wu K., Zhao S. (2016). MiR-454 promotes the progression of human non-small cell lung cancer and directly targets PTEN. Biomed. Pharmacother..

[72] Ren L., Chen H., Song J., Chen X., Lin C., Zhang X., Hou N., Pan J., Zhou Z., Wang L. (2019). MiR-454-3p-Mediated Wnt/β-catenin Signaling Antagonists Suppression Promotes Breast Cancer Metastasis. Theranostics.

[73] Jiang L., Zhao Z., Zheng L., Xue L., Zhan Q., Song Y. (2017). Downregulation of miR-503 Promotes ESCC Cell Proliferation, Migration, and Invasion by Targeting Cyclin D1. Genom. Proteom. Bioinform..

[74] Polioudakis D., Abell N.S., Iyer V.R. (2015). miR-503 represses human cell proliferation and directly targets the oncogene DDHD2 by non-canonical target pairing. BMC Genom..

[75] Lai C.Y., Lee S.Y., Scarr E., Yu Y.H., Lin Y.T., Liu C.M., Hwang T.J., Hsieh M.H., Liu C.C., Chien Y.L. (2016). Aberrant expression of microRNAs as biomarker for schizophrenia: From acute state to partial remission, and from peripheral blood to cortical tissue. Transl. Psychiatry.

[76] Boileau A., Lino Cardenas C.L., Courtois A., Zhang L., Rodosthenous R.S., Das S., Sakalihasan N., Michel J.B., Lindsay M.E., Devaux Y. (2019). MiR-574-5p: A Circulating Marker of Thoracic Aortic Aneurysm. Int. J. Mol. Sci..

[77] Yao P., Wu J., Lindner D., Fox P.L. (2017). Interplay between miR-574-3p and hnRNP L regulates VEGFA mRNA translation and tumorigenesis. Nucleic Acids Res..

[78] Lai Z., Lin P., Weng X., Su J., Chen Y., He Y., Wu G., Wang J., Yu Y., Zhang L. (2018). MicroRNA-574-5p promotes cell growth of vascular smooth muscle cells in the progression of coronary artery disease. Biomed. Pharmacother..

[79] Cui F., Zhou Q., Xiao K., Ma S. (2020). The MicroRNA hsa-let-7g Promotes Proliferation and Inhibits Apoptosis in Lung Cancer by Targeting HOXB1. YMJ.

[80] Zhou J.L., Deng S., Fang H.S., Yu G., Peng H. (2019). Hsa-let-7g promotes osteosarcoma by reducing HOXB1 to activate NF-kB pathway. Biomed. Pharmacother..

[81] Wang X., Zhang Y., Jiang B.H., Zhang Q., Zhou R.P., Zhang L., Wang C. (2017). Study on the role of Hsa-miR-31-5p in hypertrophic scar formation and the mechanism. Exp. Cell Res..

[82] Cho J.H., Dimri M., Dimri G.P. (2015). MicroRNA-31 is a transcriptional target of histone deacetylase inhibitors and a regulator of cellular senescence. J. Biol. Chem..

[83] Yang S.Y., Wang Y.Q., Gao H.M., Wang B., He Q. (2016). The clinical value of circulating miR-99a in plasma of patients with acute myocardial infarction. Eur. Rev. Med. Pharmacol. Sci..

[84] Jin Y., Tymen S.D., Chen D., Fang Z.J., Zhao Y., Dragas D., Dai Y., Marucha P.T., Zhou X. (2013). MicroRNA-99 family targets AKT/mTOR signaling pathway in dermal wound healing. PLoS ONE.

[85] Bo L., Wei B., Wang Z., Kong D., Gao Z., Miao Z. (2017). Screening of Critical Genes and MicroRNAs in Blood Samples of Patients with Ruptured Intracranial Aneurysms by Bioinformatic Analysis of Gene Expression Data. Med. Sci. Monit..

[86] Han Y., Liu Y., Zhang H., Wang T., Diao R., Jiang Z., Gui Y., Cai Z. (2013). Hsa-miR-125b suppresses bladder cancer development by down-regulating oncogene SIRT7 and oncogenic long non-coding RNA MALAT1. FEBS Lett..

[87] Liu Z.Z., Tian Y.F., Wu H., Ouyang S.Y., Kuang W.L. (2020). LncRNA H19 promotes glioma angiogenesis through miR-138/HIF-1α/VEGF axis. Neoplasma.

[88] Wang C., Sun X., Qiu Z., Chen A. (2019). MiR-138-5p exacerbates hypoxia/reperfusion-induced heart injury through the inactivation of SIRT1-PGC-1α. Inflamm. Res..

[89] Chen X., Xu X., Pan B., Zeng K., Xu M., Liu X., He B., Pan Y., Sun H., Wang S. (2018). miR-150-5p suppresses tumor progression by targeting VEGFA in colorectal cancer. Aging.

[90] Li H., Zhang P., Li F., Yuan G., Wang X., Zhang A., Li F. (2019). Plasma miR-22-5p, miR-132-5p, and miR-150-3p Are Associated with Acute Myocardial Infarction. BioMed Res. Int..

[91] Wang S., Tang D., Wang W., Yang Y., Wu X., Wang L., Wang D. (2019). circLMTK2 acts as a sponge of miR-150-5p and promotes proliferation and metastasis in gastric cancer. Mol. Cancer..

[92] Wang Y.L., Chen C.M., Wang X.M., Wang L. (2016). Effects of miR-339-5p on invasion and prognosis of hepatocellular carcinoma. Clin. Res. Hepatol. Gastroenterol..

[93] Seleem M., Shabayek M., Ewida H.A. (2019). MicroRNAs 342 and 450 together with NOX-4 activity and their association with coronary artery disease in diabetes. Diabetes Metab. Res. Rev..

[94] Qing Y., Huang M., Cao Y., Du T., Song K. (2019). Effects of miRNA-342-3p in modulating Hedgehog signaling pathway of human umbilical cord mesenchymal stem cells by down-regulating Sufu. Oral Dis..

[95] Lu C., Jia S., Zhao S., Shao X. (2019). MiR-342 regulates cell proliferation and apoptosis in hepatocellular carcinoma through Wnt/β-catenin signaling pathway. Cancer Biomark..

[96] Lago T.S., Silva J.A., Lago E.L., Carvalho E.M., Zanette D.L., Castellucci L.C. (2018). The miRNA 361-3p, a Regulator of GZMB and TNF Is Associated with Therapeutic Failure and Longer Time Healing of Cutaneous Leishmaniasis Caused by L. (viannia) braziliensis. Front. Immunol..

[97] Hayakawa K., Kawasaki M., Hirai T., Yoshida Y., Tsushima H., Fujishiro M., Ikeda K., Morimoto S., Takamori K., Sekigawa I. (2019). MicroRNA-766-3p Contributes to Anti-Inflammatory Responses through the Indirect Inhibition of NF-κB Signaling. Int. J. Mol Sci..

[98] Freedman J.E., Ercan B., Morin K.M., Liu C.T., Tamer L., Ayaz L., Kanadasi M., Cicek D., Seyhan A.I., Akilli R.E. (2012). The distribution of circulating microRNA and their relation to coronary disease. F1000Research.

[99] Han C., Song Y., Lian C. (2018). MiR-769 Inhibits Colorectal Cancer Cell Proliferation and Invasion by Targeting HEY1. Med. Sci. Monit..

[100] Ma G., Zhu J., Liu F., Yang Y. (2019). Long Noncoding RNA LINC00460 Promotes the Gefitinib Resistance of Nonsmall Cell Lung Cancer Through Epidermal Growth Factor Receptor by Sponging miR-769-5p. DNA Cell Biol..

[101] Yuan R.B., Zhang S.H., He Y., Zhang X.Y., Zhang Y.B. (2018). MiR-874-3p is an independent prognostic factor and functions as an anti-oncomir in esophageal squamous cell carcinoma via targeting STAT3. Eur. Rev. Med. Pharmacol. Sci..

[102] Bakhshmand E.A., Mohammad Soltani B.M., Fasihi A., Mowla S.J. (2018). Hsa-miR-5582-3P regulatory effect on TGFβ signaling through targeting of TGFβ-R1, TGFβ-R2, SMAD3, and SMAD4 transcripts. J. Cell Biochem..

[103] Schipper H.M., Maes O.C., Chertkow H.M., Wang E. (2007). MicroRNA expression in Alzheimer blood mononuclear cells. Gene Regul. Syst. Biol..

[104] Delavar M., Baghi M., Safaeinejad Z., Kiani-Esfahani A., Ghaedi K., Nasr-Esfahani M.H. (2018). Differential expression of miR-34a, miR-141, and miR-9 in MPP+-treated differentiated PC12 cells as a model of Parkinson’s disease. Gene.

[105] Ameling S., Kacprowski T., Chilukoti R.K., Malsch C., Liebscher V., Suhre K., Pietzner M., Friedrich N., Homuth G., Hammer E. (2015). Associations of circulating plasma microRNAs with age, body mass index and sex in a population-based study. BMC Med. Genom.

[106] Dhahbi J.M., Atamna H., Boffelli D., Magis W., Spindler S.R., Martin D.I. (2011). Deep sequencing reveals novel microRNAs and regulation of microRNA expression during cell senescence. PLoS ONE.

[107] Suh N. (2018). MicroRNA controls of cellular senescence. BMB Rep..

[108] Owczarz M., Budzinska M., Domaszewska-Szostek A., Borkowska J., Polosak J., Gewartowska M., Slusarczyk P., Puzianowska-Kuznicka M. (2017). miR-34a and miR-9 are overexpressed and SIRT genes are downregulated in peripheral blood mononuclear cells of aging humans. Exp. Biol. Med..

[109] Shi B., Gao H., Zhang T., Cui Q. (2016). Analysis of plasma microRNA expression profiles revealed different cancer susceptibility in healthy young adult smokers and middle-aged smokers. Oncotarget.

[110] Budzinska M., Owczarz M., Pawlik-Pachucka E., Roszkowska-Gancarz M., Slusarczyk P., Puzianowska-Kuznicka M. (2016). miR-96, miR-145 and miR-9 expression increases, and IGF-1R and FOXO1 expression decreases in peripheral blood mononuclear cells of aging humans. BMC Geriatr..

[111] Wang G., Wang R., Strulovici-Barel Y., Salit J., Staudt M.R., Ahmed J., Tilley A.E., Yee-Levin J., Hollmann C., Harvey B.G. (2015). Persistence of smoking-induced dysregulation of miRNA expression in the small airway epithelium despite smoking cessation. PLoS ONE.

[112] Su M.W., Yu S.L., Lin W.C., Tsai C.H., Chen P.H., Lee Y.L. (2016). Smoking-related microRNAs and mRNAs in human peripheral blood mononuclear cells. Toxicol. Appl. Pharmacol..

[113] Berrandou T., Mulot C., Cordina-Duverger E., Arveux P., Laurent-Puig P., Truong T., Guénel P. (2019). Association of breast cancer risk with polymorphisms in genes involved in the metabolism of xenobiotics and interaction with tobacco smoking: A gene-set analysis. Int. J. Cancer.

[114] Joehanes R., Just A.C., Marioni R.E., Pilling L.C., Reynolds L.M., Mandaviya P.R., Guan W., Xu T., Elks C.E., Aslibekyan S. (2016). Epigenetic Signatures of Cigarette Smoking. Circ. Cardiovasc. Genet..

[115] Obeidat M., Zhou G., Li X., Hansel N.N., Rafaels N., Mathias R., Ruczinski I., Beaty T.H., Barnes K.C., Paré P.D. (2018). The genetics of smoking in individuals with chronic obstructive pulmonary disease. Respir. Res..

[116] Parker M.M., Chase R.P., Lamb A., Reyes A., Saferali A., Yun J.H., Himes B.E., Silverman E.K., Hersh C.P., Castaldi P.J. (2017). RNA sequencing identifies novel non-coding RNA and exon-specific effects associated with cigarette smoking. BMC Med. Genom.

[117] Sridhar S., Schembri F., Zeskind J., Shah V., Gustafson A.M., Steiling K., Liu G., Dumas Y.M., Zhang X., Brody J.S. (2008). Smoking-induced gene expression changes in the bronchial airway are reflected in nasal and buccal epithelium. BMC Genom.

[118] Su D., Wang X., Campbell M.R., Porter D.K., Pittman G.S., Bennett B.D., Wan M., Englert N.A., Crowl C.L., Gimple R.N. (2016). Distinct Epigenetic Effects of Tobacco Smoking in Whole Blood and among Leukocyte Subtypes. PLoS ONE.

[119] Huan T., Joehanes R., Schurmann C., Schramm K., Pilling L.C., Peters M.J., Mägi R., DeMeo D., O’Connor G.T., Ferrucci L. (2016). A whole-blood transcriptome meta-analysis identifies gene expression signatures of cigarette smoking. Hum. Mol. Genet..

[120] Arimilli S., Madahian B., Chen P., Marano K., Prasad G.L. (2017). Gene expression profiles associated with cigarette smoking and moist snuff consumption. BMC Genom..

[121] Whiting C.C., Siebert J., Newman A.M., Du H.W., Alizadeh A.A., Goronzy J., Weyand C.M., Krishnan E., Fathman C.G., Maecker H.T. (2015). Large-Scale and Comprehensive Immune Profiling and Functional Analysis of Normal Human Aging. PLoS ONE.

[122] de Magalhães J.P., Curado J., Church G.M. (2009). Meta-analysis of age-related gene expression profiles identifies common signatures of aging. Bioinformatics.

[123] Peters M.J., Joehanes R., Pilling L.C., Schurmann C., Conneely K.N., Powell J., Reinmaa E., Sutphin G.L., Zhernakova A., Schramm K. (2015). The transcriptional landscape of age in human peripheral blood. Nat. Commun..

