# Supplementary material for: Dysregulation of microRNA Modulatory Network in Abdominal Aortic Aneurysm
Source: J Clin Med. 2020 Jun 24;9(6):1974. doi: 10.3390/jcm9061974 (PMC7355415; doi:10.3390/jcm9061974)
Supplement: Supplementary file 1 [file jcm-09-01974-s001.pdf]

SUPPLEMENTARY MATERIAL

Supplementary Figures

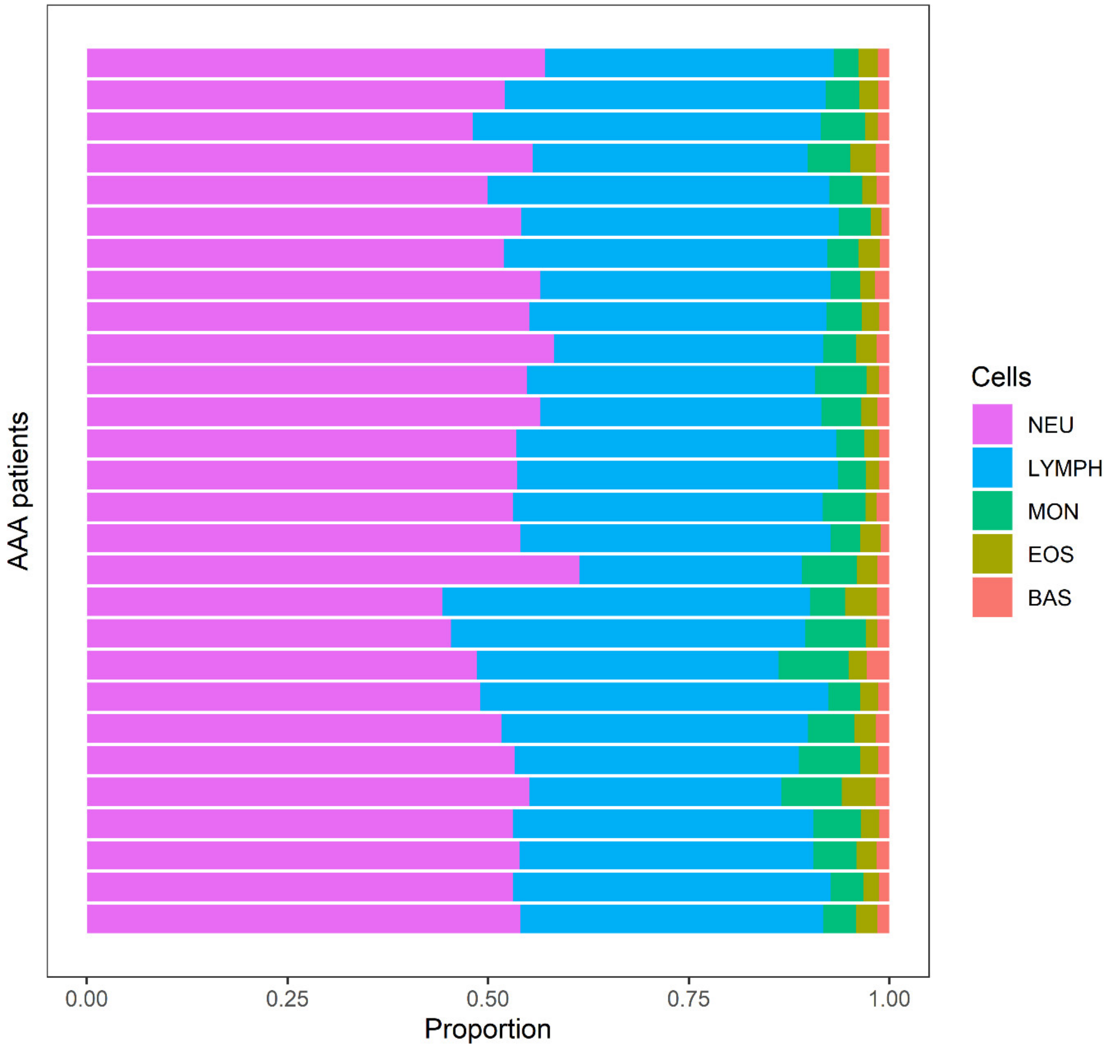

Figure S1. Proportions in white blood cells subpopulations in AAA patients resulted from blood morphology analysis. NEU – neutrophils, LYMPH – lymphocytes, MON – monocytes, EOS – eosinophils. BAS – basophils.

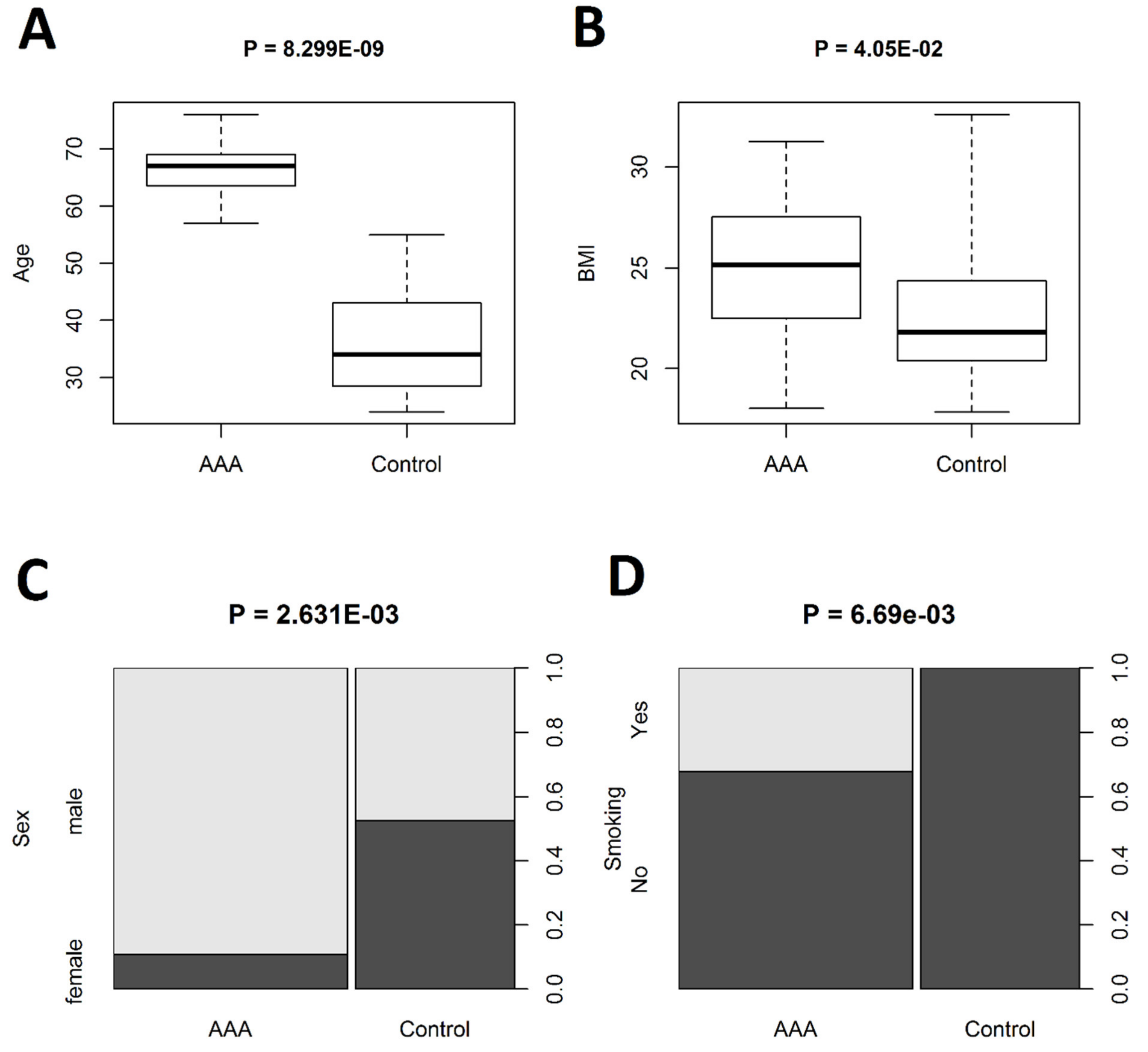

Figure S2. Evaluation of differences between AAA group (n = 28) and the control group (n = 19) for age (A), BMI (B), sex (C) and smoking (D). Statistical significance (*p* values) were calculated using two-sided Mann–Whitney *U* test (for age and BMI) and a two-sided Fisher's exact test (for sex and smoking). Distributions of age and BMI were presented on boxplots (A) and (B), respectively, with whiskers defining region between minimum and maximum values, boxes covering values between 25% and 75% quantile and horizontal lines inside boxes marking median value. Distribution of sex (black – female, gray – male) and smoking (black – no-smokers, grey – smokers) were presented on spine plots (C) and (D), respectively.

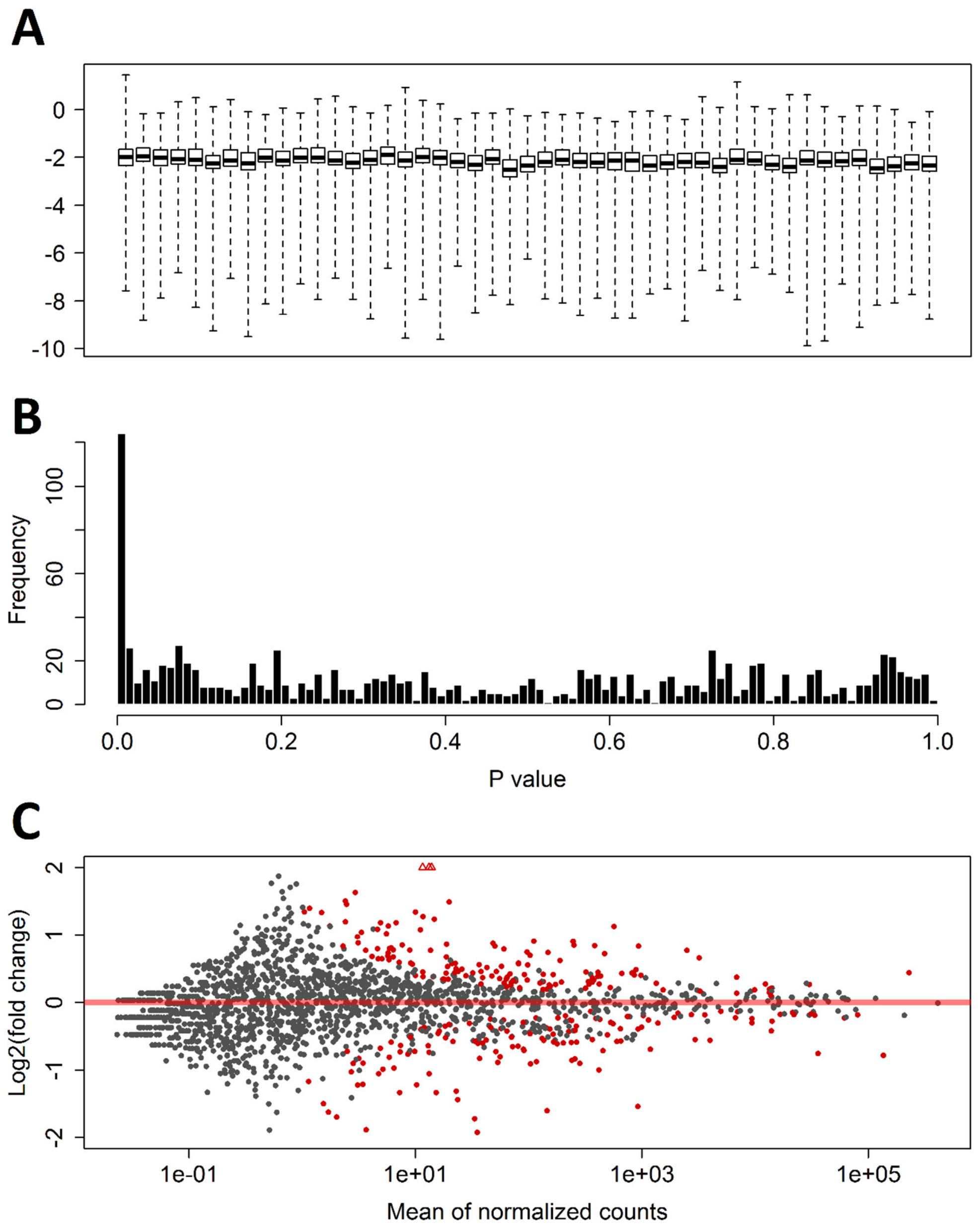

Figure S3. Quality control of data obtained from sequencing of small RNA libraries and results of differential expression analysis performed by DESeq2 package in 28 AAA individuals and 19 healthy controls. (A) Boxplot presenting Cook's distances of miRNAs across samples. Whiskers define range between minimum and maximum value of Cook's distance, boxes range between 25% and 75% quartile, horizontal lines inside boxes mark median value. (B) Histogram presenting distribution of DESeq2 *p* values. (C) MA plot showing relation between log2 of fold changes of differentially expressed miRNAs and averages of normalized counts. MiRNAs with *p* value < 0.1 were marked as red points.

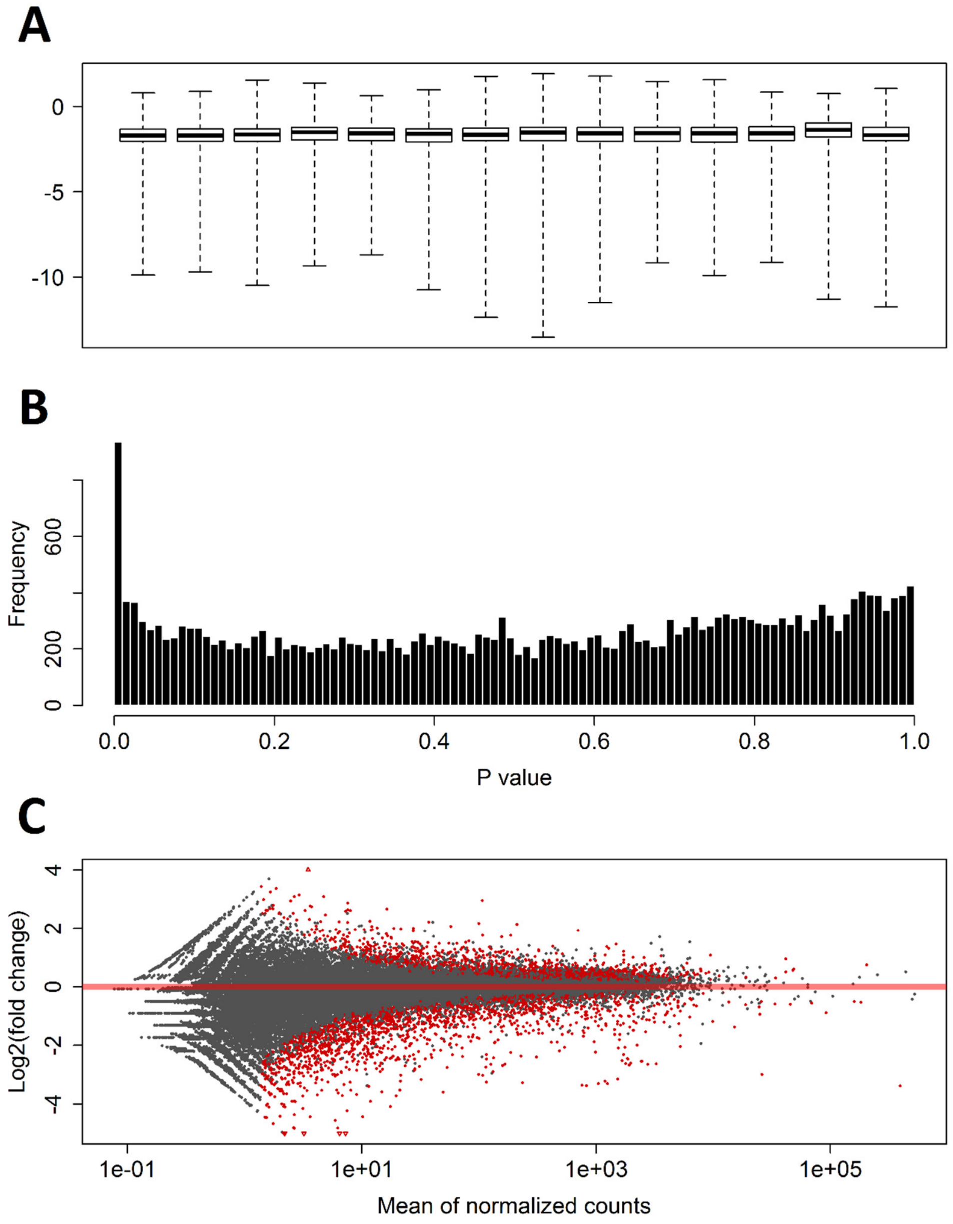

Figure S4. Quality control of data obtained from sequencing of transcriptome libraries and results of differential expression analysis performed by DESeq2 package between group of 7 AAA individuals and a group of 7 healthy controls. (A) Boxplot presenting Cook's distances of miRNAs across samples. Whiskers define range between minimum and maximum value of Cook's distance, boxes range between 25% and 75% quartile, horizontal lines inside boxes mark median value. (B) Histogram presenting distribution of DESeq2 *p* values. (C) MA plot showing relation between log2 of fold changes of differentially expressed miRNAs and averages of normalized counts. MiRNAs with *p* value < 0.1 were marked as red points.

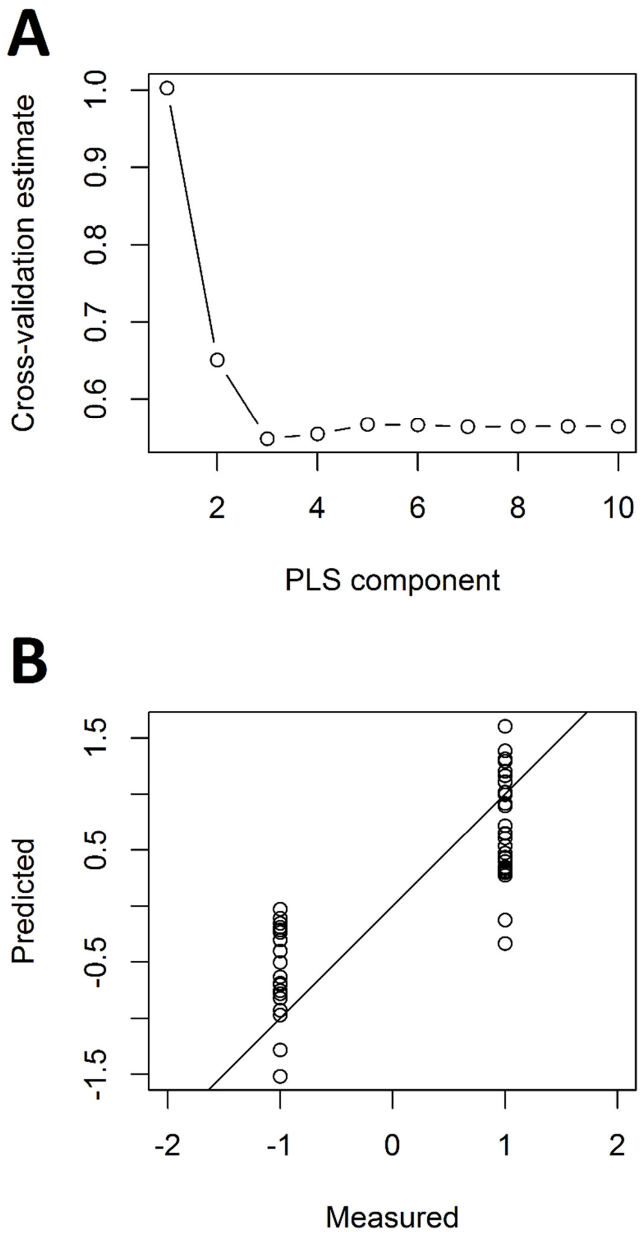

Figure S5. UVE-PLS differential expression analysis of miRNA expression data of 28 AAA patients compared to 19 healthy controls. Plots present the arrangement of prediction error and PLS components (A) and cross-validated predictions versus measured values (B).

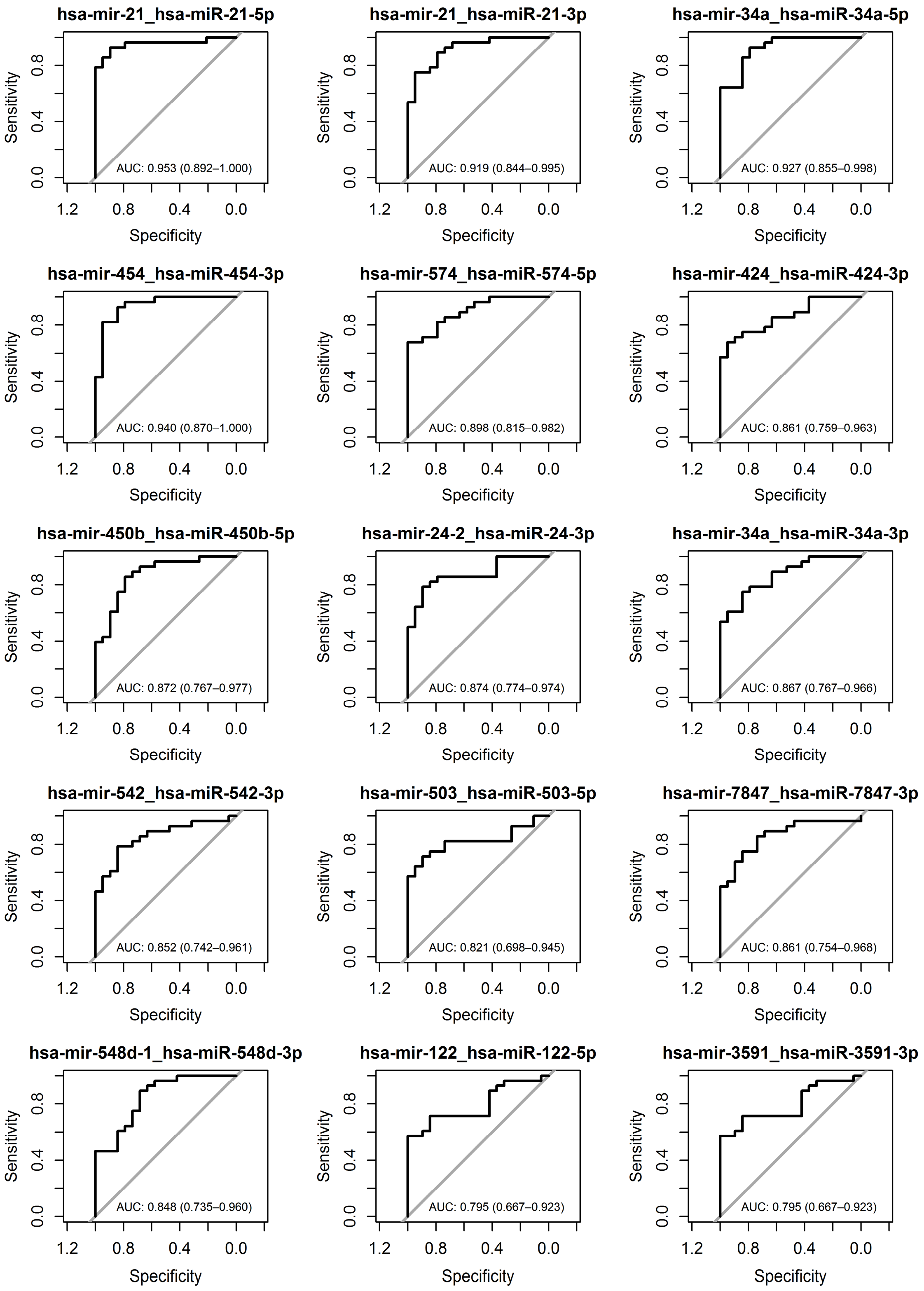

Figure S6. Results of receiver operating characteristics (ROC) analysis performed for selected 33 miRNA transcripts differentially expressed in AAA. Values of area under curves (AUC) with 95% confidence interval (in brackets) were included in each plot.

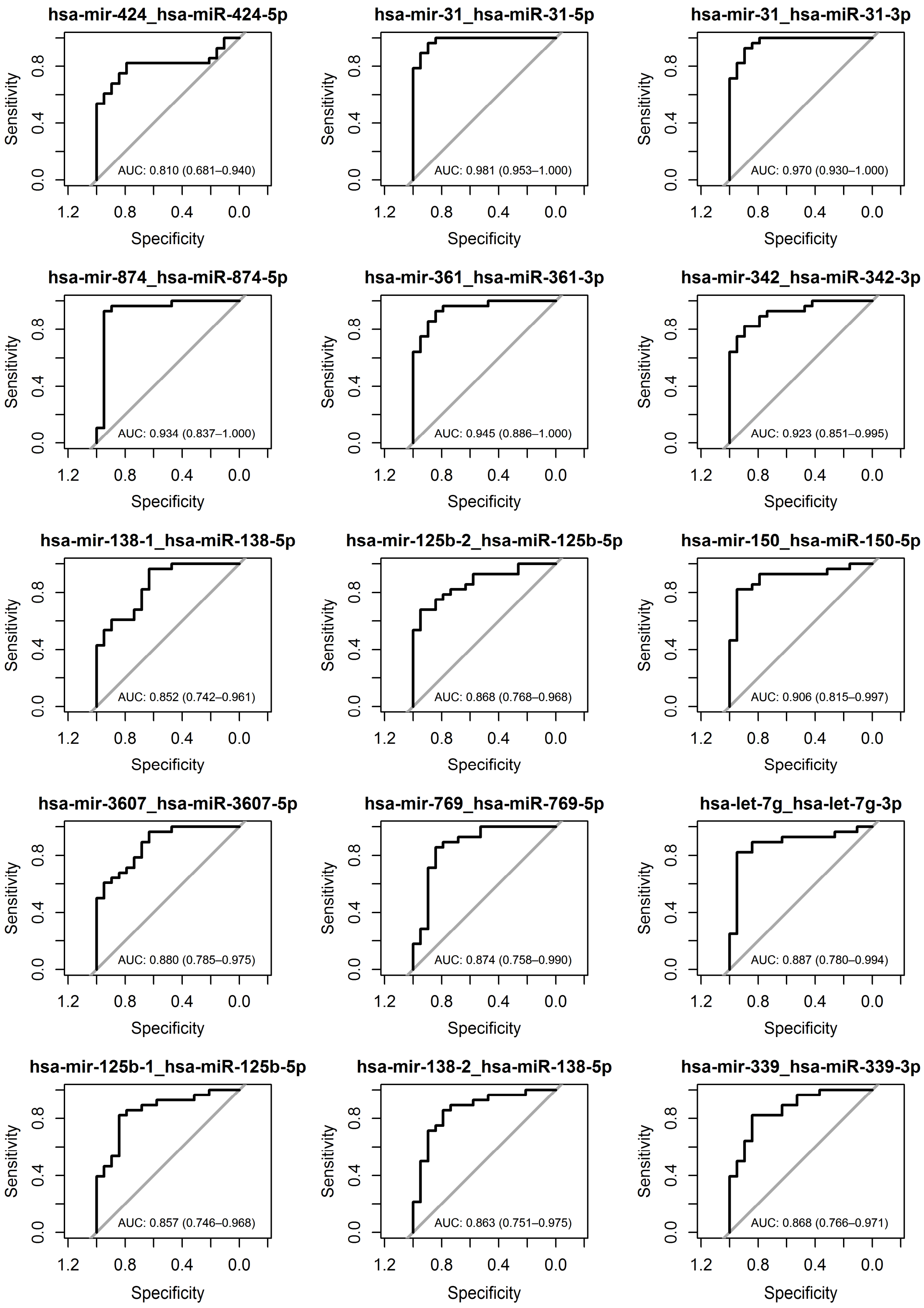

Figure S6. (continued).

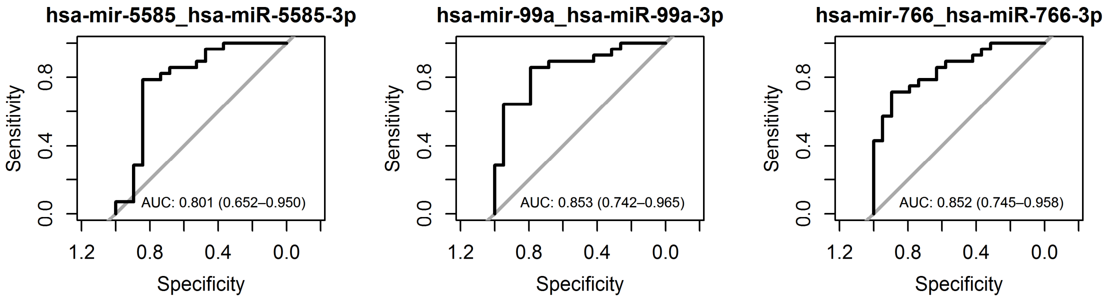

Figure S6. (continued).

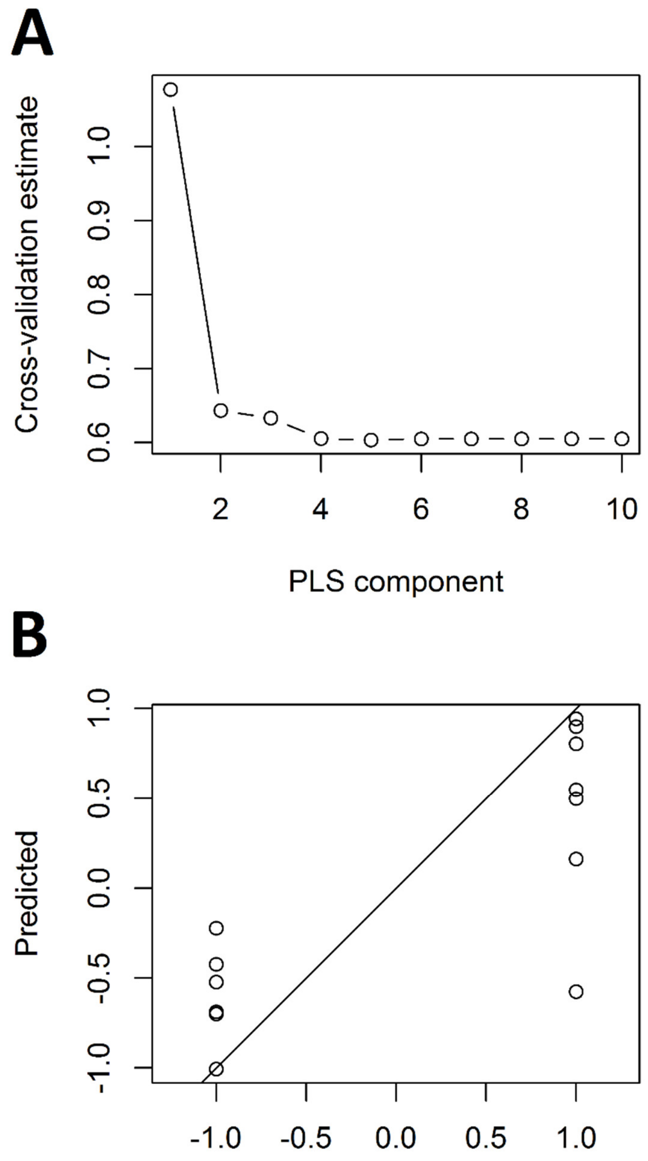

Figure S7. UVE-PLS differential expression analysis of transcriptomic expression data of 7 AAA individuals compared to 7 healthy controls. Plots present the arrangement of prediction error and PLS components (A) and cross-validated predictions versus measured values (B).

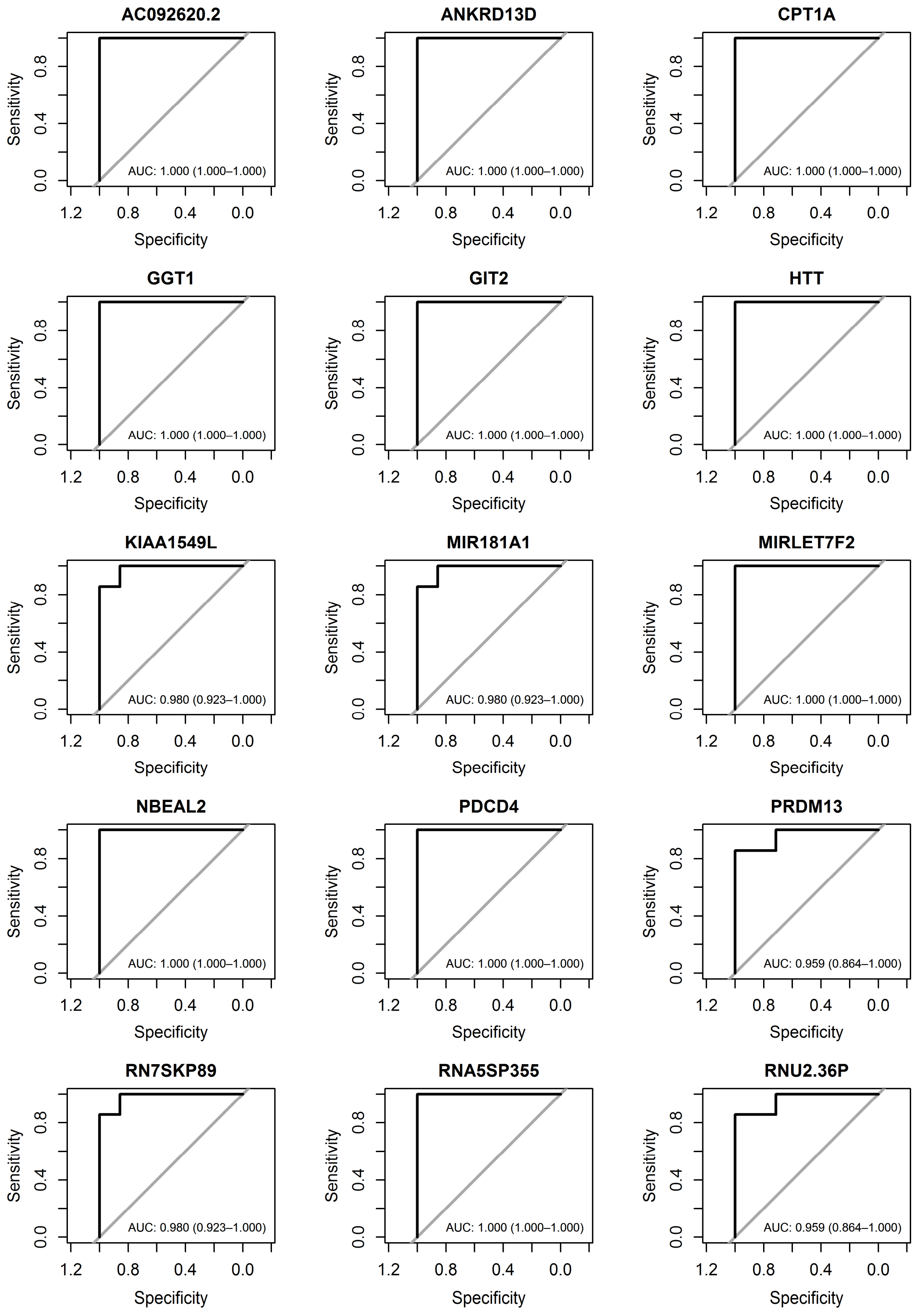

Figure S8. Results of receiver operating characteristics (ROC) analysis of 51 genes selected as signatures of AAA. Values of area under curves (AUC) with 95% confidence interval (in brackets) were included in each plot.

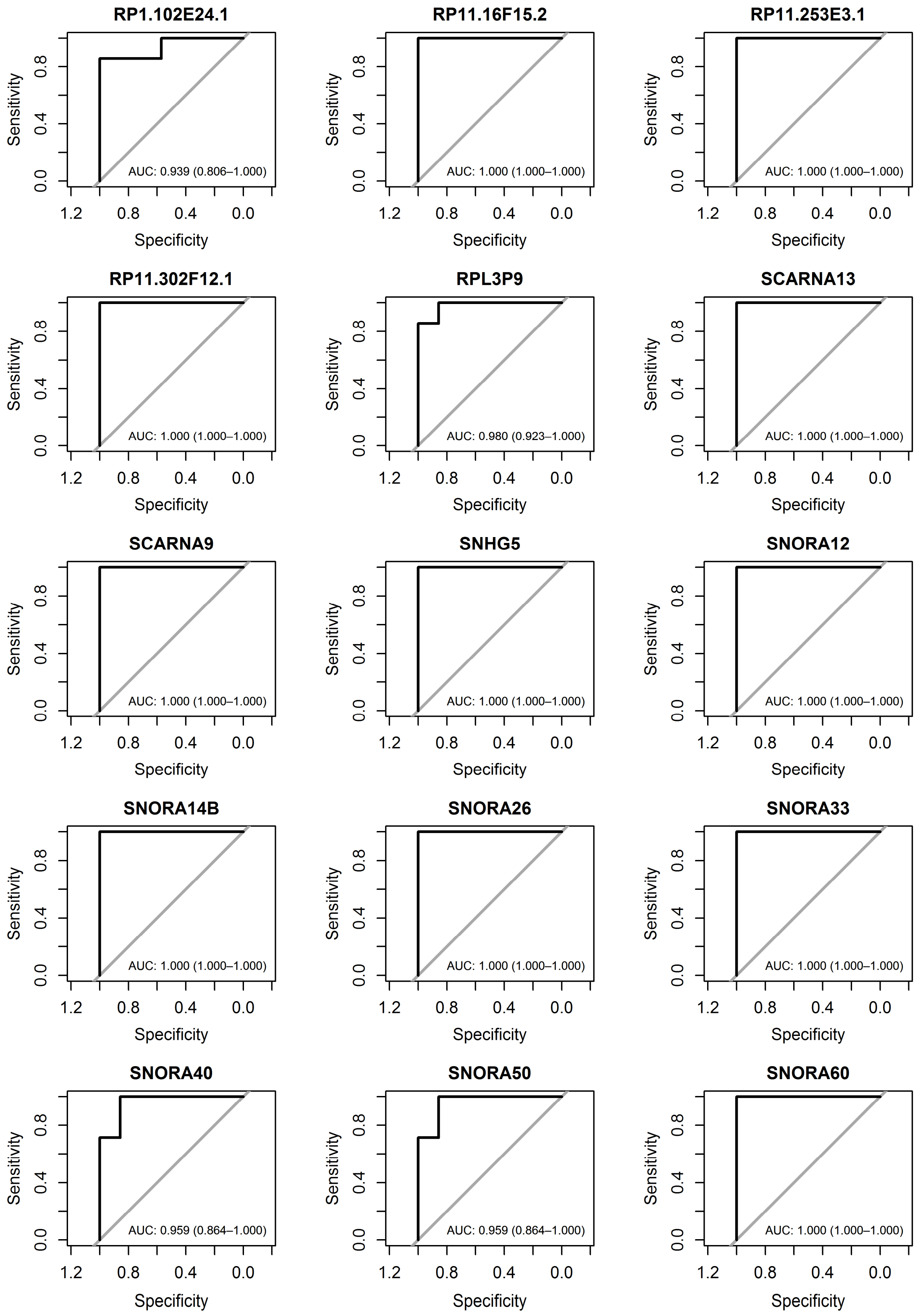

Figure S8. (continued).

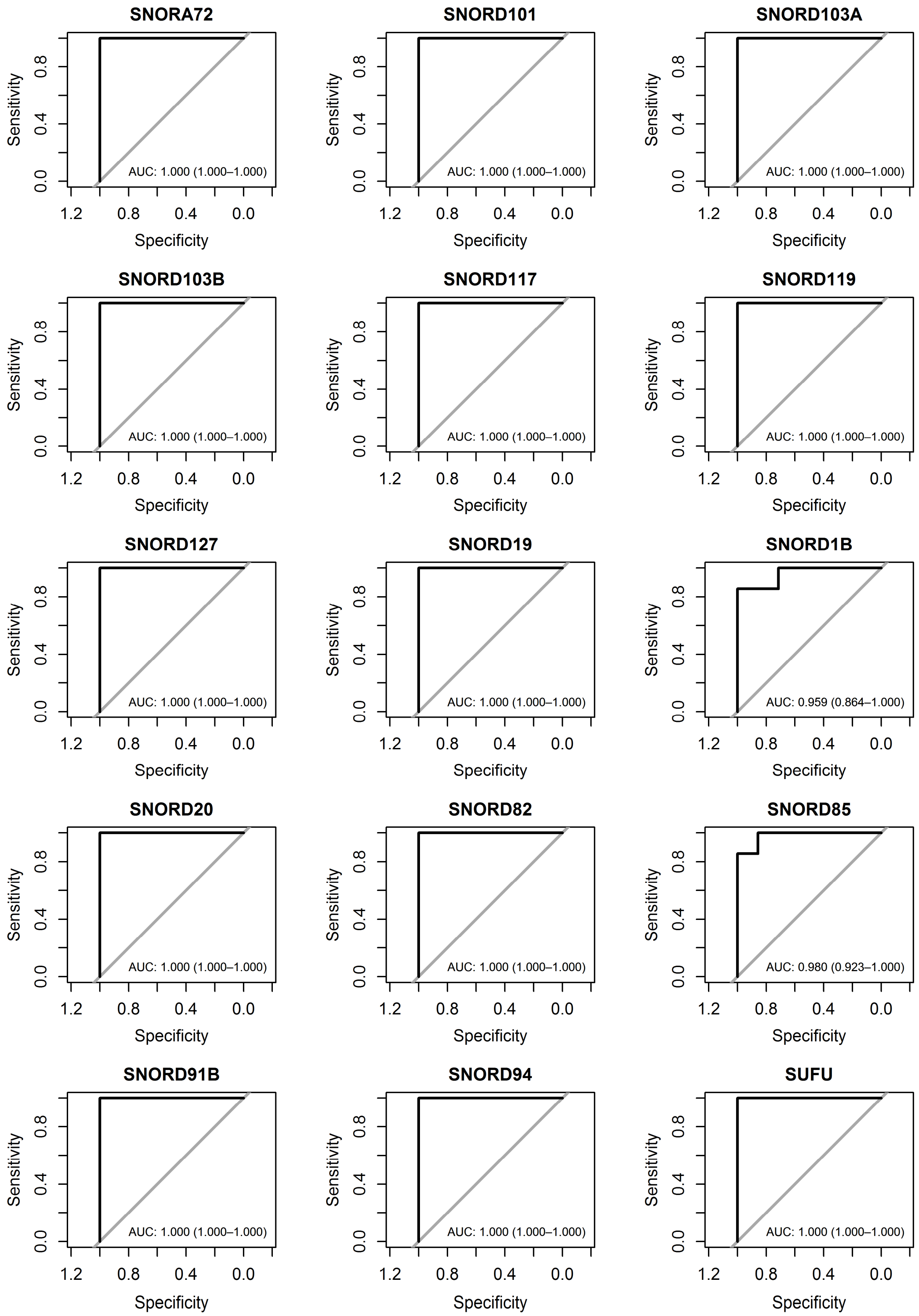

Figure S8. (continued).

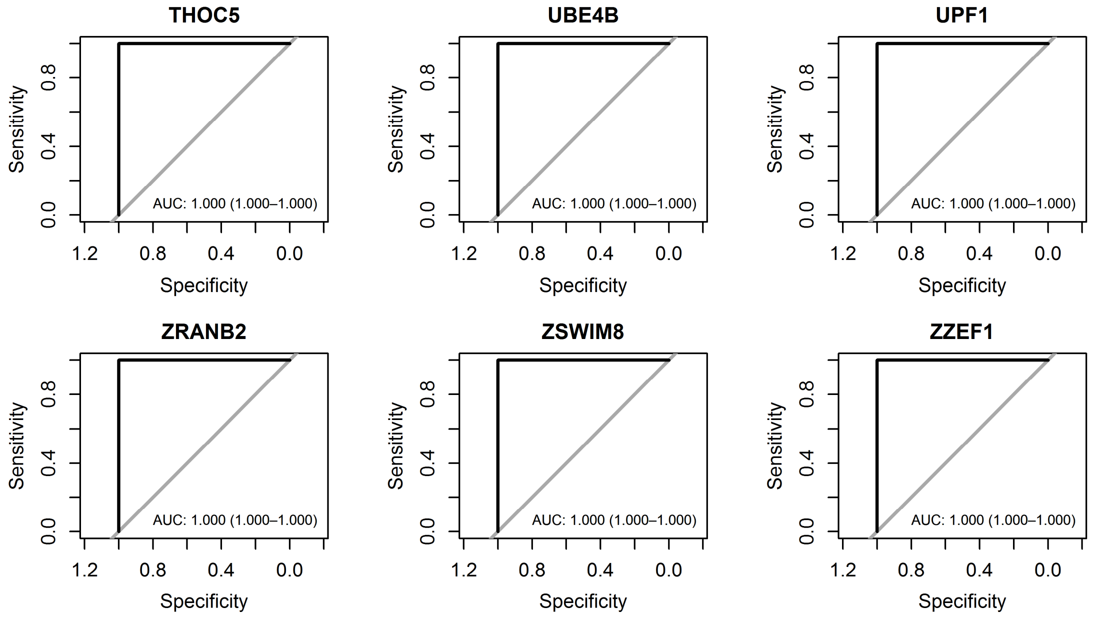

Figure S8. (continued).

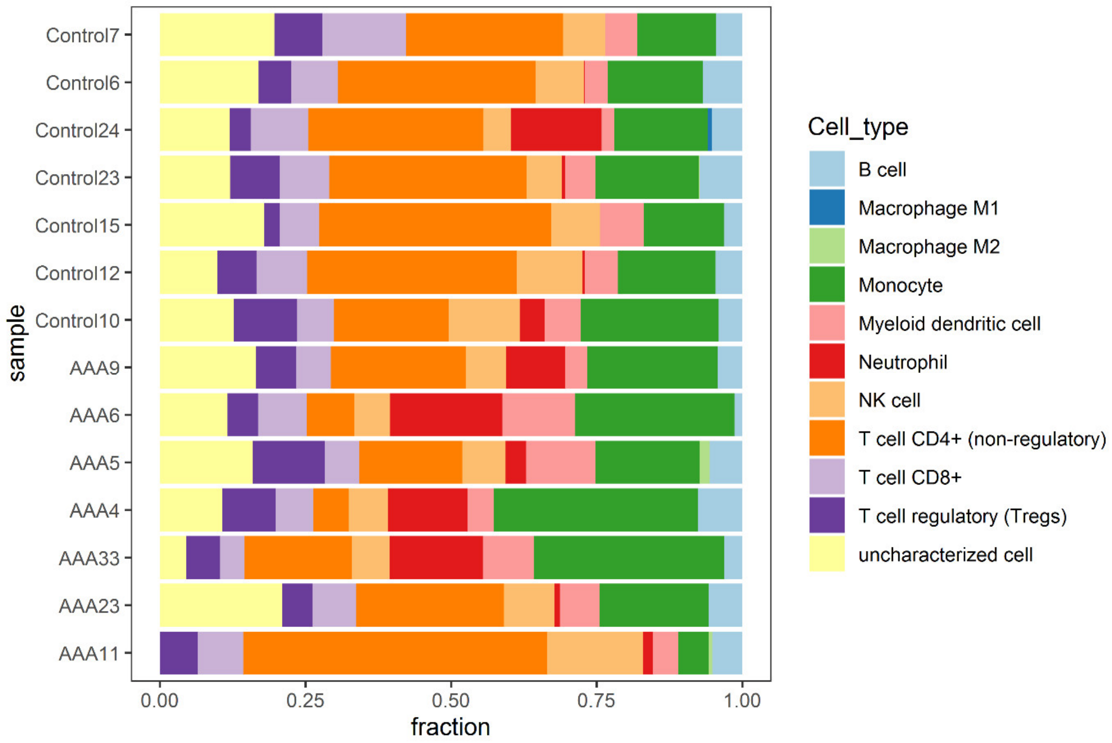

Figure S9. Results of deconvolution procedure performed on gene expression datasets of 7 AAA patients and 7 controls using "quanTIseq" method implemented to immunedeconv 2.0.0 package.

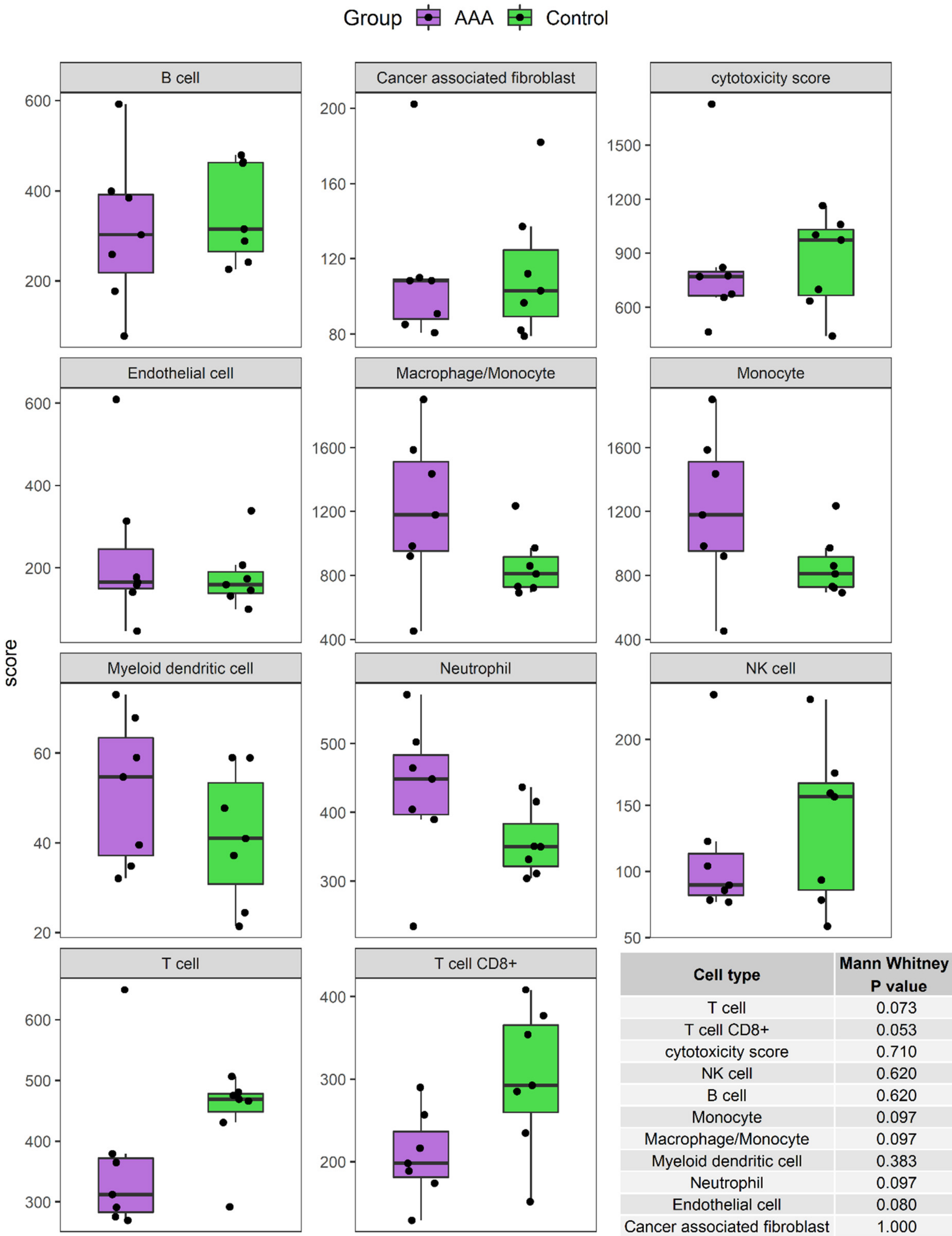

Cell type	Mann Whitney P value
T cell	0.073
T cell CD8+	0.053
cytotoxicity score	0.710
NK cell	0.620
B cell	0.620
Monocyte	0.097
Macrophage/Monocyte	0.097
Myeloid dendritic cell	0.383
Neutrophil	0.097
Endothelial cell	0.080
Cancer associated fibroblast	1.000

Figure S10. Results of deconvolution procedure performed on gene expression datasets of 7 AAA patients (AAA) and 7 controls (control) using "MCPcounter" method implemented to immunedeconv 2.0.0 package. Two-sided Mann–Whitney test (wilcox.test function in R) was used to calculate statistical significance of differences in score values between AAA and the control group.

Supplementary Tables

Table S1. Assessment of small RNA samples, small RNA libraries and results of primary analysis of small RNA sequencing data carried out with Ion Torrent small RNA Plugin v5.0.5r3.

Parameter	Mean ± Standard Deviation	Median
Percentage of microRNA in small RNA samples	19.75 ± 7.98	18
Percentage of 94-114 bp* region in small RNA libraries	51.71 ± 7.61	50
Molar concentration of small RNA libraries (pM)	117,813.3 ± 40,889.62	122,223
Ion Sphere Particles enrichment quality control	31.61% ± 3.91%	31%
Total number of reads	9,941,064.94 ± 3,717,329.33	9,463,792
Reads passing filter	8,950,345.6 ± 3,528,923.77	8,539,195
Number of aligned reads	8,307,255.13 ± 3,403,649.56	8,000,420
Percentage of aligned reads	93.31% ± 0.9%	93.25%
Number of reads aligned to mirBase v21	3,404,903 ± 1,229,633.93	3,541,954
Percentage of reads aligned to mirBase v21	39.58% ± 10.03%	39.96%
Number of reads aligned to precursors	125,943.17 ± 48,756	125,252
microRNAs with 1+ reads	1,152.51 ± 330.62	1,249
microRNAs with 10+ reads	640.6 ± 66.67	653
microRNAs with 100+ reads	330.47 ± 42.82	338
microRNAs with 1,000+ reads	151.64 ± 19.72	156
microRNAs with 10,000+ reads	66.45 ± 12.98	68
Non-uniquely mapped reads	0	0
No feature mappings	611 ± 299.37	556
Ambiguous mappings	0	0
Percentage of coding reads	16.96% ± 4.23%	16.18%
Percentage of rRNA reads	0.71% ± 0.39%	0.59%
Percentage of tRNA reads	8.93% ± 7%	6.79%
Percentage of snoRNA reads	7.93% ± 4.36%	7.1%
Percentage of snRNA reads	0.87% ± 0.23%	0.89%
Percentage of lincRNA reads	1.18% ± 0.36%	1.1%
Percentage of pseudogene reads	0.27% ± 0.06%	0.27%

*base pair

Table S2. Assessment of transcriptome libraries and results of primary analysis of transcriptome sequencing data carried out with Ion Torrent RNASeqAnalysis plugin v.5.0.3.0.

Parameter	Mean ± Standard Deviation	Median
Molar concentration of libraries (pM)	76,306.86 ± 28,426.81	75,300
Percentage of 50-160 bp[*] fragments in libraries	9.14 ± 3.13	11
Ion Sphere Particles enrichment quality control	23.43% ± 5.623%	22%
Total reads	38,585,481.07 ± 4,914,031.8	38,231,595
Aligned reads	37,210,304.6 ± 5,010,816.7	35,910,710
Percent aligned reads	96.37% ± 1.42%	96.96%
Mean read length	115.09 ± 12.15	115.7
Genes Detected	16,387.2 ± 4,268.47	16,989
Isoforms Detected	49,493.07 ± 6,659.32	50,187
Reads mapped to genes	13,687,023.47 ± 5,183,550.03	13,585,070
Genes with 1+ reads	31,199.87 ± 1,245.01	31,036
Genes with 10+ reads	16,320.53 ± 4,221.52	16,989
Genes with 100+ reads	9,035.4 ± 2,620.23	9,749
Genes with 1000+ reads	2,161.67 ± 969.27	2,085
Genes with 10000+ reads	105 ± 46.7	88
Total base reads	4,437,898,797 ± 730,392,105.3	4,212,963,535
Total aligned bases	3,577,284,946 ± 688,794,224	3,397,265,732
Percent aligned bases	80.38% ± 4.18%	79.43%
Percent coding bases	15.81% ± 6.37%	17.95%
Percent UTR[†] bases	27.94% ± 8.33%	29.21%
Percent ribosomal bases	4.72% ± 1.87%	4.79%
Percent intronic bases	38.14% ± 6.48%	36.09%
Percent intergenic bases	14.2% ± 7.71%	10.79%
Strand balance	51.17% ± 1.29%	50.91%

[*]base pair; [†]Untranslated Region

Table S3. The set of 36 differentially expressed miRNA transcripts in the group of 28 AAA individuals compared to 19 healthy controls, resulted from DESeq2 analysis with $p < 0.0001$. MiRNA transcripts were ordered according to increasing p value.

No.	microRNA transcript	p value	Fold change
1.	hsa-mir-31_hsa-miR-31-5p	4.184E-12	0.344
2.	hsa-mir-31_hsa-miR-31-3p	4.184E-12	0.329
3.	hsa-mir-21_hsa-miR-21-5p	9.194E-12	1.356
4.	hsa-mir-874_hsa-miR-874-5p	7.390E-11	0.429
5.	hsa-mir-361_hsa-miR-361-3p	8.258E-10	0.683
6.	hsa-mir-124-2_hsa-miR-124-3p	1.557E-09	12.896
7.	hsa-mir-21_hsa-miR-21-3p	1.733E-09	1.704
8.	hsa-mir-34a_hsa-miR-34a-5p	5.608E-09	2.188
9.	hsa-mir-454_hsa-miR-454-3p	2.736E-08	1.216
10.	hsa-mir-342_hsa-miR-342-3p	1.219E-07	0.592
11.	hsa-mir-124-1_hsa-miR-124-3p	3.040E-07	8.898
12.	hsa-mir-138-1_hsa-miR-138-5p	3.649E-07	0.368
13.	hsa-mir-574_hsa-miR-574-5p	1.129E-06	1.364
14.	hsa-mir-125b-2_hsa-miR-125b-5p	1.321E-06	0.552
15.	hsa-mir-150_hsa-miR-150-5p	1.884E-06	0.581
16.	hsa-mir-424_hsa-miR-424-3p	2.029E-06	1.872
17.	hsa-mir-3607_hsa-miR-3607-5p	2.029E-06	0.532
18.	hsa-mir-450b_hsa-miR-450b-5p	2.758E-06	1.834
19.	hsa-mir-769_hsa-miR-769-5p	5.363E-06	0.813
20.	hsa-let-7g_hsa-let-7g-3p	7.340E-06	0.750
21.	hsa-mir-125b-1_hsa-miR-125b-5p	7.340E-06	0.560
22.	hsa-mir-24-2_hsa-miR-24-3p	8.592E-06	1.143
23.	hsa-mir-34a_hsa-miR-34a-3p	1.422E-05	2.357
24.	hsa-mir-124-3_hsa-miR-124-3p	1.983E-05	7.173
25.	hsa-mir-138-2_hsa-miR-138-5p	2.466E-05	0.397
26.	hsa-mir-542_hsa-miR-542-3p	4.141E-05	1.666
27.	hsa-mir-339_hsa-miR-339-3p	4.314E-05	0.770
28.	hsa-mir-5585_hsa-miR-5585-3p	4.641E-05	0.396
29.	hsa-mir-503_hsa-miR-503-5p	6.916E-05	1.781
30.	hsa-mir-99a_hsa-miR-99a-3p	6.916E-05	0.481
31.	hsa-mir-7847_hsa-miR-7847-3p	6.999E-05	2.270
32.	hsa-mir-548d-1_hsa-miR-548d-3p	7.095E-05	1.493
33.	hsa-mir-122_hsa-miR-122-5p	7.937E-05	1.790
34.	hsa-mir-3591_hsa-miR-3591-3p	7.937E-05	1.789
35.	hsa-mir-766_hsa-miR-766-3p	8.720E-05	0.808
36.	hsa-mir-424_hsa-miR-424-5p	9.561E-05	1.579

Table S4. The set of 75 differentially expressed miRNA transcripts in the group of 28 AAA individuals compared to 19 healthy controls, resulted from UVE-PLS analysis. MiRNA transcripts were ordered according to decreasing PLS coefficients.

No	microRNA transcript	PLS coefficient
1.	hsa-mir-34a_hsa-miR-34a-5p	4.040E-02
2.	hsa-mir-196a-2_hsa-miR-196a-5p	3.455E-02
3.	hsa-mir-21_hsa-miR-21-3p	2.766E-02
4.	hsa-mir-424_hsa-miR-424-5p	2.614E-02
5.	hsa-mir-450b_hsa-miR-450b-5p	2.542E-02
6.	hsa-mir-1277_hsa-miR-1277-3p	2.457E-02
7.	hsa-mir-7847_hsa-miR-7847-3p	2.449E-02
8.	hsa-mir-34a_hsa-miR-34a-3p	2.421E-02
9.	hsa-mir-1268b_hsa-miR-1268b	2.291E-02
10.	hsa-mir-490_hsa-miR-490-5p	2.261E-02
11.	hsa-mir-503_hsa-miR-503-5p	1.989E-02
12.	hsa-mir-3591_hsa-miR-3591-3p	1.880E-02
13.	hsa-mir-122_hsa-miR-122-5p	1.880E-02
14.	hsa-mir-542_hsa-miR-542-3p	1.856E-02
15.	hsa-mir-1249_hsa-miR-1249	1.825E-02
16.	hsa-mir-548e_hsa-miR-548e-3p	1.822E-02
17.	hsa-mir-424_hsa-miR-424-3p	1.789E-02
18.	hsa-mir-574_hsa-miR-574-5p	1.654E-02
19.	hsa-mir-21_hsa-miR-21-5p	1.606E-02
20.	hsa-mir-497_hsa-miR-497-5p	1.494E-02
21.	hsa-mir-7977_hsa-miR-7977	1.487E-02
22.	hsa-mir-548f-2_hsa-miR-548f-3p	1.411E-02
23.	hsa-mir-548d-2_hsa-miR-548d-3p	1.374E-02
24.	hsa-mir-450a-1_hsa-miR-450a-1-3p	1.361E-02
25.	hsa-mir-22_hsa-miR-22-3p	1.323E-02
26.	hsa-mir-556_hsa-miR-556-5p	1.308E-02
27.	hsa-let-7b_hsa-let-7b-3p	1.294E-02
28.	hsa-mir-629_hsa-miR-629-5p	1.149E-02
29.	hsa-mir-454_hsa-miR-454-3p	1.145E-02
30.	hsa-mir-548d-1_hsa-miR-548d-3p	9.306E-03
31.	hsa-mir-877_hsa-miR-877-5p	8.935E-03
32.	hsa-mir-24-2_hsa-miR-24-3p	6.771E-03
33.	hsa-mir-4286_hsa-miR-4286	6.004E-03
34.	hsa-mir-25_hsa-miR-25-3p	-3.998E-03
35.	hsa-mir-192_hsa-miR-192-5p	-6.755E-03
36.	hsa-mir-30e_hsa-miR-30e-3p	-6.771E-03
37.	hsa-mir-19b-1_hsa-miR-19b-1-5p	-7.332E-03
38.	hsa-mir-141_hsa-miR-141-3p	-7.473E-03
39.	hsa-mir-330_hsa-miR-330-3p	-7.602E-03
40.	hsa-mir-92a-1_hsa-miR-92a-3p	-7.729E-03
41.	hsa-mir-106b_hsa-miR-106b-3p	-7.780E-03
42.	hsa-mir-140_hsa-miR-140-3p	-8.818E-03
43.	hsa-mir-181a-2_hsa-miR-181a-5p	-8.821E-03
44.	hsa-mir-92a-2_hsa-miR-92a-3p	-8.857E-03
45.	hsa-mir-342_hsa-miR-342-5p	-9.110E-03
46.	hsa-mir-671_hsa-miR-671-5p	-9.352E-03

47.	hsa-mir-769_hsa-miR-769-5p	-9.440E-03
48.	hsa-mir-339_hsa-miR-339-3p	-1.003E-02
49.	hsa-mir-30d_hsa-miR-30d-5p	-1.009E-02
50.	hsa-mir-181c_hsa-miR-181c-3p	-1.041E-02
51.	hsa-mir-194-1_hsa-miR-194-5p	-1.047E-02
52.	hsa-mir-664b_hsa-miR-664b-3p	-1.059E-02
53.	hsa-let-7g_hsa-let-7g-3p	-1.155E-02
54.	hsa-mir-181c_hsa-miR-181c-5p	-1.173E-02
55.	hsa-mir-30e_hsa-miR-30e-5p	-1.266E-02
56.	hsa-mir-5701-2_hsa-miR-5701	-1.294E-02
57.	hsa-mir-1275_hsa-miR-1275	-1.592E-02
58.	hsa-mir-766_hsa-miR-766-3p	-1.626E-02
59.	hsa-mir-361_hsa-miR-361-3p	-1.814E-02
60.	hsa-mir-5585_hsa-miR-5585-3p	-1.910E-02
61.	hsa-mir-342_hsa-miR-342-3p	-1.938E-02
62.	hsa-mir-150_hsa-miR-150-5p	-2.043E-02
63.	hsa-mir-193b_hsa-miR-193b-3p	-2.294E-02
64.	hsa-mir-5701-1_hsa-miR-5701	-2.346E-02
65.	hsa-mir-125b-1_hsa-miR-125b-5p	-2.352E-02
66.	hsa-mir-99a_hsa-miR-99a-3p	-2.377E-02
67.	hsa-mir-125b-2_hsa-miR-125b-5p	-2.562E-02
68.	hsa-mir-3607_hsa-miR-3607-5p	-2.860E-02
69.	hsa-mir-874_hsa-miR-874-5p	-3.331E-02
70.	hsa-mir-4284_hsa-miR-4284	-3.462E-02
71.	hsa-mir-138-2_hsa-miR-138-5p	-3.775E-02
72.	hsa-mir-5193_hsa-miR-5193	-4.051E-02
73.	hsa-mir-138-1_hsa-miR-138-5p	-4.276E-02
74.	hsa-mir-31_hsa-miR-31-5p	-4.970E-02
75.	hsa-mir-31_hsa-miR-31-3p	-5.269E-02

Table S5. Results of ROC analysis for 34 miRNA transcripts selected as signatures for AAA.

miRNA transcript	ROC-AUC[1]	Threshold	Specificity	Sensitivity	Accuracy	Positive Predictive Value	Negative Predictive Value
hsa-mir-31_hsa-miR-31-5p	0.981	9.850	0.964	0.895	0.936	0.944	0.931
hsa-mir-31_hsa-miR-31-3p	0.970	7.063	0.929	0.895	0.915	0.895	0.929
hsa-mir-21_hsa-miR-21-5p	0.953	17.731	0.929	0.895	0.915	0.895	0.929
hsa-mir-361_hsa-miR-361-3p	0.945	11.566	0.929	0.842	0.894	0.889	0.897
hsa-mir-454_hsa-miR-454-3p	0.940	10.313	0.929	0.842	0.894	0.889	0.897
hsa-mir-874_hsa-miR-874-5p	0.934	3.335	0.929	0.947	0.936	0.900	0.963
hsa-mir-34a_hsa-miR-34a-5p	0.927	8.810	0.929	0.789	0.872	0.882	0.867
hsa-mir-342_hsa-miR-342-3p	0.923	15.106	0.821	0.895	0.851	0.773	0.920
hsa-mir-21_hsa-miR-21-3p	0.919	11.164	0.750	0.947	0.830	0.720	0.955
hsa-mir-150_hsa-miR-150-5p	0.906	16.984	0.821	0.947	0.872	0.783	0.958
hsa-mir-574_hsa-miR-574-5p	0.898	8.367	0.679	1.000	0.809	0.679	1.000
hsa-let-7g_hsa-let-7g-3p	0.887	7.996	0.821	0.947	0.872	0.783	0.958
hsa-mir-3607_hsa-miR-3607-5p	0.880	6.876	0.964	0.632	0.830	0.923	0.794
hsa-mir-24-2_hsa-miR-24-3p	0.874	13.716	0.786	0.895	0.830	0.739	0.917
hsa-mir-769_hsa-miR-769-5p	0.874	9.063	0.857	0.842	0.851	0.800	0.889
hsa-mir-450b_hsa-miR-450b-5p	0.872	5.337	0.857	0.789	0.830	0.789	0.857
hsa-mir-125b-2_hsa-miR-125b-5p	0.868	7.613	0.679	0.947	0.787	0.667	0.950
hsa-mir-339_hsa-miR-339-3p	0.868	7.157	0.821	0.842	0.830	0.762	0.885
hsa-mir-34a_hsa-miR-34a-3p	0.867	3.672	0.750	0.842	0.787	0.696	0.875
hsa-mir-138-2_hsa-miR-138-5p	0.863	3.720	0.857	0.789	0.830	0.789	0.857
hsa-mir-7847_hsa-miR-7847-3p	0.861	2.483	0.857	0.737	0.809	0.778	0.828
hsa-mir-424_hsa-miR-424-3p	0.861	6.758	0.679	0.947	0.787	0.667	0.950
hsa-mir-125b-1_hsa-miR-125b-5p	0.857	7.844	0.821	0.842	0.830	0.762	0.885
hsa-mir-99a_hsa-miR-99a-3p	0.853	3.515	0.857	0.789	0.830	0.789	0.857
hsa-mir-138-1_hsa-miR-138-5p	0.852	4.655	0.964	0.632	0.830	0.923	0.794
hsa-mir-766_hsa-miR-766-3p	0.852	10.392	0.714	0.895	0.787	0.680	0.909
hsa-mir-542_hsa-miR-542-3p	0.852	7.027	0.786	0.842	0.809	0.727	0.880
hsa-mir-548d-1_hsa-miR-548d-3p	0.848	4.366	0.893	0.684	0.809	0.813	0.806
hsa-mir-503_hsa-miR-503-5p	0.821	9.752	0.714	0.895	0.787	0.680	0.909
hsa-mir-424_hsa-miR-424-5p	0.810	11.422	0.821	0.789	0.809	0.750	0.852
hsa-mir-5585_hsa-miR-5585-3p	0.801	2.587	0.786	0.842	0.809	0.727	0.880
hsa-mir-122_hsa-miR-122-5p	0.795	8.597	0.571	1.000	0.745	0.613	1.000
hsa-mir-3591_hsa-miR-3591-3p	0.795	8.597	0.571	1.000	0.745	0.613	1.000

[1]Area under ROC curve.

Table S6. Results of DESeq2 differential analysis of genes in the group of 7 AAA individuals compared to the group of 7 healthy controls. The table presents 155 differentially expressed genes with $p < 0.0001$, ordered accordingly to increasing p value. Gene names were searched using HUGO multi-symbol checker and gene symbols not assigned to names are termed as "unmatched". Synonyms or previous gene symbols are placed in brackets.

No.	Gene symbol	Gene name	*p* value	Fold change
1.	*NRCAM*	neuronal cell adhesion molecule	2.133E-12	0.087
2.	*SNORA60*	small nucleolar RNA, H/ACA box 60	1.192E-11	0.547
3.	*TBC1D22A*	TBC1 domain family member 22A	2.090E-11	1.473
4.	*NOG*	noggin	1.067E-10	0.188
5.	*CPT1A*	carnitine palmitoyltransferase 1A	1.703E-10	2.487
6.	*CD248*	CD248 molecule	3.625E-10	0.177
7.	*SNORD7*	small nucleolar RNA, C/D box 7	3.625E-10	0.399
8.	*MIRLET7F2*	microRNA let-7f-2	4.894E-10	0.285
9.	*SNHG5*	small nucleolar RNA host gene 5	5.053E-10	0.433
10.	*SNORD20*	small nucleolar RNA, C/D box 20	7.751E-10	0.235
11.	*SNORA72*	small nucleolar RNA, H/ACA box 72	3.716E-09	0.358
12.	*SCARNA1*	small Cajal body-specific RNA 1	6.279E-09	0.442
13.	*GGT1*	gamma-glutamyltransferase 1	1.107E-08	1.973
14.	*RP11-426C22.5*	Unmatched	1.107E-08	2.351
15.	*SNORD117*	small nucleolar RNA, C/D box 117	1.107E-08	0.457
16.	*TSC2*	TSC complex subunit 2	1.107E-08	1.374
17.	*SNORD82*	small nucleolar RNA, C/D box 82	1.171E-08	0.357
18.	*ARF1P2*	ADP ribosylation factor 1 pseudogene 2	1.933E-08	2.053
19.	*SNORA36B*	small nucleolar RNA, H/ACA box 36B	2.150E-08	0.633
20.	*UPF1*	UPF1 RNA helicase and ATPase	2.426E-08	1.321
21.	*POLR2A*	RNA polymerase II subunit A	2.502E-08	1.453
22.	*ZNF24*	zinc finger protein 24	5.009E-08	0.749
23.	*SNORD94*	small nucleolar RNA, C/D box 94	5.103E-08	0.387
24.	*PLEKHG2*	pleckstrin homology and RhoGEF domain containing G2	5.434E-08	1.592
25.	*SNORD101*	small nucleolar RNA, C/D box 101	5.434E-08	0.330
26.	*SNORA11*	small nucleolar RNA, H/ACA box 11	6.243E-08	0.442
27.	*SNORA2C (SNORA34)*	small nucleolar RNA, H/ACA box 2C	6.468E-08	0.572
28.	*RNA5SP355*	RNA, 5S ribosomal pseudogene 355	8.243E-08	0.053
29.	*SNORD124*	small nucleolar RNA, C/D box 124	9.220E-08	0.340
30.	*SNORD103C (SNORD85)*	small nucleolar RNA, C/D box 103C	1.340E-07	0.342
31.	*EEF1A1P24*	eukaryotic translation elongation factor 1 alpha 1 pseudogene 24	1.860E-07	0.331
32.	*SNORD64*	small nucleolar RNA, C/D box 64	1.860E-07	0.324
33.	*TRAPPC9*	trafficking protein particle complex 9	1.860E-07	1.480
34.	*RPL3P9*	ribosomal protein L3 pseudogene 9	1.868E-07	0.260
35.	*RP11-16F15.2*	Unmatched	2.060E-07	0.344
36.	*RP11-302F12.1*	Unmatched	2.251E-07	0.194
37.	*UVSSA*	UV stimulated scaffold protein A	3.311E-07	1.524

38.	*SNORA28*	small nucleolar RNA, H/ACA box 28	3.334E-07	0.511
39.	*C1orf87*	chromosome 1 open reading frame 87	4.334E-07	0.012
40.	*SNORA12*	small nucleolar RNA, H/ACA box 12	4.916E-07	0.631
41.	*NATD1 (C17orf103)*	N-acetyltransferase domain containing 1	6.334E-07	1.853
42.	*RRN3P2*	RRN3 homolog, RNA polymerase I transcription factor pseudogene 2	6.442E-07	1.692
43.	*SNORA33*	small nucleolar RNA, H/ACA box 33	6.822E-07	0.633
44.	*SNORD111B*	small nucleolar RNA, C/D box 111B	7.813E-07	0.409
45.	*ZRANB2*	zinc finger RANBP2-type containing 2	7.813E-07	0.710
46.	*AC092620.2*	Unmatched	8.851E-07	2.867
47.	*CCDC17*	coiled-coil domain containing 17	9.018E-07	1.731
48.	*SNORD91B*	small nucleolar RNA, C/D box 91B	9.723E-07	0.324
49.	*TSGA10*	testis specific 10	9.723E-07	0.266
50.	*RP11-253E3.1*	Unmatched	1.325E-06	0.315
51.	*SNORA3B (SNORA45)*	small nucleolar RNA, H/ACA box 3B	1.775E-06	0.537
52.	*LINC01016*	long intergenic non-protein coding RNA 1016	2.283E-06	0.135
53.	*SNORD103B*	small nucleolar RNA, C/D box 103B	2.344E-06	0.338
54.	*SNORA2B*	small nucleolar RNA, H/ACA box 2B	2.654E-06	0.540
55.	*PLEKHM1P1 (PLEKHM1P)*	pleckstrin homology and RUN domain containing M1 pseudogene 1	2.989E-06	1.637
56.	*SATB1*	SATB homeobox 1	3.357E-06	0.622
57.	*SNORD127*	small nucleolar RNA, C/D box 127	3.357E-06	0.511
58.	*SNORD103A*	small nucleolar RNA, C/D box 103A	4.061E-06	0.354
59.	*SCARNA13*	small Cajal body-specific RNA 13	4.128E-06	0.689
60.	*MEP1AP4*	meprin A subunit alpha pseudogene 4	4.151E-06	0.046
61.	*SNORD12C*	small nucleolar RNA, C/D box 12C	4.576E-06	0.557
62.	*SNORA14B*	small nucleolar RNA, H/ACA box 14B	4.659E-06	0.592
63.	*KIAA1549L*	KIAA1549 like	5.443E-06	0.178
64.	*SNORD119*	small nucleolar RNA, C/D box 119	5.577E-06	0.427
65.	*UBE4B*	ubiquitination factor E4B	5.725E-06	1.300
66.	*SEC14L2*	SEC14 like lipid binding 2	6.286E-06	0.296
67.	*B3GNTL1*	UDP-GlcNAc:betaGal beta-1,3-N-acetylglucosaminyltransferase like 1	6.319E-06	1.612
68.	*SNORA58*	small nucleolar RNA, H/ACA box 58	6.319E-06	0.628
69.	*RN7SKP227*	RN7SK pseudogene 227	6.454E-06	0.295
70.	*MIR199A2*	microRNA 199a-2	6.562E-06	0.143
71.	*SNORD63*	small nucleolar RNA, C/D box 63	7.160E-06	0.363
72.	*RAD21*	RAD21 cohesin complex component	7.191E-06	0.781
73.	*ZNF542P (ZNF542)*	zinc finger protein 542, pseudogene	7.727E-06	0.568
74.	*HBA2*	hemoglobin subunit alpha 2	7.849E-06	4.412
75.	*CYP26C1*	cytochrome P450 family 26 subfamily C member 1	8.646E-06	0.201
76.	*PKDCC*	protein kinase domain containing, cytoplasmic	9.257E-06	0.141
77.	*PDCD4*	programmed cell death 4	9.397E-06	0.654
78.	*MIR181A1*	microRNA 181a-1	9.422E-06	0.112
79.	*SLC36A1*	solute carrier family 36 member 1	9.422E-06	1.884
80.	*CDKN1B*	cyclin dependent kinase inhibitor 1B	9.572E-06	0.700
81.	*HTT*	huntingtin	1.123E-05	1.388
82.	*HSP90AB2P*	heat shock protein 90 alpha family class B member 2, pseudogene	1.132E-05	0.262

83.	*B3GNT7*	UDP-GlcNAc:betaGal beta-1,3-N-acetylglucosaminyltransferase 7	1.142E-05	0.285
84.	*SCARNA9*	small Cajal body-specific RNA 9	1.142E-05	0.539
85.	*RP1-102E24.1*	Unmatched	1.187E-05	0.315
86.	*SMNDC1*	survival motor neuron domain containing 1	1.235E-05	0.685
87.	*NBEAL2*	neurobeachin like 2	1.378E-05	1.517
88.	*SMG1P1*	SMG1 pseudogene 1	1.771E-05	1.598
89.	*NUP214*	nucleoporin 214	1.827E-05	1.344
90.	*GIT2*	GIT ArfGAP 2	2.082E-05	1.449
91.	*KLHL21*	kelch like family member 21	2.433E-05	1.422
92.	*PRDM13*	PR/SET domain 13	2.464E-05	0.140
93.	*HEMGN*	hemogen	2.519E-05	0.496
94.	*MIR26A1*	microRNA 26a-1	2.519E-05	0.156
95.	*RP11-738O11.13*	Unmatched	2.519E-05	0.078
96.	*SFRP5*	secreted frizzled related protein 5	2.615E-05	0.087
97.	*SNORA71B*	small nucleolar RNA, H/ACA box 71B	2.699E-05	0.527
98.	*CSAD*	cysteine sulfinic acid decarboxylase	2.721E-05	1.554
99.	*THOC5*	THO complex 5	2.724E-05	1.319
100.	*SNORD126*	small nucleolar RNA, C/D box 126	2.737E-05	0.492
101.	*ZZEF1*	zinc finger ZZ-type and EF-hand domain containing 1	2.817E-05	1.291
102.	*RPS4XP3*	ribosomal protein S4X pseudogene 3	2.875E-05	0.417
103.	*SNORD104*	small nucleolar RNA, C/D box 104	2.875E-05	0.618
104.	*ANKRD13D*	ankyrin repeat domain 13D	3.010E-05	1.428
105.	*RP3-449O17.1*	Unmatched	3.010E-05	1.907
106.	*SNORA46*	small nucleolar RNA, H/ACA box 46	3.010E-05	0.672
107.	*SNORD19*	small nucleolar RNA, C/D box 19	3.455E-05	0.541
108.	*DLGAP2 (ERICH1-AS1)*	DLG associated protein 2	3.559E-05	0.014
109.	*MAU2*	MAU2 sister chromatid cohesion factor	3.666E-05	1.306
110.	*RPL21P39*	ribosomal protein L21 pseudogene 39	3.666E-05	0.456
111.	*SNORA22*	small nucleolar RNA, H/ACA box 22	3.666E-05	0.536
112.	*SNORA26*	small nucleolar RNA, H/ACA box 26	3.666E-05	0.425
113.	*MIR144*	microRNA 144	3.819E-05	0.254
114.	*PDE9A*	phosphodiesterase 9A	3.819E-05	0.346
115.	*SLC16A10*	solute carrier family 16 member 10	3.819E-05	0.402
116.	*FAM157C*	family with sequence similarity 157 member C	3.835E-05	2.617
117.	*SUFU*	SUFU negative regulator of hedgehog signaling	4.105E-05	1.482
118.	*RP11-624L12.1*	Unmatched	4.189E-05	0.360
119.	*RN7SKP89*	RN7SK pseudogene 89	4.454E-05	2.764
120.	*SNORD111*	small nucleolar RNA, C/D box 111	4.511E-05	0.581
121.	*RNU2-36P*	RNA, U2 small nuclear 36, pseudogene	4.802E-05	0.401
122.	*SNORA50A (SNORA50)*	small nucleolar RNA, H/ACA box 50A	4.843E-05	0.475
123.	*U73166.2*	Unmatched	5.217E-05	2.348
124.	*SNORA40*	small nucleolar RNA, H/ACA box 40	5.310E-05	0.394
125.	*TRIP6*	thyroid hormone receptor interactor 6	5.496E-05	1.881
126.	*MEP1AP3*	meprin A subunit alpha pseudogene 3	5.520E-05	0.036
127.	*ZSWIM8*	zinc finger SWIM-type containing 8	5.520E-05	1.355
128.	*HBB*	hemoglobin subunit beta	5.667E-05	3.804
129.	*ADGRA3 (GPR125)*	adhesion G protein-coupled receptor A3	5.839E-05	0.418

130.	*SNORA74*	Unmatched	5.839E-05	0.512
131.	*RPL21P75*	ribosomal protein L21 pseudogene 75	5.919E-05	0.514
132.	*E2F2*	E2F transcription factor 2	5.941E-05	1.958
133.	*EEA1*	early endosome antigen 1	5.941E-05	0.770
134.	*EHMT1*	euchromatic histone lysine methyltransferase 1	5.941E-05	1.363
135.	*NKX2-5*	NK2 homeobox 5	6.070E-05	0.149
136.	*SNORD11B*	small nucleolar RNA, C/D box 11B	6.252E-05	0.499
137.	*RP11-158G18.1*	Unmatched	6.326E-05	0.461
138.	*RPL4P6*	ribosomal protein L4 pseudogene 6	6.736E-05	2.110
139.	*SNORD51*	small nucleolar RNA, C/D box 51	7.423E-05	0.392
140.	*SLC4A10*	solute carrier family 4 member 10	7.999E-05	0.261
141.	*RP11-118B13.1*	Unmatched	8.397E-05	0.207
142.	*TYRO3P*	TYRO3P protein tyrosine kinase pseudogene	8.435E-05	2.361
143.	*GDF2*	growth differentiation factor 2	8.618E-05	0.107
144.	*RN7SKP62*	RN7SK pseudogene 62	8.618E-05	0.373
145.	*MEP1AP1*	meprin A subunit alpha pseudogene 1	8.825E-05	0.030
146.	*SNORD1B*	small nucleolar RNA, C/D box 1B	8.825E-05	0.333
147.	*TMIGD2*	transmembrane and immunoglobulin domain containing 2	8.825E-05	0.450
148.	*RP4-756H11.1*	Unmatched	9.031E-05	0.036
149.	*SNORD14E*	small nucleolar RNA, C/D box 14E	9.208E-05	0.418
150.	*RP11-216N14.7*	Unmatched	9.246E-05	1.939
151.	*RP11-291B21.2*	Unmatched	9.346E-05	0.390
152.	*AGO2*	argonaute RISC catalytic component 2	9.738E-05	1.401
153.	*EEF1A1P12*	eukaryotic translation elongation factor 1 alpha 1 pseudogene 12	9.738E-05	0.557
154.	*ABCC1*	ATP binding cassette subfamily C member 1	9.989E-05	1.394
155.	*ACTG1P3*	actin gamma 1 pseudogene 3	9.989E-05	1.765

Table S7. Results of UVE-PLS analysis of miRNA in the group of 7 AAA individuals compared to the group of 7 healthy controls. The table presents 91 differentially expressed genes recognized as informative, ordered according to decreasing PLS coefficients. Gene names were searched using the HUGO multi-symbol checker and gene symbols not assigned to names are termed as "unmatched". Synonyms or previous gene symbols are placed in brackets.

No.	Gene symbol	Gene name	PLS coefficient
1.	*CPT1A*	carnitine palmitoyltransferase 1A	1.567E-03
2.	*AC092620.2*	Unmatched	1.239E-03
3.	*GGT1*	gamma-glutamyltransferase 1	9.424E-04
4.	*RN7SKP89*	RN7SK pseudogene 89	8.740E-04
5.	*NBEAL2*	neurobeachin like 2	5.590E-04
6.	*SUFU*	SUFU negative regulator of hedgehog signaling	5.397E-04
7.	*GIT2*	GIT ArfGAP 2	5.331E-04
8.	*C1RL*	complement C1r subcomponent like	4.919E-04
9.	*ANKRD13D*	ankyrin repeat domain 13D	4.791E-04
10.	*MED20*	mediator complex subunit 20	4.665E-04
11.	*HTT*	huntingtin	4.526E-04
12.	*UPF1*	UPF1 RNA helicase and ATPase	4.369E-04
13.	*ZNF592*	zinc finger protein 592	4.250E-04
14.	*ZSWIM8*	zinc finger SWIM-type containing 8	4.127E-04
15.	*THOC5*	THO complex 5	4.027E-04
16.	*LIMD1*	LIM domains containing 1	3.966E-04
17.	*UBE4B*	ubiquitination factor E4B	3.839E-04
18.	*CAMTA2*	calmodulin binding transcription activator 2	3.737E-04
19.	*TUBGCP3*	tubulin gamma complex associated protein 3	3.620E-04
20.	*ECH1*	enoyl-CoA hydratase 1	3.440E-04
21.	*ZZEF1*	zinc finger ZZ-type and EF-hand domain containing 1	3.325E-04
22.	*MAN1B1*	mannosidase alpha class 1B member 1	3.299E-04
23.	*DPP3*	dipeptidyl peptidase 3	3.229E-04
24.	*GPAA1*	glycosylphosphatidylinositol anchor attachment 1	3.177E-04
25.	*NCDN*	neurochondrin	2.728E-04
26.	*AC017080.1*	Unmatched	-4.017E-04
27.	*SNORA5A*	small nucleolar RNA, H/ACA box 5A	-4.092E-04
28.	*SNORA49*	small nucleolar RNA, H/ACA box 49	-4.112E-04
29.	*RPS4XP8*	ribosomal protein S4X pseudogene 8	-4.574E-04
30.	*FOXQ1*	forkhead box Q1	-4.799E-04
31.	*SCARNA13*	small Cajal body-specific RNA 13	-4.895E-04
32.	*RP11-766F14.2*	Unmatched	-5.035E-04
33.	*ZRANB2*	zinc finger RANBP2-type containing 2	-5.094E-04
34.	*GS1-251I9.4*	OTUD6B antisense RNA 1 (head to head)	-5.162E-04
35.	*PDCD4*	programmed cell death 4	-5.442E-04
36.	*SNORD109B*	small nucleolar RNA, C/D box 109B	-6.057E-04
37.	*ZXDB*	zinc finger X-linked duplicated B	-6.316E-04
38.	*AC005884.1*	Unmatched	-6.505E-04
39.	*ALDH1A3*	aldehyde dehydrogenase 1 family member A3	-6.710E-04
40.	*MIR4453*	microRNA 4453	-6.749E-04
41.	*SNORA33*	small nucleolar RNA, H/ACA box 33	-7.151E-04

42.	*SNORA12*	small nucleolar RNA, H/ACA box 12	-7.161E-04
43.	*SNORA14B*	small nucleolar RNA, H/ACA box 14B	-7.310E-04
44.	*SNORA36C*	small nucleolar RNA, H/ACA box 36C	-7.518E-04
45.	*SNORD19*	small nucleolar RNA, C/D box 19	-7.859E-04
46.	*RPS9P1*	ribosomal protein S9 pseudogene 1	-7.930E-04
47.	*SCARNA9*	small Cajal body-specific RNA 9	-8.278E-04
48.	*SNORD66*	small nucleolar RNA, C/D box 66	-8.802E-04
49.	*RNA5SP298*	RNA, 5S ribosomal pseudogene 298	-8.819E-04
50.	*SNORA50A (SNORA50)*	small nucleolar RNA, H/ACA box 50A	-9.165E-04
51.	*RP11-40C6.2*	Unmatched	-9.359E-04
52.	*RNU2-36P*	RNA, U2 small nuclear 36, pseudogene	-9.650E-04
53.	*SNORD127*	small nucleolar RNA, C/D box 127	-1.002E-03
54.	*RP1-102E24.1*	Unmatched	-1.006E-03
55.	*RP11-253E3.1*	Unmatched	-1.007E-03
56.	*SNORD119*	small nucleolar RNA, C/D box 119	-1.048E-03
57.	*RP11-16F15.2*	Unmatched	-1.054E-03
58.	*SNORA40*	small nucleolar RNA, H/ACA box 40	-1.075E-03
59.	*SNORA60*	small nucleolar RNA, H/ACA box 60	-1.082E-03
60.	*RNA5-8SP5*	RNA, 5.8S ribosomal pseudogene 5	-1.137E-03
61.	*RNA5SP216*	RNA, 5S ribosomal pseudogene 216	-1.144E-03
62.	*SNORA26*	small nucleolar RNA, H/ACA box 26	-1.156E-03
63.	*BRSK2*	BR serine/threonine kinase 2	-1.173E-03
64.	*RNA5SP355*	RNA, 5S ribosomal pseudogene 355	-1.178E-03
65.	*KIAA1549L*	KIAA1549 like	-1.223E-03
66.	*SNORD117*	small nucleolar RNA, C/D box 117	-1.228E-03
67.	*RPL3P9*	ribosomal protein L3 pseudogene 9	-1.237E-03
68.	*SNORD1B*	small nucleolar RNA, C/D box 1B	-1.285E-03
69.	*SNHG5*	small nucleolar RNA host gene 5	-1.296E-03
70.	*SNORD103C (SNORD85)*	small nucleolar RNA, C/D box 103C	-1.352E-03
71.	*SNORD103A*	small nucleolar RNA, C/D box 103A	-1.379E-03
72.	*SNORD103B*	small nucleolar RNA, C/D box 103B	-1.417E-03
73.	*SNORD82*	small nucleolar RNA, C/D box 82	-1.448E-03
74.	*SNORA72*	small nucleolar RNA, H/ACA box 72	-1.464E-03
75.	*SNORD91B*	small nucleolar RNA, C/D box 91B	-1.497E-03
76.	*SNORD101*	small nucleolar RNA, C/D box 101	-1.558E-03
77.	*RP11-302F12.1*	Unmatched	-1.588E-03
78.	*MIRLET7F2*	microRNA let-7f-2	-1.620E-03
79.	*SNORD94*	small nucleolar RNA, C/D box 94	-1.642E-03
80.	*PRDM13*	PR/SET domain 13	-1.674E-03
81.	*NPY*	neuropeptide Y	-1.678E-03
82.	*MIR181A1*	microRNA 181a-1	-1.680E-03
83.	*MUC19*	mucin 19, oligomeric	-1.908E-03
84.	*WNK2*	WNK lysine deficient protein kinase 2	-2.018E-03
85.	*SNORD20*	small nucleolar RNA, C/D box 20	-2.069E-03
86.	*RP11-19J5.2*	Unmatched	-2.077E-03
87.	*NHLH2*	nescient helix-loop-helix 2	-2.377E-03
88.	*RNA5SP389*	RNA, 5S ribosomal pseudogene 389	-2.434E-03
89.	*RNA5SP145*	RNA, 5S ribosomal pseudogene 145	-2.567E-03
90.	*RNA5-8SP2*	RNA, 5.8S ribosomal pseudogene 2	-3.226E-03
91.	*RNA5-8SP4*	RNA, 5.8S ribosomal pseudogene 4	-4.066E-03

Table S8. Results of ROC analysis for 51 genes selected as signatures of AAA.

Gene	ROC-AUC[1]	Threshold	Specificity	Sensitivity	Accuracy	Positive Predictive Value	Negative Predictive Value
AC092620.2	1.000	4,752	1,000	1,000	1,000	1,000	1,000
ANKRD13D	1.000	10,690	1,000	1,000	1,000	1,000	1,000
CPT1A	1.000	10,236	1,000	1,000	1,000	1,000	1,000
GGT1	1.000	6,716	1,000	1,000	1,000	1,000	1,000
GIT2	1.000	10,776	1,000	1,000	1,000	1,000	1,000
HTT	1.000	11,316	1,000	1,000	1,000	1,000	1,000
KIAA1549L	0.980	3,411	0,857	1,000	0,929	0,875	1,000
MIR181A1	0.980	3,996	0,857	1,000	0,929	0,875	1,000
MIRLET7F2	1.000	4,995	1,000	1,000	1,000	1,000	1,000
NBEAL2	1.000	12,845	1,000	1,000	1,000	1,000	1,000
PDCD4	1.000	9,950	1,000	1,000	1,000	1,000	1,000
PRDM13	0.959	3,906	0,857	1,000	0,929	0,875	1,000
RN7SKP89	0.980	4,432	0,857	1,000	0,929	0,875	1,000
RNA5SP355	1.000	2,543	1,000	1,000	1,000	1,000	1,000
RNU2-36P	0.959	7,855	0,857	1,000	0,929	0,875	1,000
RP1-102E24.1	0.939	4,629	0,857	1,000	0,929	0,875	1,000
RP11-16F15.2	1.000	4,586	1,000	1,000	1,000	1,000	1,000
RP11-253E3.1	1.000	3,998	1,000	1,000	1,000	1,000	1,000
RP11-302F12.1	1.000	4,509	1,000	1,000	1,000	1,000	1,000
RPL3P9	0.980	4,188	0,857	1,000	0,929	0,875	1,000
SCARNA13	1.000	11,153	1,000	1,000	1,000	1,000	1,000
SCARNA9	1.000	9,994	1,000	1,000	1,000	1,000	1,000
SNHG5	1.000	11,864	1,000	1,000	1,000	1,000	1,000
SNORA12	1.000	9,074	1,000	1,000	1,000	1,000	1,000
SNORA14B	1.000	12,029	1,000	1,000	1,000	1,000	1,000
SNORA26	1.000	11,013	1,000	1,000	1,000	1,000	1,000
SNORA33	1.000	11,001	1,000	1,000	1,000	1,000	1,000
SNORA40	0.959	7,482	1,000	0,857	0,929	1,000	0,875
SNORA50	0.959	9,780	1,000	0,857	0,929	1,000	0,875
SNORA60	1.000	9,636	1,000	1,000	1,000	1,000	1,000
SNORA72	1.000	10,991	1,000	1,000	1,000	1,000	1,000
SNORD101	1.000	9,226	1,000	1,000	1,000	1,000	1,000
SNORD103A	1.000	9,622	1,000	1,000	1,000	1,000	1,000
SNORD103B	1.000	9,567	1,000	1,000	1,000	1,000	1,000
SNORD117	1.000	11,467	1,000	1,000	1,000	1,000	1,000
SNORD119	1.000	9,342	1,000	1,000	1,000	1,000	1,000
SNORD127	1.000	9,717	1,000	1,000	1,000	1,000	1,000
SNORD19	1.000	7,727	1,000	1,000	1,000	1,000	1,000
SNORD1B	0.959	8,096	0,857	1,000	0,929	0,875	1,000
SNORD20	1.000	10,349	1,000	1,000	1,000	1,000	1,000
SNORD82	1.000	10,894	1,000	1,000	1,000	1,000	1,000
SNORD85	0.980	8,779	0,857	1,000	0,929	0,875	1,000
SNORD91B	1.000	10,217	1,000	1,000	1,000	1,000	1,000
SNORD94	1.000	10,895	1,000	1,000	1,000	1,000	1,000
SUFU	1.000	8,666	1,000	1,000	1,000	1,000	1,000
THOC5	1.000	8,940	1,000	1,000	1,000	1,000	1,000
UBE4B	1.000	9,792	1,000	1,000	1,000	1,000	1,000
UPF1	1.000	10,425	1,000	1,000	1,000	1,000	1,000
ZRANB2	1.000	9,058	1,000	1,000	1,000	1,000	1,000
ZSWIM8	1.000	10,435	1,000	1,000	1,000	1,000	1,000
ZZEF1	1.000	11,652	1,000	1,000	1,000	1,000	1,000

[1]Area under ROC curve.

Table S9. Correlation analysis between maximum aneurysm diameter, thrombus volume, aneurysm neck length, age, BMI and expression of 33 selected miRNA transcripts identified as potential AAA signatures.

miRNA transcript	Maximum aneurysm diameter		Thrombus volume		Aneurysm neck length		Age		BMI	
	R	*p*	R	*p*	R	*p*	R	*p*	R	*p*
hsa-let-7g_hsa-let-7g-3p	-0.04	0.859	-0.17	0.381	0.32	0.093	-0.30	0.119	-0.09	0.654
hsa-miR-122-5p	0.10	0.619	0.27	0.160	-0.05	0.782	0.10	0.618	-0.38[1]	0.045
hsa-miR-125b-1-5p	0.02	0.926	-0.19	0.341	0.45[1]	0.015	0.08	0.692	-0.01	0.954
hsa-miR-125b-2-5p	0.12	0.560	-0.08	0.686	0.40[1]	0.037	0.09	0.662	0.02	0.901
hsa-mir-138-1_hsa-miR-138-5p	-0.01	0.944	-0.17	0.389	0.02	0.927	0.13	0.498	-0.21	0.288
hsa-mir-138-2_hsa-miR-138-5p	0.24	0.212	-0.03	0.871	-0.02	0.904	0.15	0.461	-0.29	0.140
hsa-mir-150_hsa-miR-150-5p	0.00	0.993	-0.12	0.540	0.28	0.149	-0.27	0.160	-0.13	0.497
hsa-miR-21-5p	-0.10	0.621	-0.12	0.550	-0.08	0.668	-0.17	0.393	-0.08	0.701
hsa-mir-21_hsa-miR-21-3p	0.31	0.108	-0.28	0.152	-0.25	0.196	0.12	0.535	-0.22	0.262
hsa-mir-24-2_hsa-miR-24-3p	0.09	0.651	-0.07	0.720	-0.21	0.285	0.00	0.989	0.14	0.473
hsa-mir-31_hsa-miR-31-3p	0.16	0.412	-0.10	0.597	0.19	0.326	-0.10	0.627	-0.11	0.566
hsa-mir-31_hsa-miR-31-5p	0.16	0.422	-0.07	0.740	0.22	0.259	-0.02	0.914	-0.02	0.905
hsa-miR-339-3p	0.06	0.751	-0.33	0.085	0.25	0.204	0.00	0.985	-0.05	0.786
hsa-mir-342_hsa-miR-342-3p	-0.08	0.691	-0.22	0.263	0.33	0.088	-0.30	0.119	-0.16	0.416
hsa-miR-34a-3p	0.32	0.092	0.23	0.233	-0.12	0.535	0.08	0.688	0.14	0.479
hsa-miR-34a-5p	0.47[1]	0.011	0.32	0.096	-0.04	0.852	0.26	0.183	-0.01	0.961
hsa-miR-3591-3p	0.10	0.616	0.27	0.160	-0.05	0.781	0.10	0.617	-0.38[1]	0.045
hsa-mir-3607_hsa-miR-3607-5p	0.10	0.605	0.06	0.780	0.21	0.280	-0.26	0.189	-0.06	0.746
hsa-mir-361_hsa-miR-361-3p	0.09	0.633	-0.16	0.404	0.28	0.156	-0.15	0.457	-0.21	0.288
hsa-miR-424-3p	0.29	0.129	0.04	0.833	0.03	0.875	0.12	0.547	-0.08	0.672
hsa-mir-424_hsa-miR-424-5p	0.34	0.073	0.06	0.744	-0.05	0.792	0.21	0.280	-0.17	0.389
hsa-miR-450b-5p	0.20	0.312	0.09	0.636	0.00	0.998	0.01	0.945	-0.17	0.396
hsa-miR-454-3p	0.15	0.449	0.26	0.175	-0.14	0.463	-0.14	0.476	-0.12	0.535
hsa-mir-503_hsa-miR-503-5p	0.27	0.169	-0.01	0.967	-0.01	0.951	0.24	0.212	-0.11	0.586
hsa-miR-542-3p	0.32	0.102	-0.01	0.942	-0.05	0.794	0.27	0.158	-0.21	0.278
hsa-miR-548d-1-3p	0.27	0.159	0.15	0.453	-0.13	0.525	0.05	0.803	-0.08	0.674
hsa-mir-5585_hsa-miR-5585-3p	0.19	0.3226	-0.25	0.195	-0.34	0.080	0.08	0.683	-0.36	0.056
hsa-mir-574_hsa-miR-574-5p	0.16	0.421	0.49[1]	0.007	-0.03	0.896	0.26	0.180	0.16	0.414
hsa-miR-766-3p	-0.19	0.341	-0.09	0.635	-0.05	0.796	0.02	0.917	0.11	0.592
hsa-mir-769_hsa-miR-769-5p	-0.22	0.252	-0.04	0.832	0.36	0.061	-0.41[1]	0.032	0.01	0.973
hsa-mir-7847_hsa-miR-7847-3p	0.33	0.089	-0.01	0.944	0.13	0.521	0.53[1]	0.003	-0.03	0.884
hsa-miR-874-5p	0.27	0.159	-0.20	0.297	-0.03	0.897	-0.01	0.975	-0.14	0.472
hsa-mir-99a_hsa-miR-99a-3p	0.18	0.355	0.13	0.498	0.12	0.553	0.07	0.734	0.21	0.290
Maximum aneurysm diameter	-	-	0.30	0.124	-0.28	0.155	0.42[1]	0.025	-0.09	0.661
Thrombus volume	0.30	0.124	-	-	0.07	0.723	0.00	0.995	0.38[1]	0.046
Aneurysm neck length	-0.28	0.155	0.07	0.723	-	-	-0.13	0.510	0.00	0.983
Age	0.42[1]	0.025	0.00	0.995	-0.13	0.510	-	-	-0.19	0.331
BMI	-0.09	0.661	0.38[1]	0.045	0.00	0.983	-0.19	0.331	-	-

R – Spearman correlation coefficient. [1] correlations statistically significant ($p < 0.05$)

Table S10. Correlation analysis between maximum aneurysm diameter, thrombus volume, aneurysm neck length, age, BMI and expression of 51 selected genes identified as potential AAA signatures.

Gene	Maximum aneurysm diameter		Thrombus volume		Aneurysm neck length		Age		BMI	
	R	*p*	R	*p*	R	*p*	R	*p*	R	*p*
AC092620.2	0.32	0.482	-0.39	0.389	0.69	0.085	0.81	0.028[1]	0.19	0.688
ANKRD13D	0.43	0.335	-0.14	0.766	0.05	0.921	0.66	0.103	0.38	0.399
CPT1A	0.21	0.651	0.47	0.282	0.00	0.998	-0.08	0.866	0.26	0.568
GGT1	-0.07	0.879	-0.58	0.176	0.18	0.695	0.34	0.455	0.72	0.070
GIT2	0.20	0.666	-0.37	0.415	0.64	0.120	0.27	0.563	0.81	0.027[1]
HTT	-0.51	0.242	-0.27	0.560	-0.30	0.516	0.23	0.624	0.35	0.446
KIAA1549L	0.66	0.104	-0.02	0.969	0.38	0.406	0.19	0.691	-0.36	0.431
MIR181A1	0.63	0.130	0.19	0.679	0.01	0.984	0.01	0.989	-0.46	0.296
MIRLET7F2	0.33	0.470	-0.01	0.990	-0.15	0.752	0.50	0.255	-0.33	0.472
NBEAL2	0.06	0.896	-0.19	0.679	-0.12	0.798	0.29	0.528	0.40	0.376
PDCD4	-0.14	0.768	0.03	0.945	-0.07	0.885	-0.81	0.026[1]	-0.18	0.699
PRDM13	0.37	0.420	0.22	0.632	0.14	0.764	-0.09	0.849	-0.71	0.073
RN7SKP89	-0.45	0.310	-0.28	0.537	-0.26	0.569	0.36	0.423	0.25	0.586
RNA5SP355	0.45	0.309	0.48	0.272	0.33	0.472	-0.29	0.529	-0.57	0.183
RNU2-36P	0.48	0.275	0.37	0.415	0.08	0.873	-0.30	0.513	-0.47	0.290
RP1-102E24.1	0.30	0.508	0.38	0.396	0.08	0.864	-0.18	0.703	-0.78	0.039[1]
RP11-16F15.2	0.04	0.927	-0.03	0.945	-0.53	0.226	-0.43	0.337	-0.45	0.317
RP11-253E3.1	0.04	0.931	-0.09	0.841	-0.05	0.915	-0.05	0.909	-0.57	0.185
RP11-302F12.1	0.48	0.271	0.37	0.413	-0.19	0.675	-0.13	0.777	-0.69	0.087
RPL3P9	0.05	0.911	0.14	0.757	-0.42	0.344	-0.17	0.713	-0.76	0.046[1]
SCARNA13	0.24	0.611	0.44	0.321	-0.31	0.496	-0.52	0.231	-0.55	0.202
SCARNA9	0.21	0.647	0.19	0.686	-0.22	0.640	-0.60	0.153	-0.08	0.863
SNHG5	-0.01	0.976	0.32	0.490	-0.22	0.633	-0.80	0.030[1]	-0.19	0.685
SNORA12	0.38	0.394	0.09	0.841	-0.33	0.470	0.00	0.997	-0.45	0.307
SNORA14B	-0.15	0.743	0.31	0.495	-0.53	0.223	-0.66	0.104	-0.35	0.436
SNORA26	0.04	0.925	0.21	0.653	-0.41	0.355	-0.48	0.279	-0.43	0.338
SNORA33	0.29	0.531	0.29	0.532	-0.37	0.407	-0.04	0.930	-0.75	0.053
SNORA40	0.40	0.373	0.00	0.992	-0.07	0.885	-0.35	0.436	-0.24	0.600
SNORA50	0.50	0.252	0.09	0.846	-0.06	0.900	-0.21	0.652	-0.24	0.599
SNORA60	0.01	0.982	-0.20	0.671	-0.38	0.403	-0.44	0.324	0.18	0.700
SNORA72	0.25	0.583	0.29	0.534	-0.20	0.673	-0.34	0.452	-0.55	0.201
SNORD101	0.51	0.242	0.44	0.327	-0.04	0.937	-0.21	0.651	-0.50	0.256
SNORD103A	0.37	0.411	0.20	0.675	-0.15	0.749	-0.31	0.496	-0.45	0.310
SNORD103B	0.35	0.441	0.17	0.716	-0.20	0.664	-0.25	0.590	-0.51	0.245
SNORD117	0.17	0.715	0.33	0.474	-0.33	0.476	-0.33	0.468	-0.28	0.542
SNORD119	0.14	0.771	0.31	0.502	-0.25	0.593	-0.55	0.198	-0.41	0.360
SNORD127	-0.12	0.802	0.11	0.818	-0.37	0.421	-0.42	0.354	-0.29	0.525
SNORD19	0.16	0.735	0.10	0.825	-0.06	0.901	-0.31	0.495	-0.29	0.525
SNORD1B	0.45	0.307	0.16	0.726	-0.05	0.911	-0.29	0.524	-0.42	0.350
SNORD20	0.03	0.954	0.26	0.567	0.00	0.994	-0.67	0.101	-0.27	0.562
SNORD82	0.26	0.579	0.22	0.639	-0.24	0.603	-0.41	0.357	-0.36	0.425
SNORD85	0.29	0.532	0.39	0.384	-0.12	0.792	-0.40	0.377	-0.38	0.399
SNORD91B	0.09	0.845	0.11	0.819	-0.14	0.772	-0.62	0.134	-0.13	0.788
SNORD94	-0.12	0.791	0.39	0.389	-0.39	0.392	-0.71	0.076	-0.02	0.963
SUFU	0.48	0.278	-0.24	0.597	0.48	0.274	0.77	0.041[1]	0.41	0.357
THOC5	-0.08	0.870	0.22	0.638	0.41	0.357	0.12	0.791	0.52	0.234
UBE4B	-0.42	0.354	-0.67	0.096	0.26	0.573	0.46	0.302	0.49	0.270
UPF1	0.11	0.819	0.29	0.534	-0.14	0.767	-0.02	0.960	0.37	0.418
ZRANB2	-0.35	0.448	-0.19	0.679	-0.21	0.649	-0.79	0.036[1]	0.08	0.857
ZSWIM8	0.00	0.993	0.25	0.590	-0.22	0.642	0.00	0.995	0.39	0.392
ZZEF1	-0.39	0.388	-0.14	0.757	-0.13	0.788	0.00	0.995	0.65	0.116
Maximum aneurysm diameter	-	-	0.40	0.371	0.51	0.240	0.29	0.525	-0.04	0.928

Thrombus volume	0.40	0.371	-	-	0.03	0.943	-0.19	0.688	0.40	0.375
Aneurysm neck length	0.51	0.240	0.03	0.943	-	-	0.24	0.601	0.28	0.543
Age	0.29	0.525	-0.19	0.688	0.24	0.601	-	-	-0.16	0.730
BMI	-0.04	0.928	-0.40	0.375	0.28	0.543	-0.16	0.730	-	-

R – Spearman correlation coefficient. [1] correlations statistically significant ($p < 0.05$)

Table S11: Experimentally validated miRNA:gene pairs found *in silico* among 31 miRNAs and 51 genes indicative for AAA.

	Database	miRNA ID	Gene symbol	Method
1.	mirtarbase, tarbase	hsa-miR-21-5p	*PDCD4*	Western blot, Northern blot, activity assay, Luciferase reporter assay, Immunoblot analysis, qRT-PCR, Quantitative proteomic approach, GFP reporter assay, ASO assay, Immunohistochemistry, microarray, Sequencing, PAR-CLIP, In situ hybridization, reporter assay
2.	mirtarbase	hsa-miR-150-5p	*PDCD4*	HITS-CLIP
3.		hsa-miR-150-5p	*CPT1A*	HITS-CLIP
4.		hsa-miR-24-3p	*CPT1A*	HITS-CLIP
5.		hsa-miR-574-5p	*KIAA1549L*	PAR-CLIP

PAR-CLIP – Photoactivatable Ribonucleoside-Enhanced Crosslinking and Immunoprecipitation, HITS-CLIP – High-Throughput Sequencing of RNA isolated by Cross-Linking Immunoprecipitation, qRT-PCR – Quantitative Reverse-Transcriptase Polymerase Chain Reaction, GFP – Green Fluorescent Protein, ASO – Antisense Oligonucleotide.

Table S12: Top 10% predicted miRNA:gene pairs obtained *in silico* among 31 miRNAs and 51 genes found as signatures of AAA.

	Database	miRNA ID	Gene symbol	Database-specific probability value
1.	diana_microt	hsa-miR-21-5p	*PDCD4*	1
	elmmo			0.751
	miranda			-1.1999
	targetscan			-0.481
2.	diana_microt	hsa-miR-424-5p	*UBE4B*	0.994
	elmmo			0.785
	miranda			-1.1586
	mirdb			99.467306
	targetscan			-0.499
3.	diana_microt	hsa-miR-138-5p	*THOC5*	0.964
	pita			-18.96
	targetscan			-0.442
	miranda			-0.9972
4.	diana_microt	hsa-miR-125b-5p	*NBEAL2*	0.942
	elmmo			0.775
	microcosm			17.624
5.	diana_microt	hsa-miR-424-5p	*PDCD4*	0.941
	elmmo			0.811
				0.702
	targetscan			-0.261
6.	diana_microt	hsa-miR-150-5p	*PDCD4*	0.926
7.		hsa-miR-503-5p	*UBE4B*	0.888
	elmmo			0.702
8.	diana_microt	hsa-let-7g-3p	*UBE4B*	0.88
	elmmo			0.705
9.	diana_microt	hsa-miR-34a-5p	*PDCD4*	0.878
	elmmo			0.512
	pita			-15.09
10.	diana_microt	hsa-miR-342-3p	*UPF1*	0.859
11.		hsa-miR-21-3p	*UBE4B*	0.829
	miranda			-1.2926
	mirdb			98.49234
	targetscan			-0.333
12.	diana_microt	hsa-miR-24-3p	*KIAA1549L*	0.821
	pita			-13.96
13.	diana_microt	hsa-miR-503-5p	*PDCD4*	0.816
	targetscan			-0.278
14.	diana_microt	hsa-miR-125b-5p	*THOC5*	0.809
15.		hsa-miR-3591-3p	*UBE4B*	0.809
	mirdb			98.49234
	targetscan			-0.346
16.	diana_microt	hsa-miR-342-3p	*UBE4B*	0.804
17.	elmmo	hsa-miR-424-5p	*ANKRD13D*	0.628

	targetscan			-0.3
18.	elmmo	hsa-miR-454-3p	*UBE4B*	0.792
	pita			-11.37
19.	elmmo	hsa-miR-450b-5p	*KIAA1549L*	0.583
20.		hsa-miR-450b-5p	*GIT2*	0.56
21.		hsa-miR-34a-5p	*PRDM13*	0.549
22.		hsa-miR-450b-5p	*CPT1A*	0.518
23.	microcosm	hsa-miR-361-3p	*GGT1*	17.5354
	targetscan			-0.415
24.	miranda	hsa-miR-542-3p	*ZRANB2*	-1.0109
				-1.0288
25.		hsa-miR-34a-3p	*SNORA60*	-1.0324
26.		hsa-miR-769-5p	*PDCD4*	-1.0833
	targetscan			-0.267
27.	miranda	hsa-miR-548d-3p	*ANKRD13D*	-1.0874
28.		hsa-miR-24-3p	*SNORD94*	-1.1126
29.		hsa-miR-21-5p	*ZRANB2*	-1.1294
				-1.1426
30.		hsa-miR-548d-3p	*PDCD4*	-1.1607
31.		hsa-miR-31-5p	*SUFU*	-1.2842
	pita			-16.11
32.	mirdb	hsa-miR-874-5p	*ZSWIM8*	92.75364
	targetscan			-0.722
33.	pita	hsa-miR-769-5p	*SUFU*	-10.68
				-16.77
34.		hsa-miR-542-3p	*GIT2*	-10.74
35.		hsa-miR-34a-5p	*SUFU*	-10.79
36.		hsa-miR-31-5p	*CPT1A*	-11.05
37.		hsa-miR-574-5p	*KIAA1549L*	-11.31
				-11.35
				-18.44
38.		hsa-miR-503-5p	*ANKRD13D*	-11.42
39.		hsa-miR-31-5p	*KIAA1549L*	-11.5
40.		hsa-miR-122-5p	*PDCD4*	-11.53
41.		hsa-miR-122-5p	*KIAA1549L*	-11.54
42.		hsa-miR-769-5p	*HTT*	-11.67
				-11.88
43.		hsa-miR-339-3p	*GIT2*	-11.95
44.		hsa-miR-138-5p	*KIAA1549L*	-11.99
				-14.49
				-14.5
45.		hsa-miR-503-5p	*HTT*	-12.03
46.		hsa-miR-31-5p	*ZZEF1*	-12.04
47.		hsa-miR-24-3p	*ANKRD13D*	-12.86
48.		hsa-miR-24-3p	*HTT*	-13.35
				-13.68
49.		hsa-miR-766-3p	*ANKRD13D*	-13.37
50.		hsa-miR-34a-5p	*ZZEF1*	-13.51

51.		hsa-miR-122-5p	*SUFU*	-13.66
52.		hsa-miR-766-3p	*SUFU*	-13.79
53.		hsa-miR-34a-5p	*UBE4B*	-13.87
54.		hsa-miR-138-5p	*SUFU*	-13.95
				-14.02
				-14.07
55.		hsa-miR-361-3p	*ZRANB2*	-14.36
56.		hsa-miR-138-5p	*HTT*	-15.34
57.		hsa-miR-150-5p	*UBE4B*	-15.9
58.		hsa-miR-766-3p	*ZZEF1*	-17.19
59.	targetscan	hsa-miR-874-5p	*ANKRD13D*	-0.285
60.		hsa-miR-7847-3p	*UBE4B*	-0.379
